# The Architecture of Immune Escape in Neuroblastoma: Plasticity, Silence and Escape Engineer Immune Blindness

**DOI:** 10.3390/cells15121072

**Published:** 2026-06-12

**Authors:** Poorvi Subramanian, Loganayaki Periyasamy, Sreenidhi Mohanvelu, Sheeja Aravindan, Natarajan Aravindan

**Affiliations:** 1 Department of Physiological Sciences, College of Veterinary Medicine, Oklahoma State University, Stillwater, OK 74078, USA; poorvi.subramanian@okstate.edu (P.S.); loganayaki.periyasamy@okstate.edu (L.P.); sreenidhi.mohanvelu@okstate.edu (S.M.); 2 OU Health Stephenson Cancer Center, Oklahoma City, OK 73104, USA; sheeja-aravindan@ou.edu; 3 Departments of Radiation Oncology and Pathology, University of Oklahoma Health Sciences Center, Oklahoma City, OK 73104, USA

**Keywords:** high-risk neuroblastoma, tumor immune evasion, tumor microenvironment, immune checkpoint blockade, immune cell exhaustion, epigenetic reprogramming, myeloid-derived suppressor cells, tumor derived exosomes, metabolic reprogramming, CAR-therapies

## Abstract

Neuroblastoma (NB), the most common extracranial solid tumor of childhood, exemplifies one of the most formidable paradigms of tumor immune evasion (TIME) in pediatric oncology. Despite significant advances in multimodal therapy and the clinical integration of immunotherapeutic strategies, high-risk NB (HR-NB) remains largely refractory to durable immune control. This failure reflects not an absence of immune engagement, but the presence of a highly evolved and developmentally wired immune escape architecture. In this review, we synthesize emerging insights from single-cell, multi-omics, and functional studies to define how developmental lineage, cellular plasticity, metabolic rewiring, epigenetic regulation, and therapy-induced adaptation converge to engineer immune blindness in NB. We discuss how NB’s neural crest origin establishes a baseline of low immunogenicity, which is subsequently reinforced through coordinated suppression of antigen presentation, dominance of immune checkpoint signaling, and profound dysfunction of cytotoxic T and natural killer cells within an immunosuppressive tumor microenvironment. Central to this process is tumor-intrinsic plasticity, whereby lineage instability and dedifferentiation, exacerbated by therapeutic pressure, embed immune silence as a stable tumor state. We highlight evidence positioning RD3 as a master upstream regulator linking cellular identity to immune visibility, governing antigen presentation, innate immune sensing, checkpoint expression, and cytotoxic lymphocyte engagement. Beyond tumor-intrinsic mechanisms, we examine the roles of immunosuppressive myeloid populations, tumor-derived exosomes, metabolic stress, hypoxia, and ferroptosis-associated pathways in reinforcing immune paralysis. Finally, we outline emerging therapeutic strategies aimed at dismantling this architecture, including combinatorial checkpoint blockade, metabolic and epigenetic reprogramming, exosome-targeted interventions, and next-generation immune engineering platforms. Together, this review reframes TIME in NB as a programmable, developmentally rooted process and provides a mechanistic roadmap for restoring immune competence and therapeutic susceptibility in HR disease.

## 1. Introduction

### Neuroblastoma Clinical Heterogeneity

Neuroblastoma (NB) is the most common extracranial solid tumor of childhood and remains a major clinical challenge within pediatric oncology. It accounts for significant childhood cancer morbidity and mortality, representing ~15% of all childhood cancer-related deaths [1]. As an embryonal malignancy arising from neural crest cell (NCC)-derived sympathetic lineage cells, NB exhibits a uniquely broad clinical spectrum, ranging from benign lesions that spontaneously regress to aggressive metastatic disease that is often refractory to therapy. This dramatic variability in NB behavior underscores its biological complexity and drives ongoing efforts to better understand the mechanisms underlying its progression and treatment response [1,2]. NB primarily affects very young children, especially during infancy and early toddlerhood (median age 22 months). Its early onset reflects the tumor’s origin during fetal development, where aberrant differentiation and proliferation of sympathetic precursor cells can give rise to malignancy. Interestingly, unlike many pediatric cancers that display relatively uniform behavior across patients, NB is characterized by striking heterogeneity both clinically and molecularly [3]. Some infants with localized disease may experience spontaneous tumor regression, a phenomenon rarely observed in human cancers, whereas others present with widespread metastatic involvement, often associated with poor prognosis. This heterogeneity is compounded by the diversity in genetic and epigenetic landscapes observed across patient tumors [3]. Although NB has a relatively low mutational burden when compared with adult solid tumors, specific genomic alterations have profound implications for disease behavior. Among the most clinically important features is MYCN amplification, a HR genetic marker strongly associated with rapid tumor progression, metastatic dissemination, and poor survival outcomes. MYCN-amplified tumors represent one of the most aggressive NB subtypes, contributing significantly to treatment resistance and high mortality rates in older children [4,5]. Beyond MYCN, other signaling pathways, including ALK, RAS-MAPK, PI3K/AKT/mTOR, and various epigenetic regulators, contribute to the variable biology of NB and influence clinical trajectories [6,7].

**Figure 1:**
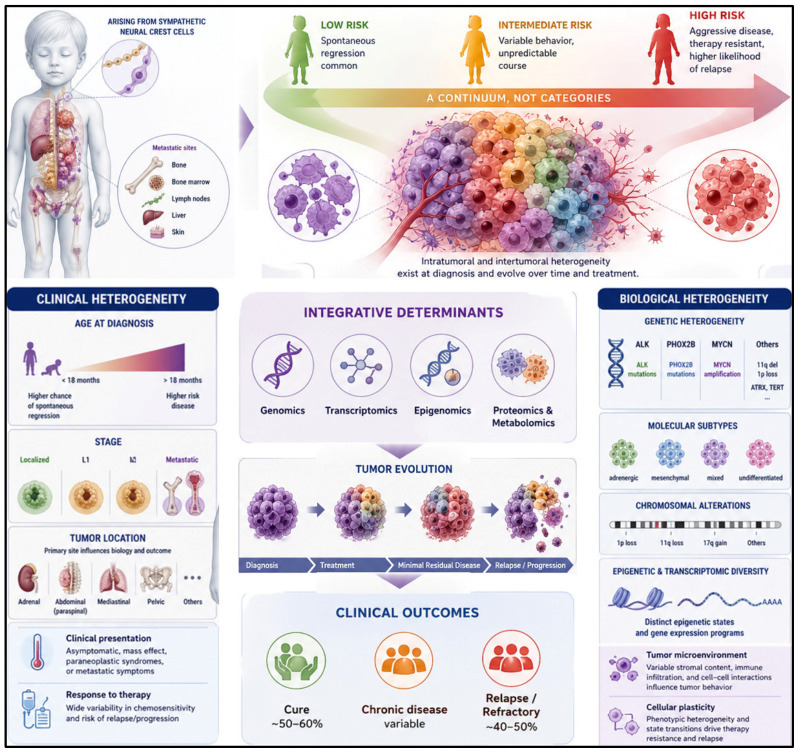
***Clinical heterogeneity of NB.*** NB is not a single disease but a biologically and clinically heterogeneous group of tumors. It is characterized by dynamic risk at diagnosis, variable responses to treatment, and a broad spectrum of clinical outcomes. A comprehensive understanding of this heterogeneity, through integrated molecular profiling, minimal residual disease monitoring, functional assays, single-cell approaches, and the development of novel targeted immunotherapies, is essential to improving outcomes for every child.

NB’s clinical behavior is shaped by hallmarks such as angiogenesis, apoptosis dysregulation, cell cycle alterations, drug resistance, hypoxia responses, and reactive oxygen species (ROS) signaling, the molecular features not only central to tumor progression but also dictate differential treatment responsiveness and play pivotal role in survival outcomes [7]. In particular, the interaction between tumor cells and their surrounding microenvironment has gained recognition as a key modulator of tumor aggressiveness and immune escape. The tumor microenvironment (TME) of NB plays an essential role in promoting clinical heterogeneity [8]. NB tumors frequently harbor immunosuppressive TME, with limited infiltration of cytotoxic immune cells and the presence of immunoregulatory components that inhibit effective antitumor immunity. Tumor-associated macrophages (TAMs, myeloid-derived suppressor cells (MDSCs), regulatory T cells (TREGs), and stromal populations collectively create a niche that facilitates tumor survival and metastasis. Notably, HR NB (HR-NB) tumors often demonstrate infiltrates of dysfunctional or exhausted T_c_ and natural killer (NK) cells, highlighting the immune-evasive adaption of aggressive disease forms. This immune dysfunction is further shaped by tumor-derived exosomes (TDEs) and metabolic shifts within the TME, which contribute to impaired dendritic cell (DC) maturation, suppression of NK cytotoxicity, and expansion of immunosuppressive cell subsets [9,10]. This complexity is reflected in treatment approaches, which must account for the diverse clinical presentations of NB. Historically, management of HR-NB has included multimodal therapies involving chemotherapy (CT), surgery, radiation therapy (RT), autologous stem cell rescue (ASCT), and differentiating agents such as isotretinoin. Over the past decade, the incorporation of immunotherapy (IMT), most prominently anti-GD2 monoclonal antibodies, has marked a major advance in improving survival rates for HR patients. Yet despite these advancements, outcomes for many children with HR or relapsed disease remain poor, with long-term (10-Year) overall survival (OS) rates still hovering below 10% in (HR-NB) cohorts. The persistence of treatment-resistant disease highlights the critical need to better understand the biological drivers of NB heterogeneity, particularly immune evasion mechanisms that undermine IMTs [11,12,13]. Cutting-edge technologies such as single-cell RNA sequencing further underscore the diversity within NB tumors, revealing substantial variation in immune infiltrates and transcriptional programs across both treatment-naïve and post-chemotherapy specimens. Recent analyses demonstrate the presence of at least 17 distinct immune cell subsets within NB tumors and identify the NECTIN2–TIGIT axis as a key checkpoint pathway capable of suppressing antitumor responses. Such findings offer insight into why some patients derive substantial benefit from IMT while others show suboptimal responses or develop rapid resistance [12]. Together, the clinical and biological heterogeneity of NB necessitates personalized and risk-adapted treatment strategies, integrating genomic profiling, immune characterization, and novel targeted therapeutics (Figure 1).

## 2. IMT and the Challenge of Immune Evasion in NB

In the last decade, IMT has emerged as a transformative addition to frontline treatment regimens, offering a powerful means of enhancing antitumor activity through immune-mediated cytotoxicity. However, its success remains limited by the profound ability of NB tumors to evade immune detection and suppress immune function. Understanding both the promise of IMT and the biological obstacles imposed by tumor immune evasion (TIME) is essential for advancing treatment strategies capable of improving survival in this devastating disease [13].

### 2.1. IMT as a Critical Advancement in NB Treatment

The integration of immunotherapeutic strategies into standard NB care, in particular anti-GD2 monoclonal antibodies, such as dinutuximab, have played a central role in enhancing survival outcomes for children with HR-NB. GD2 is a disialoganglioside abundantly expressed on NB cells, and its restricted expression on normal tissues makes it an excellent target for antibody-mediated cytotoxicity (ADCC). Indeed, survival improvements following adoption of anti-GD2 therapy are widely recognized and have demonstrated the potential of immune-based approaches to address minimal residual disease (MRD) after intensive CT and consolidation therapy [12]. Beyond anti-GD2 therapy, a broader interest in next-generation immunotherapeutics, including CAR-T cell therapy, immune checkpoint inhibitors, engineered NK cell therapies, and exosome-based immune modulation, has emerged as a means to more effectively redirect or enhance antitumor immune activity. As the NB immunobiology supports multiple immunotherapeutic targets due to its expression of cell-surface antigens, its susceptibility to NK-cell cytotoxicity, and its interactions with immune-regulatory pathways that can be pharmacologically modulated, IMT is increasingly appreciated as a central pillar in the future of NB treatment design [13]. However, the full realization of IMT approaches has been tempered by the formidable immune-evasive capabilities of NB. Despite the demonstrated efficacy of anti-GD2 IMT, treatment failure and relapse remain significant challenges. The deeper the field explores IMT, the more evident it becomes that NB employs a sophisticated network of immune escape mechanisms, many of which are now being uncovered by advanced genomics, epigenomics, and single-cell technologies.

### 2.2. Biological Basis of Immune Evasion in NB

**Figure 2:**
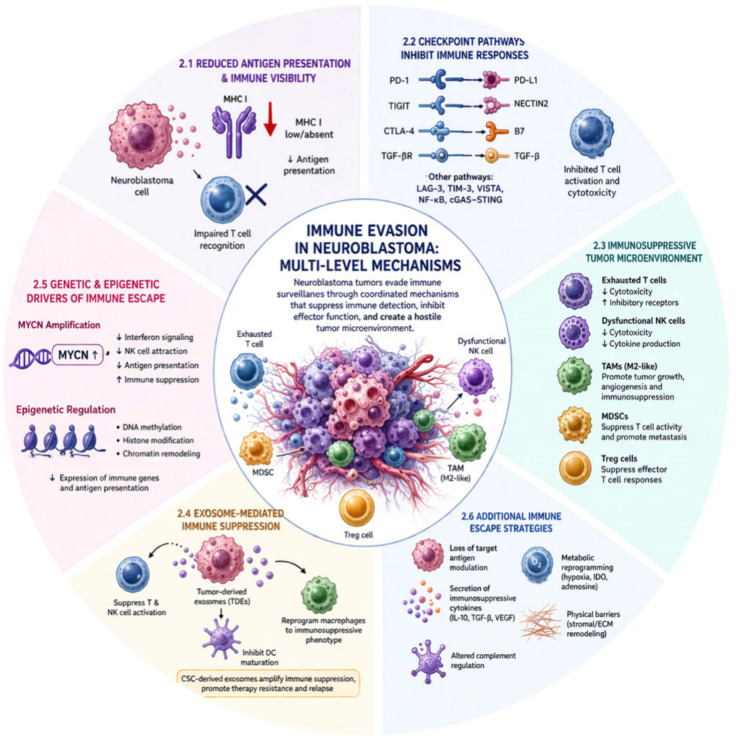
***Biological basis of immune evasion in NB.*** Immune evasion wiring in NB is multifold including reduced antigen presentation and immune visibility; immune checkpoints; immunosuppressive tumor microenvironment; exosomes based immune suppression; genetic and epigenetic reprogramming; metabolic reprograming; physical barriers etc.

Immune evasion is not simply an ancillary feature of NB biology, it is central to tumor survival, especially in HR and relapsed disease. Several converging forms of evidence demonstrate that NB tumors actively remodel the immune microenvironment, suppress immune effector cell function, reduce antigen visibility, and engage ligand–receptor pathways that inhibit cytotoxic attack (Figure 2).

*Reduced Antigen Presentation and Immune Visibility*: One of the most widely recognized immune-evasion strategies involves the downregulation of antigen presentation machinery, including major histocompatibility complex (MHC) class I molecules. Although NB is traditionally considered to express low levels of MHC-I even in early development, tumor-driven suppression further reduces T_c_ recognition. In addition, IL-10 contributes to immunosuppressive TME by inhibiting antigen presentation and suppressing effector T_c_ responses, thereby facilitating immune escape. These mechanisms mirrors broader themes identified in cancer biology, where antigen-presentation loss is a hallmark of reduced immune surveillance [14].
*Immune Checkpoint Engagement as a Barrier to Cytotoxic Responses*: NB cells also leverage multiple inhibitory checkpoint pathways to dampen immune activity. While the PD-1/PD-L1 axis is well established, recent mechanistic analyses reveal that other checkpoint circuits (e.g., NECTIN2–TIGIT axis) are particularly important in NB (Table 1) [12]. A landmark bed-to-bench study identified Retinal degeneration protein 3 (RD3) as a dominant immune-suppressive pathway in NB, where therapy-pressure steered RD3-depletion determines immune cell type composition in the NB-TME [15]. RD3-dependent 27-gene signature dramatically improving immune-mediated tumor clearance in preclinical models, and RD3-loss contributed to the TIME with impeded homing of activated-CD4^+^ and -CD8^+^ T_c_ [15]. More broadly, other immunosuppressive pathways (CTLA-4, TGF-β, NFκB, cGAS–STING axis), have been implicated in shaping the local immune environment and reinforcing tumor tolerance. In addition, IL-2 is important for T-cell proliferation and activation and has been used clinically to enhance anti-tumor immune responses, particularly in combination with IMTs such as anti-GD2 antibodies. These findings, although not all are equally dominant, not only contribute to the layered network of immune suppression that challenges IMT efficacy, but also, underscore the essential role of immune checkpoint interactions in NB immune evasion and highlight the need for combination-based IMT [12,14].

### 2.3. Immunosuppressive TME in NB

A key obstacle to successful IMT lies in the composition and function of the NB TME. NB tumors create a deeply immunosuppressive niche that dampens immune function through cellular, molecular, and metabolic mechanisms. NB tumors frequently contain infiltrates of dysfunctional or exhausted T and NK cells, which display reduced cytotoxic potential and impaired cytokine production [8]. Single-cell profiling of NB demonstrates that NK cells in particular exhibit suppressed cytotoxic signatures, and T_c_ exhibit exhaustion phenotypes both before and after CT, explaining why even NB that express targetable surface antigens may still escape immune-mediated destruction [12]. In addition, the NB TME also contains abundant immunosuppressive myeloid populations, including TAMs and MDSCs, those that further suppress T_c_ function, promote tumor growth, and facilitate metastatic progression [10]. Their presence is closely tied to poor prognosis and is a major contributor to the failure of IMT in HR disease states [12]. One of the more recently uncovered mechanisms of TIME involves TDEs that transport proteins, RNA, and lipids and modulate immune activity at both local and systemic levels. In NB, TDEs suppress T and NK cell activation, inhibit DC maturation, and reprogram macrophages toward immunosuppressive phenotypes. Cancer stem cell (CSC)-derived exosomes amplify these effects, contributing to therapeutic resistance and promoting relapse. Notably, inhibition of exosome release or uptake significantly enhances immune-based treatments, making them increasingly attractive as therapeutic targets [29].

### 2.4. Genetic and Epigenetic Influences on Immune Escape

The genomic drivers of NB also influence immune dynamics. MYCN amplification, perhaps the most important oncogenic determinant of HR-NB, has profound effects on immune function, contributing to reduced immunogenicity, impaired antigen presentation, and enhanced immune suppression [5]. HR-NB tumors with MYCN amplification show deeply immunosuppressive TMEs, and MYCN overexpression has been linked to reduced interferon signaling and diminished NK cell attraction, undermining immune surveillance. These features align with broader observations that MYCN and other oncogenic pathways sculpt an immune-resistant tumor phenotype [24,30]. Epigenetic regulators, such as DNA methylation machinery, histone modifiers, and chromatin remodeling complexes, also contribute to NB immune evasion by repressing antigen-presentation genes, altering cytokine networks, and promoting tumor plasticity. Modern studies highlight the interplay between the NB epigenome, microenvironment, and immune system, emphasizing epigenetic therapeutics as potential tools for reversing immune resistance and sensitizing tumors to IMT [31].

### 2.5. The Clinical Consequences of Immune Evasion in NB

The ability of NB tumors to evade immune surveillance has profound implications for therapeutic success. Even when initial tumor burden is reduced by cytotoxic CT, residual disease may persist due to immune escape mechanisms that prevent the body’s natural defenses from eliminating remaining tumor cells. This is a primary contributor to relapse, which remains the greatest clinical barrier in HR-NB management [32,33]. Moreover, immune evasion directly undermines the efficacy of immunotherapeutic strategies: (a) Anti-GD2 therapies are limited when tumors suppress NK-cell function or downregulate complement-mediated cytotoxic pathways; (b) CAR-T cell therapies fail when tumors exclude T_c_, suppress T_c_ activation, or remodel the TME to promote exhaustion; and (c) Checkpoint inhibitors have limited efficacy unless paired with strategies that enhance immune infiltration or overcome suppressive TMEs. Thus, the challenge is not simply developing new immune-based treatments but designing combination strategies that can simultaneously activating effector cells and blocking suppressive pathways, circumventing tumor immune avoidance [32]. Future directions must integrate single-cell technologies, epigenetic targeting, metabolic reprogramming, exosome inhibition, and multi-checkpoint interference in order to fully unlock the potential of IMT for children affected by aggressive NB.

## 3. NB TME and Immune Landscape

The TME of NB plays a pivotal role in dictating disease aggressiveness, therapeutic resistance, metastatic behavior, and responsiveness to IMT. Unlike many adult cancers, NB arises during early development from NCC-derived sympathetic progenitors, resulting in a unique interaction between tumor cells and the developing immune system. Advanced technologies such as scRNA-seq have revealed that NB tumors are infiltrated by a diverse ecosystem of immune and stromal cell populations, each contributing to a finely tuned balance between immune activation and immune suppression [12,34]. Recent high-resolution immune profiling studies show at least 17 distinct immune subsets infiltrating NB, reflecting a TME of remarkable complexity and heterogeneity [12,15]. This section provides an in-depth review of the cellular constituents of the NB TME, focusing on NK cells, T_c_, B cells (B_c_), and myeloid cells, and elaborates on the immunosuppressive mechanisms that shape tumor progression, immune evasion, and therapeutic failure.

### 3.1. Cellular Components of the NB TME

#### 3.1.1. NK Cells

NK cells play an essential role in innate immune surveillance, particularly against tumors that downregulate MHC class I, as NB commonly does. NB are notably infiltrated by NK cell populations; however, multiple studies show that NK cells in the NB TME exhibit reduced cytotoxicity and impaired effector function, especially in HR-NB. NK cells demonstrate suppressed cytotoxic signatures, including diminished expression of perforin and granzyme molecules, and an exhausted or dysfunctional phenotype that fluctuates during and after CT [35,36]. Several mechanisms contribute to NK cell dysfunction in NB including upregulation of inhibitory ligands such as NECTIN2 on tumor cells, which engage inhibitory receptors like TIGIT on NK cells. The NECTIN2–TIGIT interaction was identified as a crucial immune checkpoint that actively suppresses NK cell activation in NB [12]; TDEs that inhibit NK cell proliferation, cytotoxic function, and cytokine production, thereby diminishing NK-mediated immunosurveillance [29]; metabolic constraints within the TME, such as hypoxia, nutrient depletion, and lactic acid accumulation, which further suppress NK activity [14]. Given these immunosuppressive influences, therapeutic strategies that enhance NK cell function, such as TIGIT blockade, NK-cell engaging antibodies, and engineered NK-derived exosomes, represent promising avenues for restoring NK-mediated tumor clearance.

#### 3.1.2. T Cells

T_c_ constitute another critical component of the immune infiltrate in NB, yet their anti-tumor function is severely compromised. CD8^+^ cytotoxic T lymphocytes (CTLs) are central mediators of anti-tumor immunity in NB through their ability to recognize tumor-associated antigens presented on MHC class I molecules and directly lyse malignant cells. However, their effectiveness is markedly constrained in NB due to intrinsic and microenvironmental immune evasion mechanisms. NB cells frequently exhibit low or absent MHC class I expression and antigen-processing machinery, limiting CTL recognition and activation. In addition, the TME is characterized by low immunogenicity, sparse T_C_ infiltration, and the presence of immunosuppressive cytokines and cells, all of which impair CD8^+^ T_C_ priming and effector function. Tumor-infiltrating CD8^+^ T_Cs_ often display a dysfunctional or exhausted phenotype, driven in part by immune checkpoint pathways such as PD-1/PD-L1 and TIGIT, further reducing cytotoxic activity. Despite these barriers, higher levels of cytotoxic T_C_ infiltration correlate with improved patient outcomes, underscoring their therapeutic relevance. Consequently, strategies aimed at restoring antigen presentation, reversing T_C_ exhaustion, and remodeling the TME are critical to enhancing CD8^+^ T_C_-mediated immunity and overcoming immune escape in NB.

Multiple studies highlight the presence of dysfunctional, exhausted, or metabolically impaired T_c_ populations in NB tumors [15,37]. scRNA-seq analyses reveal T_c_ subsets exhibiting classical exhaustion markers, reduced effector cytokine profiles, and suppressed cytotoxic potential that worsen after CT exposure [12,34,37]. Key features of T_c_ dysfunction in the NB TME include expression of inhibitory checkpoint receptors, especially TIGIT and PD-1, which directly suppress T_c_ activation and proliferation. Combination blockade of TIGIT and PD-L1 has shown synergy in restoring T_c_ function and reducing NB tumor growth in vivo, leading to complete responses in preclinical models [12]; exosome-mediated suppression, where NB-derived exosomes inhibit T_c_ activation and promote expansion of suppressive T_c_ subsets while reducing antitumor T_c_ signaling pathways [29]; suppression through myeloid cell interactions, as TAMs and MDSCs secrete inhibitory cytokines and metabolites that promote T_c_ exhaustion. Further compounding the issue, NB often exhibits low antigenicity, partially due to reduced expression of MHC class I, ultimately diminishing the ability of T_c_ to recognize and eliminate tumor cells, a common immune evasion mechanism across cancers [14,38].

#### 3.1.3. B Cells

Although less studied than T_c_ or NK cells, B_c_ do infiltrate NB tumors and may play diverse roles in modulating immune responses. scRNA-seq data from primary NB tumors identifies B_c_ subsets among the 17 infiltrating immune populations [39]. Their functional contribution is complex and can include antigen presentation to T_c_, which may be impaired due to TME conditions; production of antibodies, although most NB tumors lack strong neoantigens for robust B_c_ stimulation; regulatory B_c_ (Breg)-mediated immune suppression, which can contribute to a tolerogenic microenvironment [39,40]. While B_c_ do not appear to be the dominant immune population in NB, emerging evidence suggests they may participate in shaping T_c_ responses and influencing the broader tumor immune network.

#### 3.1.4. Myeloid (TAMs, MDSCs, DCs) Cells

The most influential immune cell populations in the NB TME are myeloid lineage cells, which include TAMs, MDSCs, and dysfunctional DCs. These cells form a cornerstone of TIME strategies and are strongly associated with poor clinical outcomes.

TAMs: NB tumors contain abundant TAMs that support tumor growth, angiogenesis, metastasis, and immunosuppression. In the NB TME, macrophages are frequently polarized toward an M2-like immunosuppressive phenotype, characterized by secretion of anti-inflammatory cytokines (IL-10, TGF-β), promotion of tumor cell survival and invasion and suppression of effector T_c_ responses. scRNA-seq studies confirm the presence of strong immunosuppressive myeloid signatures across NB tumors, contributing to dysfunctional antitumor immunity [19].
MDSCs: MDSCs are potent immune suppressors that inhibit T_c_, NK cells, and antigen-presenting cells. Their recruitment and expansion in NB are supported by TDEs, inflammatory factors, and metabolic alterations. MDSCs impair multiple arms of the immune response by producing nitric oxide (NO) and ROS, secreting arginase-1 and depleting nutrients essential for lymphocyte activation and promoting B_c_ exhaustion [10].
DCs: In NB, TDEs inhibit DC maturation, which prevents effective antigen presentation and T_c_ priming. This effect creates a bottleneck in adaptive immunity, further contributing to tolerogenic TME. Studies highlight that blocking the release or uptake of TDEs can partially restore DC function and enhance IMT effectiveness [29].

### 3.2. Immunosuppressive Features of the NB TME

The NB TME is profoundly immunosuppressive, driven by cellular, molecular, metabolic, and vesicle-mediated mechanisms. This constellation of suppressive influences creates one of the most challenging barriers to successful IMT in pediatric solid tumors.

#### 3.2.1. Immune Checkpoint Signaling and Ligand–Receptor Suppression

NB employs a complex array of immune checkpoint signaling mechanisms to suppress antitumor immunity, effectively dampening both T_c_- and NK cell-mediated responses within the TME. Among these pathways, the NECTIN2–TIGIT axis has emerged as a central inhibitory checkpoint in NB, where engagement of TIGIT on cytotoxic lymphocytes (CTL) potently restrains their effector function; notably, experimental blockade of this axis markedly enhances NK and T_c_ activity and has been shown to induce complete tumor regression in vivo, underscoring its functional dominance in TIME [12]. In parallel, the PD-1/PD-L1 axis plays a critical role, as PD-L1 expression is frequently upregulated in NB following CT or inflammatory conditioning, leading to T_c_ cell exhaustion, impaired cytotoxicity, and immune escape in a manner consistent with established paradigms across multiple cancer types [41]. Additional immunoregulatory pathways, including CTLA-4, TGF-β signaling, NFκB-driven transcriptional programs, and dysregulation of the cGAS–STING pathway, further contribute to immune suppression by shaping cytokine landscapes, inhibiting antigen presentation, and attenuating innate immune sensing [19,42]. Recently, two seminal studies extend the immune checkpoint framework in NB by integrating tumor-intrinsic differentiation programs, most notably those controlled by RD3, with microenvironment-mediated immune suppression [15,37]. Both reports emphasize that NB TIME is not solely driven by extrinsic checkpoint ligands but is tightly coupled to the differentiation state of tumor cells and their transcriptional wiring. RD3, identified in NB as a differentiation-associated tumor suppressor, emerges as a critical molecular brake on aggressive tumor behavior. Functionally, RD3 promotes neuronal differentiation and restrains proliferative and invasive programs, thereby limiting the emergence of poorly differentiated, HR tumor states that are most adept at immune escape. Loss or downregulation of RD3 shifts NB cells toward an undifferentiated phenotype characterized by heightened inflammatory signaling, activation of NFκB and TGF-β pathways, and increased expression of immune inhibitory molecules, including checkpoint ligands induced under therapeutic or inflammatory stress [37]. Within this context, the treatment-driven inflammatory cues amplify these effects, reinforcing checkpoint signaling while simultaneously suppressing cytotoxic T_c_ function. The analysis complements this by showing that tumors with more primitive transcriptional programs, consistent with reduced RD3 activity, are embedded within microenvironments enriched for suppressive myeloid cells that further potentiate immune paralysis. Importantly, RD3 appears to act upstream of these cascades, linking tumor cell differentiation status to immune visibility by modulating cytokine output, antigen presentation capacity, and responsiveness to inflammatory stimuli. Collectively, these findings align with the earlier discussion by positioning immune checkpoint signaling as part of a broader, differentiation-dependent regulatory network in NB. They suggest that durable immunotherapeutic responses will likely require strategies that not only block checkpoint pathways but also restore or mimic RD3-driven differentiation programs, thereby reversing tumor cell plasticity and dismantling the layered immunosuppressive architecture characteristic of HR disease. Importantly, checkpoint signaling is shown to extend beyond direct lymphocyte inhibition, reprogramming myeloid cells toward phenotypes that further dampen antigen presentation, secrete inhibitory mediators, and sustain chronic immune paralysis [15,37]. Collectively, these findings by illustrating that immune checkpoint pathways in NB function as a coordinated, plastic network rather than isolated signaling nodes. They underscore that effective immunotherapeutic strategies will require rational combination approaches that simultaneously disrupt checkpoint signaling, reverse myeloid-driven suppression, and counteract therapy-induced immune adaptation, thereby restoring durable antitumor immunity in this historically immunoresistant pediatric malignancy.

#### 3.2.2. TDEs and Vesicle-Mediated Immunosuppression

TDEs, particularly exosomes, are now recognized as highly efficient mediators of immunosuppression in NB, functioning as long-range conveyors of tumor-programmed signals within both the local TME and the systemic immune compartment. NB-derived exosomes carry a diverse molecular cargo, including immune checkpoint ligands, suppressive cytokines, metabolic enzymes, microRNAs, and lipids, that collectively blunt antitumor immune responses [29,43]. Functionally, these vesicles inhibit T_c_ receptor (TCR) signaling and suppress NK cell cytotoxicity, thereby directly impairing the effector arms of adaptive and innate immunity. In parallel, exosomal signaling interferes with DCs maturation and antigen-presenting capacity, limiting efficient T_c_ priming and skewing immune responses toward tolerance rather than activation. NB-derived exosomes further promote the expansion and functional polarization of immunosuppressive myeloid populations, including MDSCs and TAMs, reprogramming macrophages toward tumor-supportive, anti-inflammatory phenotypes that reinforce immune escape [44,45]. Importantly, the immunomodulatory effects of exosomes are not confined to the tumor site but extend systemically, contributing to peripheral immune dysfunction and reduced responsiveness to immunotherapeutic interventions such as checkpoint blockade or antibody-based therapies [29]. Collectively, these findings establish NB-derived exosomes as central architects of immune suppression, positioning them as valuable biomarkers of tumor-induced immune remodeling and as compelling therapeutic targets for disrupting vesicle-mediated communication and restoring effective antitumor immunity [29].

#### 3.2.3. Metabolic Suppression and Hypoxic Microenvironments

The NB TME is characterized by profound metabolic stress driven by regional hypoxia, nutrient deprivation, and oxidative imbalance, which collectively impose a strong suppressive pressure on antitumor immune responses [46]. Hypoxic niches arise as a consequence of rapid tumor growth and inadequate vascularization, leading to stabilization of hypoxia-inducible factor (HIF) signaling pathways that reprogram both tumor and stromal cells [47]. Persistent HIF activation promotes angiogenesis, EMT-like behavior, and metastatic dissemination, hallmarks that are well described in NB biology, while simultaneously dampening immune surveillance. From an immunological perspective, hypoxia directly impairs T_c_ and NK cell function by reducing cytotoxic granule release, cytokine production, and proliferative capacity [48]. These defects are compounded by metabolic exhaustion, as tumor cells outcompete infiltrating lymphocytes for glucose, amino acids, and oxygen, depriving effector cells of substrates required for activation and clonal expansion. In parallel, enhanced glycolytic flux within tumor cells drives accumulation of lactate and other acidic metabolic byproducts in the extracellular space, creating a hostile biochemical milieu that further suppresses lymphocyte signaling, motility, and survival. Hypoxia-driven metabolic rewiring also induces the expression of immunosuppressive genes, including those involved in adenosine production, redox control, and lipid metabolism, aligning with broader cancer-associated metabolic programs [46,48]. Collectively, these metabolic and hypoxic constraints act as an early and largely silent layer of immune evasion, weakening effector cell function and immune priming before classical immune checkpoint pathways are even engaged. As such, metabolic suppression represents a foundational barrier to effective antitumor immunity in NB and a critical consideration for the design of combinatorial immunotherapeutic strategies.

#### 3.2.4. CT-Induced Immune Remodeling

While CT remains a cornerstone of NB treatment due to its direct cytotoxic effects on rapidly dividing tumor cells, accumulating evidence indicates that it paradoxically reshapes the TME in ways that promote immune dysfunction and long-term immune suppression [49,50]. High-resolution scRNA-seq analyses of NB tumors obtained before and after CT have revealed dynamic but often detrimental alterations in immune composition and function. Although transient immune activation can occur during early treatment phases, post-therapy landscapes are frequently dominated by exhausted T_C_s, metabolically impaired NK cells, and expanded immunosuppressive myeloid populations. These changes reflect both direct cytotoxic injury to immune effectors and indirect effects mediated through therapy-induced inflammation, tissue damage, and altered cytokine gradients. CT has been shown to promote upregulation of inhibitory pathways, including immune checkpoint ligands, stress-response signaling, and suppressive cytokines, which together blunt CTL persistence and antitumor efficacy [49,51]. Concurrently, treatment-induced remodeling of stromal and myeloid compartments reinforces immune tolerance by favoring antigen-poor presentation states and tumor-supportive macrophage phenotypes. Importantly, these post-CT immune states are not merely passive consequences of treatment but actively constrain the effectiveness of subsequent immunotherapeutic interventions, including immune checkpoint blockade, antibody-based therapies, and cellular IMTs [50]. Rather than creating a uniformly permissive immune environment, CT often leaves behind a deeply dysfunctional and suppressive immune architecture that tumors can exploit to evade immune surveillance.

Our recent work elucidates how CT profoundly remodels the immune landscape of NB through mechanisms tightly linked to tumor-intrinsic differentiation programs governed by RD3 [15,37]. Cytotoxic therapy simultaneously perturbs the immune microenvironment by inducing marked shifts in T and NK cell functionality, characterized by transient activation followed by sustained dysfunction and exhaustion. Importantly, these immune changes are not uniform but strongly influenced by RD3 status within tumor cells. RD3, a differentiation-associated tumor suppressor, plays a central role in maintaining neuronal identity and restraining inflammatory and stress-response pathways. CT-induced downregulation or functional disruption of RD3 drives tumor dedifferentiation, which in turn amplifies inflammatory signaling, NFκB activation, and the expression of immunosuppressive mediators. Within this RD3-low, post-CT state, tumor cells secrete cytokines and metabolites that further impair CTL persistence and promote immune exhaustion. Concurrently, CT reshapes myeloid compartments, expanding suppressive macrophage and myeloid-derived suppressor populations that reinforce T_c_ inhibition and limit antigen presentation. Our findings indicate that RD3 loss acts as a molecular switch linking chemotherapeutic stress to immune escape, effectively converting treatment-induced inflammation into a tolerogenic, tumor-protective response [15,37]. As a result, the post-therapy TME becomes less responsive to subsequent immunotherapeutic interventions, including checkpoint blockade and antibody-based strategies. Collectively, these data reposition CT as a potent immunomodulator in NB rather than a purely cytotoxic modality. They further highlight RD3 as a critical molecular determinant of how CT shapes immune remodeling, underscoring the need for therapeutic strategies that preserve or restore RD3-associated differentiation programs to prevent immune collapse and enhance IMT efficacy following cytotoxic treatment [15,37].

#### 3.2.5. Immune Cell Trafficking

Immune cell trafficking is a critical determinant of anti-tumor immunity in NB and is frequently subverted to promote immune evasion. The recruitment and spatial distribution of immune cells within the NB TME are tightly regulated by chemokine-chemokine receptor networks, which can either support CTL infiltration or favor immunosuppressive cell accumulation. In HR-NB, tumor cells often exhibit epigenetic suppression of interferon-inducible chemokines such as CXCL9 and CXCL10, key mediators of CD8^+^ T_c_ trafficking, resulting in a “cold” tumor phenotype with poor lymphocyte infiltration [52]. Concurrently, NB cells and stromal components produce chemokines (e.g., CXCL1/2, CXCL12, CCL2) that preferentially recruit MDSCs, TAMs, and TREGs, thereby establishing an immunosuppressive niche that inhibits effective anti-tumor responses [53]. The CXCR4/CXCL12 axis further contributes to both tumor cell dissemination and immune modulation, shaping leukocyte trafficking patterns that dampen immune surveillance [54]. Collectively, this imbalance between deficient effector T_c_ recruitment and enhanced trafficking of suppressive immune populations represents a key mechanism of immune escape in NB, highlighting chemokine signaling pathways as promising therapeutic targets to restore immune infiltration and improve immunotherapy efficacy.

Overall, the NB TME is an intricate, highly coordinated network of immune and stromal cells that collectively shape tumor progression, immune evasion, and therapeutic resistance. The immune landscape is dominated by dysfunctional NK and T_C_s cells, regulatory myeloid populations, inhibitory checkpoint interactions, and potent exosome-mediated suppression. Metabolic constraints and CT-induced reprogramming further exacerbate the immunosuppressive milieu. Together, these factors form a formidable barrier to IMT success. However, advances in single-cell profiling, immune checkpoint biology, and exosome research are revealing new vulnerabilities within the NB TME. Restoring RD3, targeting TIGIT, restoring NK cell cytotoxicity, blocking exosome pathways, and reversing myeloid cell suppression represent promising future directions. A deeper understanding of the TME will refine immunotherapeutic strategies and ultimately improve outcomes for children with HRNB.

## 4. Mechanisms of Tumor Immune Evasion in NB

**Figure 3:**
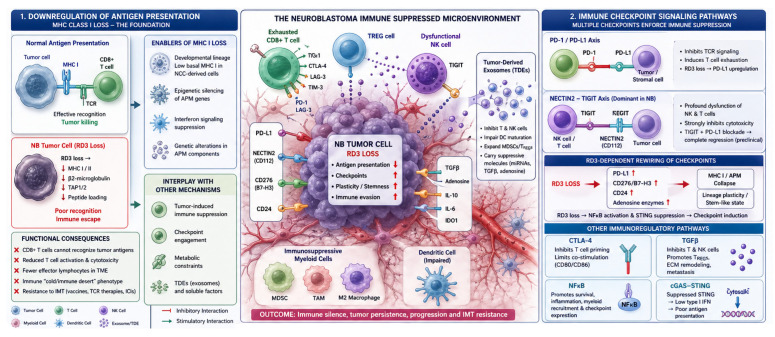
***Mechanisms of Tumor Immune Evasion in NB.*** NB exhibits coordinated yet convergent mechanisms of immune evasion, including: the downregulation of antigen presentation; T_c_ exhaustion; TREGs increase; dysfunctional NK cells; TDEs; increase in immunosuppressive myeloid cells; activation of immune checkpoint (PD1-PD-L1) pathways inhibiting TCR signaling and inducing T_c_ exhaustion; Nectin2-TIGIT signaling flow prompting NK and T_c_ dysfunction; therapy-pressure driven RD3-PD-L1, RD3-CD276/B7-H3, RD3-CD24, RD3-Adenosinergic, RD3-NFkB, RD3-STING axes rewiring of checkpoints; and other immunoregulatory pathways including CTLA 4, TGF β etc., that plays critical role in shaping both local and systemic immune dysfunction and contribute to IMT resistance.

NB exhibits a sophisticated capacity for immune evasion, enabling tumor cells to persist, progress, and resist therapeutic interventions despite the presence of innate and adaptive immune surveillance. A defining feature of NB is its profoundly immunosuppressive TME, which undermines the cytotoxic potential of NK cells, T_C_, and other immune effectors [8,55]. The gene surrogate studies and single-cell profiling from others has revealed extensive infiltration by NK, T, and myeloid cell populations, yet these cells are often rendered dysfunctional, displaying reduced cytotoxicity, exhaustion phenotypes, and impaired cytokine responses. This intrinsic suppression reflects the tumor’s ability to remodel the immune landscape and establish a permissive environment for tumor survival. Central to NB immune evasion is the engagement of inhibitory immune checkpoint pathways [12,56]. Recent analyses identify the RD3–immune axis as a dominant suppressive mechanism [15,37], and more broadly, NB tumors exploit canonical cancer immune-evasion pathways, including NECTIN2, PD-1/PD-L1, CTLA-4, TGF-β, and NFκB signaling, to curtail immune activation and promote tolerance, consistent with strategies seen across multiple malignancies [12]. Another major contributor to immune evasion is TDEs. In NB, TDEs suppress T and NK cell activity, inhibit DC maturation, and expand immunosuppressive myeloid populations, collectively reinforcing a hostile environment for effective immune responses. These vesicles play a critical role in shaping both local and systemic immune dysfunction, contributing to IMT resistance [29,44]. Together, these mechanisms, checkpoint engagement, cellular suppression, metabolic constraints, and exosome-mediated modulation, underscore why NB remains one of the most formidable pediatric cancers to treat with IMT (Figure 3). Understanding these immune-evasion pathways is essential for designing next-generation IMT strategies capable of overcoming NB’s entrenched resistance.

### 4.1. Downregulation of Antigen Presentation Machinery

#### 4.1.1. MHC Class I Downregulation in NB as a Foundational Immune Evasion Strategy

A central hallmark of tumor immune evasion is the downregulation of MHC-I molecules, a key component required for CTL recognition and elimination of malignant cells [57]. Under normal physiological conditions, MHC-I molecules present intracellular peptide fragments on the surface of cells, enabling CD8^+^ T lymphocytes to detect and destroy abnormal or virally infected cells. In cancer, loss or reduction of MHC-I surface expression is a well-documented mechanism by which tumor cells avoid CTL surveillance [58]. Tumors may achieve this by downregulating MHC-I heavy chains, suppressing β2-microglobulin expression, disrupting antigen processing machinery (APM) components such as TAP1/2, and/or repressing interferon-signaling pathways required for MHC-I induction [57]. These strategies collectively impair CTL recognition, allowing tumor cells to proliferate unchecked. Such immune evasion adaptations “allow tumors to evade immune surveillance and destruction,” underscoring the importance of altered antigen presentation in cancer progression [14,59]. This mechanism is particularly relevant in NB, where diminished antigen presentation is widely recognized as a core biological feature that contributes significantly to immune escape and therapeutic resistance.

#### 4.1.2. MHC Class I Downregulation in NB

Although NB-specific antigen-presentation pathways are still being deeply characterized, NB is one of the most prominent examples of intrinsic MHC-I downregulation. NB originates from embryonic NCC-derived sympathetic precursors, a developmental lineage that naturally expresses low basal levels of MHC-I, even in normal physiological states [60]. Tumor cells exploit this developmental characteristic, maintaining or further reducing MHC-I expression, thereby reinforcing resistance to CD8^+^ T_c_-mediated killing. Consequently, NB tumors often display an “immunologically cold” phenotype, characterized by poor T_c_ infiltration, low neoantigen burden, minimal interferon signaling, and a TME unfavorable for adaptive immune activation [21,60].

#### 4.1.3. Functional Consequences for T_C_-Mediated Tumor Recognition

Given the essential role of MHC-I in orchestrating CTL activity, its downregulation in NB has profound implications for immune failure. With insufficient MHC-I on the tumor surface: CD8^+^ T_c_ cannot efficiently recognize tumor antigens; antitumor T_c_ activation is markedly reduced; adoptively transferred or vaccine-induced T_c_ fail to exert meaningful cytotoxicity; and the TME accumulates fewer effector lymphocytes, weakening adaptive immunity [57,61,62]. Mechanistically, our recent investigations identified that the RD3–MHC axis is a central determinant of NB immunogenicity [15,37]. RD3 preserves antigen presentation by sustaining MHC-I expression and the associated peptide-processing machinery, enabling effective cytotoxic T_c_ recognition. When RD3 is lost, NB cells undergo a coordinated shutdown of MHC-I/II, β2-microglobulin, TAP transporters, and peptide-loading components [37]. This suppression is reinforced by RD3-dependent lineage destabilization, which drives cells toward a plastic, stem-like, immune-silent state. The resulting collapse of antigen presentation reduces T_c_ infiltration and promotes immune evasion. Collectively, these findings position RD3 as a key guardian of the MHC axis in NB [15,37]. These consequences align with the broader cancer immunology framework that tumors with impaired MHC-I expression create an “immune desert” or “immune-excluded” phenotype characterized by poor T_c_ infiltration and inadequate antigen presentation. Because of this, many IMTs that rely on efficient antigen recognition (neoantigen vaccines, TCR-based therapies, and ICIs) struggle to produce durable responses in NB.

#### 4.1.4. Interplay Between MHC-I Loss and Other Immune Evasion Mechanisms

MHC-I downregulation rarely occurs in isolation. Instead, it synergizes with multiple other immune evasion processes. These include:

Tumor-Induced Immune Suppression—NB tumors create highly immunosuppressive TME through cytokines, metabolic remodeling, and regulatory immune cells. Such suppression dampens interferon-γ signaling, which would otherwise promote MHC-I upregulation. This feedback loop strengthens the tumor’s ability to resist CTL killing [14,63].
Immune Checkpoint Regulation—NB employs multiple checkpoint ligands such as PD-L1 and NECTIN2 to inhibit T and NK cell activity. Tumors with reduced antigenicity tend to rely more heavily on checkpoint pathways [12,14].
Genetic and Epigenetic Modifiers—Cancer cells may epigenetically silence APM genes or mutation-inactivate components of the MHC-I presentation pathway and serves as the core driver of immune escape [64].

Together, these mechanisms create a multifaceted immune shield, with MHC-I downregulation serving as a central pillar in a broader immune-evasive strategy.

#### 4.1.5. Therapeutic Implications

Understanding MHC-I downregulation has significant implications for designing more effective IMT for NB. Some of the key strategies include:

Enhancing MHC-I Expression—Strategies to upregulate MHC-I, such as interferon-based therapies, epigenetic modulators, or agents that boost antigen processing, may sensitize NB tumors to T_C_-based therapies [59]. The RD3–MHC axis offers a compelling therapeutic entry point in NB. Because RD3 loss collapses MHC-I antigen presentation and creates an immune-silent state, restoring this axis could resensitize tumors to T_C_-based therapies. Strategies that establish RD3 function, stabilize its downstream transcriptional programs may reverse immune escape [15,37]. This creates opportunities to combine RD3-restorative approaches with checkpoint blockade, adoptive T_c_ therapies, or tumor vaccines. Targeting the plastic, RD3-deficient state itself, through differentiation agents or inhibitors of stemness pathways, may further re-enable antigen presentation.
Leveraging NK Cell-Based Therapies—Because NK cells are programmed to kill cells with low MHC-I (“missing-self recognition”), therapies that enhance NK cell activity may compensate for reduced CTL surveillance. This rationale is strongly supported by NB studies revealing compromised NK function in HR disease and highlighting checkpoint mechanisms such as TIGIT that restrain NK cytotoxicity [12,36].
Combination IMTs—Studies have stressed the importance of personalized combination approaches to overcome immune evasion mechanisms, integrating multi-omics data to rationally design therapies that target multiple escape pathways simultaneously. For NB, this may include combining NK-activating agents, checkpoint inhibitors, and antigen-presentation modulators [14].

Overall, downregulation of MHC class I is a foundational mechanism by which NB evades immune detection. By limiting CTL engagement, shaping an immune-cold microenvironment, and synergizing with other suppressive pathways, diminished antigen presentation significantly challenges the efficacy of current IMT. Addressing antigen presentation deficits is essential for developing next generation, multi-modal IMT approaches capable of overcoming TIME and improving outcomes for patients with NB.

### 4.2. Immune Checkpoint Signaling Pathways in NB

Immune checkpoint signaling pathways play a central role in shaping the TME and modulating the activity of CTLs in NB. These pathways enable tumors to inhibit immune activation, suppress cytotoxic effector cell function, and evade immune surveillance. While some checkpoints, such as PD-1/PD-L1, represent canonical mechanisms shared across multiple cancers, others (e.g., NECTIN2–TIGIT axis) appear to be uniquely dominant in NB, reflecting disease-specific immunobiology [12]. In addition, a constellation of auxiliary immunoregulatory pathways, including CTLA-4, TGF-β, NFκB, and cGAS–STING, further reinforces immune suppression and supports tumor progression, consistent with broader principles of TIME [14]. Here we discuss some of these immune checkpoint pathways, their roles in NB immune evasion, and their implications for IMT.

#### 4.2.1. PD-1/PD-L1 Axis in NB

The PD-1 receptor and its ligand PD-L1 constitute one of the most widely studied immune checkpoint pathways across human malignancies. Although PD-1/PD-L1 blockade has revolutionized treatment for adult cancers, its role in pediatric solid tumors, particularly for NB, is more complex [41,65]. Nonetheless, the underlying biology of this pathway is that the tumors exploit PD-1/PD-L1 signaling to silence T_c_ responses, promote T_c_ exhaustion, and establish immune tolerance. In NB, PD-L1 expression is often low at baseline but can be induced under inflammatory or therapeutic pressure, particularly in response to interferon signaling or CT exposure [65,66]. To that end, in NB activated T_c_ secrete IFN-γ, which can drive PD-L1 expression on tumor and stromal cells, creating a negative feedback loop; Post-CT tumors show increased PD-1^+^ exhausted T_c_ and increased PD-L1 expression in the TME, mirroring the single-cell-Seq NB study observations showing fluctuating T_C_-cell functionality across treatment stages [12]. Mechanistically, the RD3–PD-L1 axis represents a critical immune-evasion pathway in NB. Loss of RD3 drives upregulation of PD-L1, creating a dual barrier to T_c_-mediated immunity. RD3-deficient cells adopt a plastic, stem-like state that reinforces immune silence, and PD-L1 induction emerges as part of this broader reprogramming. Elevated PD-L1 further inhibits cytotoxic T_c_ activity, enabling tumor persistence despite immune pressure [15,37]. Together, these changes position RD3 loss as a key upstream event that licenses PD-L1-mediated immune escape and shapes an immunosuppressive TME in NB. Functionally, the PD-1/PD-L1 axis contributes to NB immune suppression by inhibiting TCR signaling; reducing T_c_ proliferation and cytokine production; promoting T_c_ exhaustion, impairing the ability to mount durable antitumor responses; and enabling tumor escape even in the presence of tumor-infiltrating lymphocytes [41]. Therapeutic strategies including checkpoint inhibitors targeting PD-1/PD-L1 have shown limited single-agent activity in NB, likely due to low baseline PD-L1 expression; low tumor mutational burden; poor T_c_ infiltration; and co-dominance of another checkpoint (RD3, TIGIT, etc.) [12,65]. To that end, the RD3–PD-L1 axis presents a promising therapeutic vulnerability in NB. RD3 loss not only suppresses antigen presentation but also induces PD-L1, creating a potent immune-evasion program. Targeting this axis could restore T_c_ activity and enhance responsiveness to IMT. Approaches that re-establish RD3 function, inhibit the plastic, stem-like state it enables, downregulate PD-L1, may reverse immune silence [15,37]. Combining RD3-restorative strategies with PD-1/PD-L1 blockade, adoptive T_c_ therapies, or differentiation agents could synergistically overcome immune resistance. Likewise, given strong synergy observed between TIGIT blockade and PD-L1 inhibition in preclinical NB models, combinatorial strategies remain highly promising [12].

#### 4.2.2. NECTIN2–TIGIT Axis in NB

The NECTIN2–TIGIT axis represents a pathway that is uniquely dominant in NB. Single-cell RNA-seq analysis in NB tumors revealed the NECTIN2–TIGIT interaction as the crucial immune checkpoint regulating both NK-cell and T_c_ dysfunction in NB. The TIGIT receptor is highly expressed on CTLs, TREGs and NKs. In NB, TIGIT^+^ NK and T_c_ display profound dysfunction, reduced cytotoxic signatures and features of exhaustion even at early treatment stages [12,67]. TIGIT expression fluctuates throughout CT, reflecting dynamic immune remodeling, a critical insight that underscores the importance of timing in IMT design. NECTIN2 (CD112) is abundantly expressed on NB cells and stromal components. Its interaction with TIGIT exerts a strong inhibitory effect on NK-cell cytotoxicity, T_C_ activation, and tumor killing both in vitro and in vivo [68]. This pathway’s dominance in NB demonstrated that the NECTIN2–TIGIT axis surpasses PD-1/PD-L1 in functional impact in the NB TME. TIGIT Blockade in preclinical NB models show that, TIGIT blockade alone improves immune activation, TIGIT + PD-L1 dual blockade induces complete tumor regression, and TIGIT blockade boosts NK-cell-mediated killing, especially important because NB often downregulates MHC class I and shifts tumor control toward NK-cell surveillance [12]. These findings highlight the TIGIT axis as a high-value therapeutic target, surpassing traditional checkpoint pathways in relevance to NB.

#### 4.2.3. RD3-Dependent Rewiring of Immune Checkpoint Signaling in NB

RD3 has emerged as a master upstream regulator of immune checkpoint signaling in NB, defining whether tumors remain visible or invisible to the immune system. Across diverse experimental systems, including engineered RD3 knockouts, silencing, inducible RD3-rescue lines, and patient-derived NB models, loss of RD3 consistently triggers a profound rewiring of the tumor–immune interface [15,37]. RD3 maintains a transcriptional state that supports antigen presentation and restrains immune-suppressive programs. When RD3 is lost, NB cells undergo a profound shift toward immune silence, activating a coordinated network of checkpoint molecules that blunt cytotoxic T_c_ activity. RD3-deficient cells activate a multilayered immune-suppressive program characterized by robust induction of PD-L1, CD276/B7-H3, CD24, and adenosine-generating enzymes, creating a potent inhibitory shield against cytotoxic T_c_ activity. This checkpoint upregulation occurs in parallel with a collapse of MHC-I antigen presentation, including suppression of β2-microglobulin, TAP transporters, and peptide-loading machinery, ensuring that even activated T_C_ cannot effectively recognize tumor cells. This dual mechanism, checkpoint upregulation coupled with antigen-presentation collapse, positions RD3 as a master gatekeeper of tumor–immune interactions [15,37]. Importantly, RD3-dependent checkpoint activation is tightly linked to lineage plasticity. As cells transition into a stem-like, therapy-resistant state, immune checkpoints become embedded within the reprogrammed transcriptional landscape, making immune escape a stable feature rather than a transient adaptation. This plasticity-linked immune silence is not merely correlative: inducible re-expression of RD3 reverses checkpoint activation, restores antigen presentation, and re-sensitizes tumor cells to immune pressure. RD3-deficient tumors grow more aggressively, exhibit markedly reduced T-cell infiltration, and display high PD-L1 and B7-H3 expression. Analyses of HR patient tumors further confirm that low RD3 expression aligns with immune-cold phenotypes and poor clinical outcomes [15,37]. These insights highlight the RD3 axis as a therapeutic fulcrum: restoring RD3 function, targeting plasticity programs, or directly inhibiting RD3-induced checkpoints may reawaken anti-tumor immunity.

**Figure 4:**
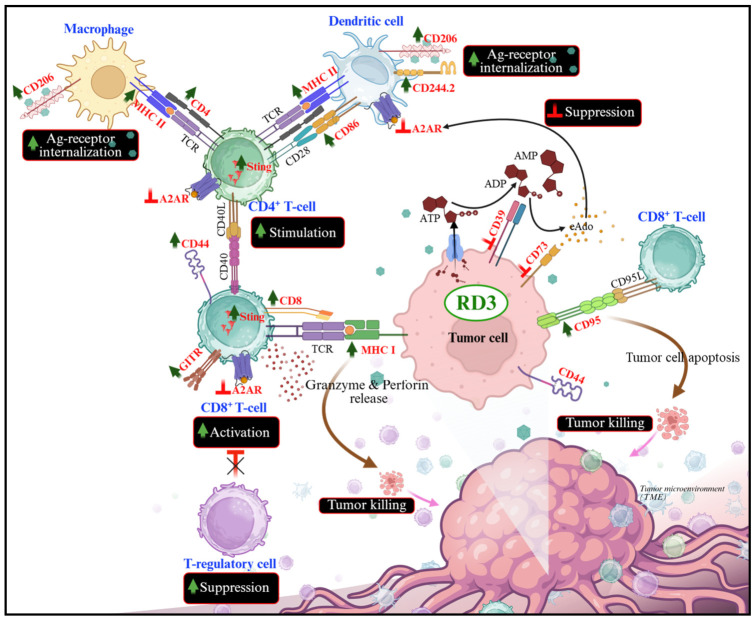
***RD3, the master regulator of TIME in NB.*** Therapy pressure steered RD3-loss coordinates NB cell immune escape by synchronously reshaping antigen presentation (CD206, MHCI, MHCII, B2M), adenosine metabolism (CD39, CD73, A2AR), innate-adaptive crosstalk (CD244.2, CD206), CTL fitness (GITR, STING, CD8, CD44), and apoptotic susceptibility (CD95). This coordinated remodeling explains why RD3-negative NB exhibits a “immune cold” immune phenotype.

Mechanistically, RD3-loss orchestrates immune escape by synchronously reshaping antigen presentation, adenosine metabolism, innate-adaptive crosstalk, CTL fitness, and apoptotic susceptibility. This coordinated remodeling explains why RD3-negative NB exhibits a “cold but deceptive” immune phenotype, one that contains immune cells but neutralizes their function (Figure 4).

Axis I: RD3-loss Adenosine-A2AR Signaling Rewires Metabolic Immune Suppression: RD3-controlled purinergic signaling is centered on ectonucleotidase modulation (CD39/CD73) and extracellular adenosine (eAdo) flux. Tumor-associated RD3 loss alters ATP processing, shifting the balance from immunostimulatory toward immunosuppressive adenosine accumulation. Elevated adenosine engages A2A receptors (A2AR) on CD4^+^, CD8^+^ T_c_, macrophages, etc. This axis imposes a metabolic checkpoint that dampens TCR signaling, suppresses STING activation, and reduces granzyme/perforin release despite preserved antigen recognition. Crucially, RD3-loss driven adenosine signaling does not eliminate immune cells; instead, it locks them into a hyporesponsive state, explaining why infiltrating T_c_ fails to translate presence into tumor control. The downregulation of A2AR signaling shown upon RD3 presence thus represents a metabolic “unlocking” of effector competence rather than classical immune recruitment [37].
Axis II: RD3-loss and Antigen Presentation Decoupling Recognition from Response: RD3 preserved or enhanced MHC I and MHC II expression on tumor cells and antigen-presenting cells (APCs), yet downstream suppression persists. This paradox suggests RD3-loss does not primarily drive immune evasion through antigen loss but through functional disconnection of antigen presentation from co-stimulation. In DCs and macrophages, RD3 reprogramming increases antigen receptor internalization while skewing surface marker expression (CD206, CD244.2). These features resemble tolerogenic or exhausted APC states, cells that efficiently ingest antigen but fail to license productive T_c_ activation. Despite intact TCR-MHC engagement, insufficient CD28-CD86 signaling and dominant suppressive cues blunt adaptive priming. Thus, RD3 loss enforces a “presentation-without-activation” phenotype, enabling tumor persistence under immune surveillance while avoiding overt immune elimination pressures [37].
Axis III: RD3–loss Innate Sensing Crosstalk–STING Dampening Without Inflammation Loss: STING appears centrally positioned in both CD4^+^ and CD8^+^ T_c_, emphasizing innate immune signaling as a convergence point. RD3-loss suppresses effective STING pathway activation. This selective attenuation is particularly consequential: STING is required not only for type I interferon production but also for sustaining CTL persistence, antigen spreading, and dendritic cell licensing. RD3 loss’s ability to mute STING signaling without eliminating immune infiltration creates a silent immune landscape, one where signaling thresholds are never crossed. Importantly, restoration of STING signaling upon RD3 reinforcement emerges as a mechanistic explanation for the observed increase in immune stimulation and tumor killing illustrated downstream. This positions RD3 as a molecular switch, regulating inflammatory amplitude rather than immune cell numbers [37].
Axis IV: RD3-loss Skews CD4^+^ T_c_ Fate Toward Suppression and Help Attrition: CD4^+^ T_c_ acts as a pivotal node of RD3-loss mediated control. RD3 deficiency reduces CD44-driven activation while favoring Tregs expansion and suppressive dominance. This shift deprives CD8^+^ T_C_ of essential helper signals required for sustained cytotoxicity, memory formation, and resistance to exhaustion. This mechanism is particularly relevant in NB, where CD4^+^ T_c_ often infiltrate tumors but fail to provide durable support. RD3-loss driven skewing explains the coexistence of immune infiltration with functional paralysis and highlights why IMTs alone have limited benefit unless helper compartment reprogramming is addressed [15].
Axis V: RD3-loss Impairs Cytotoxic Execution Despite Preserved CD8^+^ Engagement: On the cytotoxic front, RD3 loss does not prevent CD8^+^ T_c_ from forming immune synapses with NB cells. However, granzyme and perforin release are functionally suppressed, while death receptor signaling (CD95/CD95L) is rendered ineffective. This decoupling of recognition and killing results in abortive immune synapses, contacts that fail to trigger apoptosis despite proximity. RD3-loss thereby converts CD8^+^ T_c_ into passive witnesses rather than executioners, further reinforcing immune escape without requiring antigen loss or checkpoint upregulation alone [15].
Axis VI: RD3-loss Shapes Myeloid Plasticity Toward Immune Dampening: Macrophages and dendritic cells exhibit enhanced CD206 expression, indicative of alternatively polarized or tolerogenic states. RD3-loss influences myeloid plasticity while suppressing inflammatory instruction. This results in efficient clearance of immunogenic debris without propagation of danger signals, blunting epitope spreading and adaptive amplification. This axis underscores RD3-loss as a myeloid conditioning factor, capable of instructing innate cells to serve tumor persistence rather than immune defense [15,37].

Collectively, these axes converge on a single unifying principle: RD3-loss does not block immunity, it rewires it. Instead of inducing immune ignorance, RD3-loss enforces immune misdirection, tolerance, and inefficiency across cellular, metabolic, and signaling layers. By synchronizing adenosine metabolism, antigen presentation fidelity, STING signaling thresholds, helper T-cell licensing, and cytotoxic execution, RD3-loss constructs a TME that is immunologically populated yet functionally inert. This explains resistance to checkpoint blockade, limited durability of adoptive cell therapies, and the deceptive appearance of immune engagement in RD3-driven NB.

#### 4.2.4. Other Immune Checkpoints Relevant to NB

While PD-1/PD-L1 pathways dominate NB immune regulation, several other checkpoint pathways also contribute significantly to the immunosuppressive NB microenvironment. These include CTLA-4, TGF-β, NF-κB, and cGAS–STING.

##### CTLA-4 Signaling

CTLA-4 is an inhibitory receptor expressed on T_c_ that competes with CD28 for binding to costimulatory ligands (CD80/CD86). When CTLA-4 binds these ligands, T_c_ activation is suppressed. Although CTLA-4 blockade has transformed treatment in other cancers, its role in NB is more limited [21]. This is majorly attributed to that NB tumors have fewer activated T_c_ in the TME; show low levels of antigen presentation; and possess an immune-cold phenotype. Nonetheless, CTLA-4 signaling suppresses T_c_ priming and contributes to inadequate adaptive immune activation, and CTLA-4 as a pathway could be exploited broadly by NB to suppress immune activation [21,69].

##### TGF-β Signaling

TGF-β is one of the most potent immunosuppressive cytokines found in TME across cancers. Its roles include inhibiting T_c_ proliferation, suppressing NK-cell cytotoxicity, promoting TREG differentiation, and inducing ECM remodeling and metastasis. Studies have explicitly identified TGF-β as a major pathway contributing to TIME, modulating both innate and adaptive immunity [55,70]. In NB, elevated TGF-β levels correlate with impaired NK-cell activity, an especially critical issue given NB’s reliance on NK-cell surveillance due to low MHC-I expression [71].

##### NFκB Pathway

NFκB is a master transcription factor involved in inflammation, survival, and oncogenesis. Many cancers, including NB, exploit dysregulated NFκB signaling to promote tumor growth, suppress antigen-presentation pathways, and enhance resistance to cytotoxic immune mechanisms [72]. In NB, NFκB activation supports myeloid cell recruitment and inflammatory signaling that paradoxically promotes tumor progression rather than immune clearance [10]. Emerging evidence indicates that NFκB signaling operates in close crosstalk with the NRF2 pathway, a key regulator of antioxidant and anti-inflammatory responses. In NB, NRF2 activation contributes to tumor cell survival, metabolic adaptation, and therapy resistance, while also shaping a more immune-suppressive TME. NRF2-driven transcriptional programs enhance redox homeostasis and cytoprotective gene expression, which can blunt immunogenic cell death and reduce immune recognition. Moreover, NRF2 hyperactivation has been linked to diminished immune cell infiltration and impaired anti-tumor immunity, further reinforcing immune escape. Importantly, NRF2 can modulate NFκB activity through antioxidant response element (ARE)-dependent mechanisms, establishing a regulatory feedback loop that sustains tumor-promoting inflammation while limiting effective cytotoxic responses [73]. Our work identifies the RD3–NFκB axis as a critical upstream regulator of immune checkpoint signaling in NB [15,37]. We demonstrate that RD3 loss unleashes constitutive NFκB activation, shifting tumor cells into a pro-survival, immune-evasive state. This NFκB hyperactivation directly drives transcriptional upregulation of key immune checkpoints, including PD-L1, CD276/B7-H3, and CD24, establishing a multilayered inhibitory network that suppresses cytotoxic T_c_ engagement. Through integrated cell-line models, inducible RD3-rescue systems, and in vivo validation, we showed that restoring RD3 dampens NFκB signaling, reduces checkpoint expression, and re-sensitizes tumors to immune pressure. Transcriptomic profiling further reveals that NFκB-dependent checkpoint induction becomes embedded within the plastic, stem-like state created by RD3 deficiency [15,37]. Collectively, our findings position the RD3–NFκB axis as a mechanistic bridge linking lineage instability to immune suppression, defining a tractable target for reversing immune escape in NB.

##### cGAS–STING Pathway

The cGAS–STING pathway is an innate immune sensor of cytosolic DNA that typically activates type I interferon responses and promotes antitumor immunity. However, NB can suppress cGAS–STING signaling, disrupt interferon production, and prevent effective activation of immune cells. Impaired cGAS–STING signaling in NB, contributes to low interferon-γ signatures, reduced T_c_ recruitment, and inadequate antigen presentation [74,75]. Our studies reveal the RD3–STING axis as a pivotal regulator of immune-checkpoint signaling in NB. We show that RD3 is essential for maintaining proper STING activation and downstream type I interferon signaling, a pathway normally required for antigen presentation and immune surveillance [37]. RD3 loss disrupts STING trafficking and signaling competence, resulting in blunted interferon responses and diminished induction of immune-stimulatory genes. This impaired STING activity creates a permissive environment for immune-checkpoint upregulation, including heightened expression of PD-L1, CD276/B7-H3, and CD24. Using engineered RD3-deficient cell lines, inducible rescue systems, and in vivo tumor models, we demonstrate that restoring RD3 reactivates STING signaling, reduces checkpoint expression, and enhances T_c_ engagement [37]. Transcriptomic analyses further show that STING suppression becomes embedded within the plastic, stem-like state driven by RD3 loss. Collectively, these findings position the RD3–STING axis as a mechanistic link between disrupted innate sensing and immune-checkpoint-mediated escape in NB [37].

##### Membrane Signaling and ECM Dynamics

Within the broader framework of immune checkpoint signaling in NB, emerging evidence suggests that ECM remodeling and membrane-associated signaling pathways further modulate checkpoint function and immune suppression. Lipid raft–localized receptors such as LRP6 organize Wnt signaling within specialized membrane domains, enhancing signaling plasticity and potentially facilitating the clustering and stabilization of immune checkpoint ligands [76]. This spatial organization may reinforce dominant checkpoint axes, including PD-L1 and NECTIN2–TIGIT, by sustaining their surface expression and downstream signaling efficiency. Concurrently, studies in other neuronal tumors showed that ECM-modifying enzymes such as heparanase influence not only matrix architecture but also autophagy and apoptotic balance, thereby enabling tumor cells to adapt to immune and therapeutic stress [77]. Given that heparan sulfate interactions regulate receptor presentation and vesicle-mediated signaling, these processes may further amplify checkpoint-mediated immune evasion. Collectively, these findings suggest that membrane signaling architecture and ECM dynamics act in concert with canonical checkpoint pathways to stabilize immunosuppressive states and promote therapy-resistant tumor progression.

Overall, immune checkpoint pathways are central drivers of immune evasion in NB. TIME in NB is not the result of a single immunosuppressive pathway but instead reflects an interwoven network of checkpoint interactions. The multifaceted landscape of NB TME mirrors the broader complexity in TIME where some contribute to general adaptive immune resistance, while others interact for the NB-specific functionally consequential checkpoint mechanism, collectively protecting tumors from immune elimination. This implies that single agent checkpoint inhibitors are unlikely to be sufficient for NB, a disease-specific, mechanism focused (e.g., RD3–PD-L1, RD3–STING, RD3–NFκB, and Nectin2–TIGIT axis), personalized strategy is essential for developing next-generation IMT [21,78].

## 5. Metabolic Reprogramming and Immune Suppression in NB

**Figure 5:**
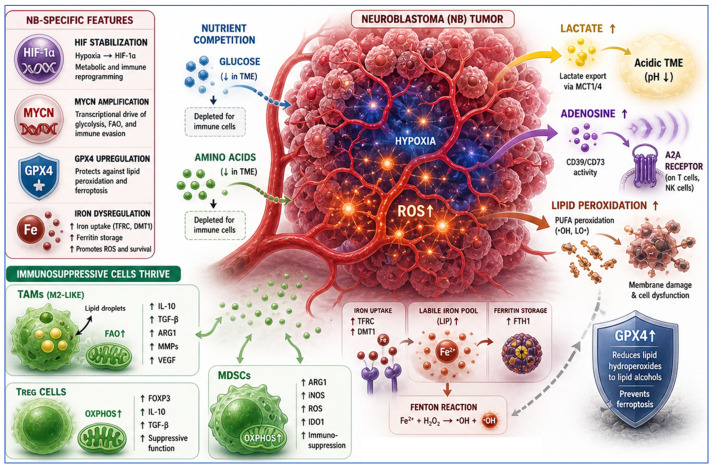
**Metabolic reprogramming and immune suppression in NB.** NB tumor cells (pink/red) under hypoxia (dark blue gradients) and MYCN amplification release toxic metabolites including lactate (yellow clouds causing acidosis), adenosine (purple waves activating A2A receptors), and ROS/lipid peroxidation products (orange particles), which suppress effector T_c_ (blue; exhausted mitochondria, reduced glycolysis/IFN-γ) and NK cells (teal; impaired cytotoxicity/granule release). Immunosuppressive cells including TAMs (green; FAO/lipid droplets), Tregs, and MDSCs thrive via metabolic adaptations like OXPHOS and ROS buffering. NB-specific features highlight HIF signaling, GPX4-mediated ferroptosis resistance (shield icon), and nutrient competition (depleted glucose/amino acids). Therapeutic targets (bottom) include GPX4 inhibition, ROS modulation, and hypoxia blockade to restore antitumor immunity.

Metabolic reprogramming is one of the most powerful and multifaceted mechanisms by which tumors suppress immune function [79]. In NB, the TME is shaped by metabolic constraints, including glucose deprivation, hypoxia, accumulation of lactate and ROS, altered lipid pathways, and ferroptosis-associated metabolic signatures, which collectively impair the function of key immune effector cells such as T_c_ and NK cells [80,81]. These metabolic disturbances not only promote tumor survival and treatment resistance but also create an environment in which antitumor immunity is profoundly compromised. To that end, immunometabolism studies emphasize that metabolic reprogramming is a core mechanism of tumor-driven immune suppression, affecting everything from nutrient availability to intracellular signaling pathways that dictate immune cell activation, differentiation, and persistence [79]. Similarly, NB-specific studies reveal that hypoxia, ROS accumulation, lipid metabolism rewiring, and ferroptosis-associated metabolic pathways are strongly enriched in HR-NB, contributing to aggressive behavior and immune dysfunction within the TME [81]. Here, we synthesize the mechanistic connections between tumor metabolism and immune suppression with NB-specific metabolic stressors (Figure 5).

### 5.1. Metabolic Reprogramming in Cancer and Its Impact on T_c_ and NK Cell Function

#### 5.1.1. Nutrient Competition and Metabolic Deprivation

One of the clearest metabolic modes of immune suppression is nutrient competition. Rapidly proliferating tumor cells consume glucose, amino acids (glutamine, arginine, tryptophan), and lipids at exceptionally high rates, leaving insufficient nutrients for infiltrating T_c_ and NK cells [14,82]. Metabolic reprogramming is a central mechanism promoting immune suppression, emphasizing that tumors create nutrient-poor microenvironments that “exhaust” immune effector cells and blunt cytotoxic responses [14]. Metabolic deprivation causes T_c_ and NK cells to shift from effector glycolytic programs to exhausted, hypoactive states. Effector lymphocytes rely heavily on glycolysis and amino acid metabolism to generate ATP and biosynthetic intermediates required for proliferation, cytokine production, and cytotoxic granule release. When tumors consume these substrates first, immune cells experience diminished proliferation, reduced cytotoxicity, lower cytokine secretion (e.g., IFN-γ), impaired memory formation, and loss of mitochondrial function. Effector T_c_, in particular, undergo “metabolic exhaustion,” a key barrier to antitumor immunity. NK cells, which depend on glucose flux and mitochondrial fitness for cytotoxicity, similarly fail in metabolically starved TMEs [83,84]. Thus, metabolic deprivation is a universal suppressor of lymphocyte activity, laying the foundation for metabolic immune evasion in NB.

#### 5.1.2. Toxic Metabolite Accumulation: Lactate, Adenosine, and Lipid Byproducts

Alongside competition for nutrients, tumor cells impose immune suppression by releasing toxic metabolites that accumulate in the TME. Cancer cells export: (i) lactate, creating acidic conditions that impair T_c_ and NK cell activity [85]; (ii) adenosine, which suppresses immune activation through A2A receptors [86]; and (iii) lipid peroxidation products, which damage immune cell membranes and signaling [87]. These metabolites as drivers of local immunosuppression, forming acidic, hypoxic niches enriched with ROS that accelerate immune exhaustion [83]. In these toxic niches: (i) T_c_ downregulate TCR signaling pathways [88], (ii) NK cells lose cytotoxic granule release capacity [89], and (iii) dendritic cells fail to mature properly [90]. These combined effects shape an immune landscape in which tumor cells are shielded from adaptive and innate responses.

#### 5.1.3. Preference of Immunosuppressive Cells for Alternative Metabolic Programs

While T and NK cells falter in nutrient-deprived conditions, many immunosuppressive cell populations thrive. Tregs, MDSCs, and TAMs exhibit a high degree of metabolic plasticity, enabling them to adapt by switching to pathways such as fatty acid oxidation (FAO); oxidative phosphorylation (OXPHOS); and lipid scavenging. These adaptations promote their survival and enhance local immune suppression [91,92]. This metabolic asymmetry, immunosuppressive cells adapting successfully while effector cells fail, not only depletes resources but selectively empowers the suppressive arms of the immune system.

### 5.2. Hypoxia, ROS, and Metabolic Stress in the NB TME

Hypoxia, oxidative stress, and metabolic reprogramming are defining features of the NB TME, particularly in HR-NB. Spatial transcriptomics and proteomic profiling of human tumors consistently reveal hypoxic niches embedded within densely proliferative tumor regions, where limited perfusion drives stabilization of HIF signaling and broad transcriptional rewiring [46,47]. These hypoxic zones generate intense ROS flux, promoting genomic instability, stress-adapted survival pathways, and selection of aggressive tumor subpopulations. Elevated ROS also intersects with oncogenic MYCN activity, amplifying metabolic pressure and reinforcing a pro-tumorigenic redox state [93]. Metabolic stress extends beyond oxygen deprivation. HR-NB exhibits profound alterations in lipid metabolism, including enhanced fatty-acid uptake, β-oxidation, and lipid droplet accumulation, features that support energy flexibility and resistance to therapy. These lipid-driven programs converge with ferroptosis-associated metabolic pathways, where dysregulated iron handling, lipid peroxidation, and antioxidant defenses shape tumor vulnerability or resilience depending on context [94]. Together, hypoxia, ROS, and metabolic remodeling create a TME optimized for tumor persistence, TIME, and therapeutic resistance. Understanding how these metabolic pressures sculpt NB biology provides a foundation for targeting metabolic stress pathways as a therapeutic strategy.

#### Hypoxia as a Fundamental Driver of NB Immune Suppression

Hypoxia is one of the most pervasive and influential metabolic pressures within the NB TME. Several studies identified hypoxia as a defining hallmark of tumors that shapes growth, metastasis, angiogenesis, TIME, and therapeutic resistance [46,47]. In NB, hypoxia arises from the tumor’s chaotic, poorly organized vasculature, which produces regions of low oxygen tension, restricted glucose and nutrient availability, accumulation of metabolic waste, and high lactate levels that acidify the local environment. These conditions stabilize HIF factors 1α and 2α, which activate broad transcriptional programs that promote angiogenesis, enhance metastatic potential, and enable tumor cells to survive under metabolic stress [47]. Crucially, hypoxia exerts profound immunosuppressive effects. HIF-driven signaling reduces immune-cell infiltration by altering chemokine gradients, suppresses antigen-presentation pathways, and promotes expression of immune-evasive molecules. Hypoxic stress also impairs T_c_ and NK-cell effector function, driving exhaustion phenotypes and weakening cytotoxic responses [48]. In NB specifically, hypoxia reinforces aggressive tumor behavior by promoting metabolic states associated with poor therapy response, lineage plasticity, and resistance to apoptosis [48]. Together, these findings position hypoxia as a central metabolic force that not only fuels NB progression but also establishes an immune-silent microenvironment that enables tumor persistence despite immune pressure.

### 5.3. ROS Accumulation and Oxidative Stress in NB

ROS accumulation is a defining metabolic hallmark of the NB TME, reflecting the intense metabolic and oxidative pressures that shape HR disease [95]. Elevated ROS in NB arises from enhanced mitochondrial respiration, oncogene-driven redox dysregulation, hypoxia–reoxygenation cycles, and lipid peroxidation associated with ferroptosis-linked pathways. Excessive ROS disrupts immune signaling, damages cellular macromolecules, and promotes tumor immune evasion through redox-mediated suppression of antitumor immunity [95]. In the immune compartment, ROS exerts broad inhibitory effects. High oxidative stress damages mitochondrial membranes in T_c_ and NK cells, impairs T-cell receptor signaling, and promotes apoptosis of effector lymphocytes. ROS also supports the expansion and suppressive activity of TAMs and MDSCs, further reinforcing an immunosuppressive microenvironment [10]. Spatial multi-omics analyses of HR-NB reveal pronounced ROS signaling and upregulation of glutathione metabolism, indicating that NB cells rely heavily on antioxidant defenses such as glutathione peroxidase 4 (GPX4) to prevent ferroptosis and maintain survival under oxidative stress [96]. This redox-adapted state not only fuels tumor persistence but also contributes to immune escape and therapy resistance, positioning ROS regulation as a critical metabolic axis in NB biology.

### 5.4. Ferroptosis-Associated Metabolic Pathways in HRNB

Ferroptosis-associated metabolic reprogramming has emerged as one of the most distinctive metabolic features of HR-NB. Recent spatial multi-omics studies reveal that NB tumors display strong enrichment of ferroptosis-linked signatures, including increased fatty-acid metabolism, elevated ROS signaling, and high expression of glutathione-dependent antioxidant pathways [80,97]. Central to this phenotype is the upregulation of GPX4, a key enzyme that detoxifies lipid peroxides and prevents the catastrophic membrane damage characteristic of ferroptotic cell death. Although NB cells are metabolically primed for ferroptosis due to their heightened lipid peroxidation potential, they compensate by activating GPX4-driven protective programs that maintain redox balance and support tumor survival. Experimental inhibition of GPX4 leads to rapid accumulation of lipid peroxides and a marked reduction in NB cell viability, confirming that ferroptosis resistance is an active, targetable metabolic adaptation. This ferroptosis-resistant state critically contributes to immune evasion [80,96]. ROS handling, lipid metabolism, and ferroptosis pathways are tightly intertwined with immune regulation, and NB cells that suppress ferroptosis often exhibit reduced immunogenicity and enhanced tolerance to oxidative stress within the tumor microenvironment [80,96]. To that end, ferroptosis resistance, largely mediated by upregulation of GPX4, limits lipid peroxidation–driven immunogenic cell death and the release of damage-associated molecular patterns (DAMPs), thereby reducing dendritic cell activation and downstream T-cell priming [76,98,99]. Furthermore, maintenance of redox homeostasis in ferroptosis-resistant cells supports an immunosuppressive microenvironment enriched in tolerogenic myeloid populations and impaired CTL function [98,100]. In contrast, induction of ferroptosis has been linked to enhanced antigen presentation and increased sensitivity to immune-mediated killing, highlighting its potential to modulate immune checkpoint responsiveness [76,98,99]. Collectively, these findings position ferroptosis as a dynamic immunometabolic regulator that intersects with antigen presentation, innate immune signaling, and checkpoint-dependent immune escape in NB.

### 5.5. Macrophage–Tumor Interactions and Metabolic Rewiring

HR-NB tumors contain macrophage-enriched niches that play a central role in shaping the metabolic and immunological landscape of the TME. Spatial proteomics consistently identifies dense infiltrates of TAMs exhibiting pro-angiogenic phenotypes, immunosuppressive signaling, and metabolic activation linked to lipid utilization and ROS-driven pathways [49]. These macrophage-dominated regions correlate strongly with poor patient survival, reflecting broader cancer-wide mechanisms in which TAMs undergo metabolic rewiring to sustain immune suppression and support tumor progression [49]. Metabolic crosstalk between NB cells and TAMs reinforces this suppressive ecosystem. Lipid-rich and oxidative conditions within the NB TME promote TAM polarization toward an immunoregulatory state, enhancing their ability to inhibit T_c_ activity, remodel extracellular matrices, and stimulate angiogenesis. Studies across cancer models emphasize that TAMs adapt their metabolism, particularly FAO and redox buffering, to maintain suppressive function under metabolic stress [101]. Longitudinal single-cell analyses in NB further reveal that macrophage populations expand following CT, accompanied by heightened inflammatory and metabolic activity. This post-treatment macrophage surge contributes to therapeutic resistance by reinforcing immune suppression and supporting tumor regrowth [49]. Together, these findings position macrophage–tumor metabolic interactions as a critical driver of NB aggressiveness and a promising target for therapeutic intervention.

Overall, metabolic reprogramming is a defining feature of NB biology and a powerful driver of TIME. Nutrient competition, toxic metabolite accumulation, and immunosuppressive metabolic adaptations undermine T and NK cell function. Hypoxia, ROS accumulation, ferroptosis-associated signaling, and macrophage-driven metabolic rewiring further shape a TME permissive for tumor progression and resistant to IMT. Insights from cancer-wide immunometabolism demonstrate that metabolic reprogramming is one of the most potent forms of TIME. Meanwhile, NB-specific multi-omic analyses provide compelling evidence that HR-NB tumors harbor distinct metabolic vulnerabilities, including ferroptosis pathways and ROS–lipid metabolism interactions. Together, these data underscore the need for metabolic-targeted IMT strategies, including GPX4 inhibition, ROS modulation, hypoxia-targeted therapy, metabolic checkpoint blockade, and nutrient-reprogramming approaches, to overcome immune suppression and improve outcomes for children with HR-NB.

## 6. Epigenetic Regulation of Immune Evasion

Epigenetic regulation represents a crucial dimension of NB biology, shaping tumor progression, immune resistance, therapeutic response, and adaptation under selective pressure. Unlike genetic mutations that alter DNA sequence, epigenetic mechanisms, including DNA methylation, histone modifications, chromatin remodeling, noncoding RNAs, and higher-order chromatin architecture, modify gene expression programs without permanent genomic alteration [31]. NB is characterized by relatively low mutational burden compared to adult cancers, making epigenetic dysregulation a major driver of tumor plasticity, lineage commitment, and immune evasion [64]. Insights from recent analyses emphasize that NB progression involves close interplay between oncogenic programs, the epigenome, and the TME, all converging to support tumor survival while undermining antitumor immunity [102]. Here, we review the epigenetic mechanisms that underlie immune escape in NB and highlight therapeutic strategies that target these pathways (Table 2).

### 6.1. Epigenetic Alterations Underlying NB Progression and Immune Resistance

#### 6.1.1. Epigenetic Rewiring as a Driver of NB Progression

Recent research indicates that NB cells frequently adopt developmental programs governed by transcription factors and epigenetic states that mirror sympathoadrenal lineage differentiation [126]. These programs confer tumor cells with aggressive phenotypes, stem-like properties, and enhanced survival capacity. Epigenetic regulators such as chromatin modifiers, DNA methyltransferases, histone deacetylases, and protein degraders act in concert with NB-specific oncogenes including MYCN to sustain proliferative and metastatic behavior [31,102]. Because NB arises from NCCs, its chromatin landscape is inherently dynamic, allowing transitions between cellular states with differing immunological properties. HR-NB, in particular, shows epigenetic deregulation driven transcriptomic signatures steer repression of differentiation pathways, persistence of progenitor-like states, activation of survival and stress-response pathways, reduced immunogenicity and antigen presentation, and resistance to cytotoxic therapies [127]. These epigenetic shifts underpin NB heterogeneity, treatment resistance, and TIME.

#### 6.1.2. Epigenetic Suppression of Immune-Related Gene Expression

A central mechanism of TIME in NB involves epigenetic silencing of genes required for effective antitumor immunity, including those encoding antigen-presentation machinery, inflammatory cytokines, chemokines responsible for immune recruitment, and regulators of innate immune signaling [31]. According to the epigenome-microenvironment study, NB progression is shaped by chromatin-mediated modulation of tumor–immune interactions, creating a phenotype in which immune pathways are repressed [128]. Epigenetic repression may affect MHC class I genes, limiting T_c_ recognition; interferon-stimulated genes, reducing antiviral-like immune activation; chemokines such as CXCL9/CXCL10, which are needed for T_c_ infiltration; NK cell-activating ligands, impairing innate immune surveillance; and stress-induced pathways important for immunogenic cell death [52]. These changes reflect an integrated immune-evasive strategy driven not solely by oncogenes, but by epigenetic mechanisms that tune the immune phenotype of tumor cells.

#### 6.1.3. Epigenetic Regulation of the TME

The epigenetic landscape of NB not only regulates tumor-intrinsic gene expression but also alters the TME, particularly, with studies iterating that epigenetic mechanisms strongly influence how tumors interact with immune, stromal, and myeloid cells [102]. Key TME-related epigenetic processes include:

Suppression of immune cell activation—Epigenetic downregulation of cytokines and co-stimulatory molecules reduces immune infiltration and weakens effector cell function. NK cells and T_c_ encountering NB cells often face an environment lacking proper activating signals due to epigenetic silence [31,64].
Induction of immunosuppressive populations—Altered tumor-secreted factors, regulated epigenetically, promote expansion of suppressive cell types such as Tregs, MDSCs, and TAMs [10].
Remodeling of ECM and Stromal Compartments—Epigenetic regulators modulate genes involved in ECM deposition and stromal remodeling, which in turn affects immune cell trafficking and antigen presentation [129].

These processes create a self-reinforcing immune-suppressive ecosystem.

### 6.2. MYCN and Epigenetic Programming in NB-TIME

Among epigenetically regulated oncogenes, MYCN remains the dominant driver of NB pathogenesis and a central architect of the tumor–immune microenvironment. MYCN amplification, a hallmark of HR-NB, is tightly linked to widespread epigenetic remodeling that shapes tumor identity, metabolic state, and immune visibility [130]. Multiple studies demonstrate that MYCN interacts with chromatin-remodeling complexes, histone-modifying enzymes, and DNA-methylation machinery to enforce transcriptional programs that suppress immune activation [52]. Through these epigenetic partnerships, MYCN directly represses antigen-presentation genes, including components of the MHC-I pathway; downregulates type I and II interferon signaling; and alters transcription of innate-immunity regulators such as pattern-recognition receptors and cytokine-response genes. MYCN-driven epigenetic programming also maintains an undifferentiated, stem-like tumor state, which is inherently less immunogenic and more resistant to immune-mediated clearance. This lineage-locked phenotype contributes to poor responsiveness to IMT, including checkpoint blockade and cellular therapies [52]. Furthermore, MYCN coordinates metabolic rewiring, enhancing glycolysis, glutamine dependence, and redox buffering, that reinforces TIME by creating a metabolically hostile tumor immune microenvironment. Collectively, these findings position MYCN as an epigenetic hub that integrates proliferative, metabolic, and immune-suppressive circuits [24]. By shaping chromatin accessibility and transcriptional output, MYCN establishes a tumor ecosystem optimized for growth and immune escape [24]. Targeting MYCN-dependent epigenetic machinery therefore represents a promising strategy to re-sensitize NB to IMT and restore antitumor immune surveillance.

### 6.3. Epigenetic Alterations Contributing to Treatment Resistance

Epigenetic plasticity is increasingly recognized as a major driver of treatment resistance in NB, enabling tumor cells to dynamically reprogram their identity and survive therapeutic pressure. HR-NB tumors frequently undergo epigenetic reprogramming following CT, RT, or IMT, shifting toward mesenchymal-like or NCC-like states that are less differentiated, less immunogenic, and more stress-adapted [131]. These therapy-induced state transitions are orchestrated by chromatin-remodeling complexes, histone-modifying enzymes, and DNA-methylation changes that collectively reshape transcriptional output. A key feature of this adaptive process is the activation of stress-response transcriptional circuits, including AP-1, NF-κB, and ATF family factors, which are enabled by permissive chromatin landscapes established during treatment exposure [132]. These circuits promote survival under oxidative, metabolic, and inflammatory stress, allowing NB cells to withstand cytotoxic therapies. Epigenetic rewiring also disrupts differentiation pathways, reinforcing stem-like phenotypes that exhibit reduced antigen presentation and diminished susceptibility to immune-mediated killing [131]. Furthermore, epigenetically driven upregulation of drug-resistance genes, including efflux transporters, DNA-repair enzymes, and anti-apoptotic regulators, contributes directly to chemoresistance [133]. Importantly, many of these resistance mechanisms converge on epigenetic remodeling, highlighting why epigenetic regulators have emerged as compelling therapeutic targets. Inhibitors of HDACs, BET proteins, and PRC2 components show promise in reversing resistant states and restoring treatment sensitivity [134]. Collectively, these findings underscore that epigenetic plasticity is not merely a consequence of therapy but a central mechanism enabling NB persistence and relapse.

### 6.4. Potential Epigenetic Therapeutic Targets in NB

#### 6.4.1. Histone Deacetylase (HDAC) Inhibitors

HDACs are frequently dysregulated in NB, where they contribute to transcriptional repression of genes involved in differentiation, immune activation, and tumor suppression. HDAC-mediated chromatin compaction silences key components of antigen-presentation pathways and interferon signaling, reinforcing the immune-cold phenotype characteristic of HR-NB [103]. HDAC inhibitors (HDACi) counteract these effects by promoting a more open chromatin state, enabling re-expression of lineage-specific differentiation programs and enhancing tumor immunogenicity. Preclinical studies demonstrate that HDACi promote NB cell differentiation, restore interferon-responsive transcriptional networks, and increase MHC class I expression, thereby improving tumor visibility to CTLs [31]. HDACi also modulate the TME by reducing immunosuppressive cytokine production and enhancing T-cell and NK-cell activation. These immune-stimulatory effects provide a strong rationale for combining HDACi with IMTs, including checkpoint blockade and adoptive cell therapies, to improve antitumor immune recognition [31,64]. Several HDAC inhibitors, such as vorinostat, panobinostat, and entinostat, have shown promise in early NB studies, particularly when used in combination with agents that target MYCN, DNA-damage pathways, or immune checkpoints [104]. Collectively, these findings position HDAC inhibition as a compelling epigenetic strategy to reprogram NB cells toward a more differentiated, immunogenic, and therapy-responsive state.

#### 6.4.2. DNA Methyltransferase (DNMT) Inhibitors

DNMTs play a central role in establishing and maintaining the aberrant methylation patterns characteristic of HR-NB. Hypermethylation of promoter regions in antigen-presentation genes, interferon-response pathways, and tumor-suppressor loci contributes to the profoundly immune-silent phenotype of NB [133]. DNMT inhibitors (DNMTi), such as decitabine and azacitidine, counteract these effects by inducing global and locus-specific DNA hypomethylation, thereby reactivating silenced immune and differentiation programs. Preclinical studies show that DNMTi restore MHC class I expression, enhance type I interferon signaling, and increase transcription of pattern-recognition receptors and chemokines that recruit CTLs [105,106]. These epigenetic changes improve tumor immunogenicity and sensitize NB cells to T_c_-mediated killing. DNMTi also promote partial tumor-cell differentiation, reducing the stem-like, therapy-resistant states that emerge under treatment pressure. Importantly, DNMT inhibition can reverse epigenetically driven resistance mechanisms by reactivating apoptotic pathways and reducing expression of drug-efflux transporters. Combination strategies pairing DNMTi with IMTs, such as checkpoint blockade or adoptive NK-cell therapy, are supported by growing evidence that DNMTi enhance immune visibility and reduce suppressive signaling within the NB TME [105,106]. Collectively, DNMT inhibitors represent a promising class of epigenetic therapeutics capable of reprogramming NB cells toward a more immunogenic and therapy-responsive state.

#### 6.4.3. Bromodomain and Extraterminal Domain (BET) Protein Inhibitors

BET proteins are a family of epigenetic “reader” proteins that regulate gene expression by binding to acetylated histones and transcription factors, serving as scaffolds to recruit regulatory complexes that drive transcription and cellular development. BETs, particularly BRD4, are key epigenetic readers that regulate transcriptional elongation at super-enhancers controlling oncogenic, metabolic, and immune-evasive programs in NB. HR-NB frequently exhibits BRD4-driven activation of MYCN, inflammatory stress pathways, and chromatin states that suppress antigen presentation and interferon signaling [107]. BET inhibitors (BETi) disrupt BRD4 binding to acetylated chromatin, leading to broad transcriptional reprogramming that reduces tumor proliferation and enhances immune visibility. Preclinical studies demonstrate that BETi downregulate MYCN, attenuate survival signaling, and restore expression of immune-stimulatory genes, including components of the MHC-I pathway and interferon-response networks [108]. BET inhibition also reduces transcription of immunosuppressive cytokines and chemokines, thereby reshaping the TME toward a more pro-inflammatory state. These effects position BETi as promising agents to enhance antitumor immunity. Importantly, BETi synergizes with IMTs. By increasing tumor immunogenicity and reducing suppressive signaling, BET inhibitors improve T-cell and NK-cell recognition of NB cells and sensitize tumors to checkpoint blockade [108]. Several BET inhibitors, including JQ1, OTX015, and newer clinical-grade compounds, have shown strong preclinical activity, particularly in MYCN-amplified models [109,110]. Collectively, BET inhibitors represent a powerful epigenetic strategy capable of simultaneously targeting oncogenic transcription, metabolic stress responses, and immune evasion in NB.

#### 6.4.4. Enhancer of Zeste Homolog 2 (EZH2) Inhibitors

EZH2 is the catalytic subunit of the Polycomb Repressive Complex 2 (PRC2), responsible for depositing the repressive histone mark H3K27me3. Polycomb repressive complexes (PRC1/PRC2) epigenetically maintain stem-like, undifferentiated cell states, which in NB support cancer stem cell-like populations that promote immune suppression by limiting differentiation and sustaining tumor immune evasion programs [135]. In NB, EZH2 is frequently overexpressed and plays a central role in maintaining undifferentiated, proliferative, and immune-silent tumor states. EZH2-mediated chromatin repression silences genes involved in antigen presentation, interferon signaling, and neuronal differentiation, contributing to the aggressive phenotype of HRNB [112]. EZH2 inhibitors (EZH2i), including tazemetostat and next-generation PRC2-targeting compounds, reverse these repressive chromatin states by reducing H3K27me3 levels and reactivating silenced immune and lineage-specific programs. Preclinical studies demonstrate that EZH2i promote NB cell differentiation, restore MHC-I expression, and enhance type I interferon responses, thereby increasing tumor immunogenicity [111]. EZH2 inhibition also disrupts PRC2-dependent survival pathways, sensitizing NB cells to CT and targeted agents. Importantly, EZH2i shows strong synergy with IMTs. By lifting epigenetic repression of antigen-presentation machinery and inflammatory signaling, EZH2 inhibitors improve T_c_ recognition and enhance responses to checkpoint blockade [52,136]. Collectively, EZH2 inhibitors represent a promising therapeutic strategy to counteract the epigenetic rigidity of HR-NB, reprogramming tumors toward more differentiated, immune-responsive, and therapy-sensitive states.

#### 6.4.5. Lysine Specific Demethylase 1 (LSD1) Inhibitors

LSD1 (also known as KDM1A) is a key epigenetic regulator that removes mono- and dimethyl marks from H3K4 and H3K9, thereby controlling transcriptional programs essential for NB cell identity and TIME. HR-NB frequently exhibits elevated LSD1 activity, which reinforces undifferentiated, stem-like tumor states and suppresses genes involved in antigen presentation, interferon signaling, and neuronal differentiation [113].Through its interactions with transcription factors such as MYCN and components of the CoREST complex, LSD1 helps maintain the immune-cold phenotype characteristic of aggressive NB [114]. LSD1 inhibitors (LSD1i) disrupt these repressive chromatin programs, leading to reactivation of differentiation pathways, increased MHC-I expression, and enhanced interferon-responsive gene transcription. These changes improve tumor immunogenicity and sensitize NB cells to CTLs [115]. LSD1 inhibition also reduces expression of survival and stemness-associated genes, weakening the therapy-resistant states that emerge after chemotherapy or targeted therapy. Importantly, LSD1i shows strong synergy with IMTs. By lifting epigenetic repression of immune-stimulatory pathways, LSD1i enhance T_c_ and NK-cell recognition and improve responses to checkpoint blockade [116]. Several clinical-grade LSD1i, such as seclidemstat and iadademstat, are now being evaluated for pediatric solid tumors, including NB [117,118]. Collectively, LSD1 inhibitors represent a promising strategy to reprogram NB toward more differentiated, immune-responsive, and therapy-sensitive states.

#### 6.4.6. Polycomb Repressive Complex 1 (PRC1) Inhibitors

PRC1 is a key chromatin-modifying complex that works in concert with PRC2 to maintain transcriptionally repressive states through H2A ubiquitination (H2AK119ub1). In NB, PRC1 components, particularly BMI1 and RING1B, are frequently overexpressed and contribute to the maintenance of stem-like, undifferentiated tumor states associated with HR disease. PRC1-mediated repression silences genes involved in neuronal differentiation, antigen presentation, and interferon signaling, reinforcing the immune-cold phenotype characteristic of NB [119,120]. PRC1 inhibitors (PRC1i), including emerging BMI1-targeting compounds, disrupt these repressive chromatin programs by reducing H2A ubiquitination and reactivating silenced developmental and immune pathways [121]. Preclinical studies demonstrate that PRC1 inhibition promotes NB cell differentiation, reduces self-renewal capacity, and restores expression of immune-stimulatory genes, including components of the MHC-I pathway. These effects enhance tumor immunogenicity and weaken the therapy-resistant states that arise under treatment pressure [120]. Importantly, PRC1i shows potential synergy with IMTs. By lifting repression of antigen-presentation machinery and inflammatory signaling, PRC1 inhibitors improve T_c_ recognition and may sensitize NB tumors to checkpoint blockade [121]. Although PRC1-targeting drugs remain in early development, their ability to modulate lineage identity and immune visibility positions them as promising additions to the epigenetic-therapy landscape.

#### 6.4.7. Histone Acetyltransferase (HAT) Activators

HATs are key epigenetic writers that deposit acetyl groups on lysine residues of histone tails, creating an open chromatin configuration that promotes transcriptional activation. In NB, dysregulated HAT activity, often driven by oncogenic signaling or chromatin-repressive complexes, contributes to the silencing of genes involved in differentiation, antigen presentation, and immune activation [123]. Restoring HAT function through HAT activators or HAT-stabilizing compounds has emerged as a promising strategy to counteract the transcriptional rigidity of HR-NB. HAT activators enhance acetylation at promoters and enhancers of lineage-specific and immune-stimulatory genes, leading to reactivation of differentiation programs, increased MHC class I expression, and improved interferon-responsive transcription [123]. These changes promote a more immunogenic tumor phenotype and sensitize NB cells to CTLs. HAT activation also antagonizes the effects of repressive epigenetic regulators such as HDACs, PRC2, and LSD1, helping to reverse therapy-induced dedifferentiation and resistance. Preclinical studies suggest that HAT activators synergize with IMTs and targeted agents by enhancing chromatin accessibility at immune-related loci and reducing transcriptional dependence on oncogenic drivers like MYCN [122]. Although still in early development, HAT-activating compounds represent an emerging class of epigenetic therapeutics capable of reprogramming NB cells toward more differentiated, immune-responsive, and therapy-sensitive states.

#### 6.4.8. Protein Degraders (e.g., PROTACs)

Targeted protein degradation technologies, most notably PROTACs (Proteolysis-Targeting Chimeras), represent an emerging therapeutic strategy with strong relevance for NB, particularly in targeting epigenetic and transcriptional regulators that are difficult to inhibit with conventional small molecules [124]. PROTACs function by recruiting an E3 ubiquitin ligase to a target protein, leading to its ubiquitination and proteasomal degradation. This catalytic mechanism enables efficient removal of oncogenic drivers, including epigenetic regulators such as BRD4, HDACs, and components of the Polycomb complexes [124]. In NB, PROTAC-mediated degradation of BRD4 has shown potent suppression of MYCN-driven transcriptional programs, reduced tumor proliferation, and reactivation of immune-stimulatory pathways [125]. Unlike traditional inhibitors, degraders eliminate both enzymatic and scaffolding functions of their targets, producing deeper transcriptional reprogramming and more durable antitumor effects. Early studies also suggest that degraders targeting HDACs, LSD1, or EZH2 may overcome resistance mechanisms that arise from compensatory epigenetic rewiring [125]. Importantly, protein degraders can enhance tumor immunogenicity by restoring antigen-presentation machinery, increasing interferon signaling, and reducing immunosuppressive cytokine production. These effects position PROTACs as promising partners for IMTs, including checkpoint blockade and NK-cell-based approaches [137]. Although still in preclinical development for pediatric solid tumors, protein degraders represent a powerful next-generation strategy to dismantle the epigenetic architecture that sustains HR-NB.

### 6.5. Combining Epigenetic Therapy with IMT

Epigenetic therapies are increasingly being explored as immune-sensitizing agents. Combining epigenetic modulation with IMTs (e.g., anti-GD2, checkpoint inhibitors, NK-cell-based therapies) offers synergistic benefits. The combination strategies are aimed to increase tumor antigen expression, enhance cytokine and chemokine signaling, improve T_c_ infiltration into tumors, restore NK cell ligands, and reverse TME-driven immune suppression [138,139]. These approaches align with precision oncology efforts to create individualized combination regimens tailored to epigenetic and immune profiles.

Overall, epigenetic dysregulation plays a profound role in NB progression and immune evasion. Through chromatin remodeling, DNA methylation, histone modification, and transcriptional reprogramming, NB cells silence immune activation pathways, suppress antigen presentation, and remodel the TME to support survival. These epigenetic adaptations drive tumor plasticity, enabling NB to resist CT, evade immune surveillance, and adopt aggressive phenotypes. Importantly, epigenetic mechanisms are not only drivers of disease but also actionable therapeutic targets. Emerging epigenetic therapies, including HDAC and DNMT inhibitors, BET inhibitors, and protein degraders, offer promising tools to restore immune visibility and enhance responsiveness to IMT. By integrating epigenetic modulators into modern IMT frameworks, there is potential to transform the treatment landscape for HR-NB. Continued multi-omics research will be critical to identifying optimal therapeutic combinations and overcoming the entrenched immune resistance that characterizes this devastating pediatric cancer.

## 7. Exosome-Mediated Immune Modulation

Exosomes, nano-sized extracellular vehicles (EVs) generated by virtually all cell types, represent one of the most potent non-cellular mechanisms of immune regulation in the TME [29]. In NB, exosomes derived from tumor cells and CSCs profoundly influence tumor progression, metastasis, and the immune landscape. Through the transfer of proteins, lipids, mRNAs, microRNAs, and other regulatory molecules, NB TDEs actively reprogram local and systemic immunity to promote immune evasion. Recent evidence highlights exosome-dependent immune modulation plays a fundamental role in shaping NB disease outcome [44,45]. TDEs suppress T and NK cell activation, impair DC maturation, expand immunosuppressive myeloid populations, and enhance macrophage reprogramming, forming an immunologically “cold” environment resistant to IMT. CSC-derived exosomes amplify these effects by strengthening immunosuppressive pathways and enhancing tumor aggressiveness [44,45].

### 7.1. Biological Basis of Exosome-Mediated Communication in NB

**Figure 6:**
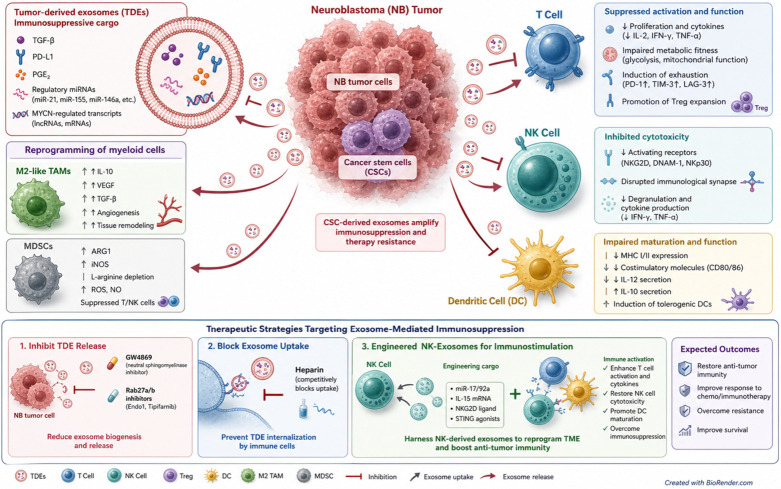
***Exosome-mediated immune modulation in NB-TME.*** Central NB tumor cells and cancer stem cells (CSCs) release TDEs (depicted as nano-vesicles) enriched with immunosuppressive cargo including TGF-β, PD-L1, PGE2, regulatory miRNAs, and MYCN-regulated transcripts. These TDEs suppress T_c_ (blue) activation, proliferation, cytokine production (↓IL-2, IFN-γ, TNF-α), and metabolic fitness while promoting Treg expansion; inhibit NK cell (teal) cytotoxicity via downregulated activating receptors (NKG2D, DNAM-1), disrupted immunological synapses, and reduced granule release; impair dendritic cell (DC; yellow) maturation by blocking MHC I/II, costimulatory molecules (CD80/86), and IL-12 secretion while favoring tolerogenic phenotypes and IL-10 output. TDEs also reprogram myeloid cells, driving M2-like TAMs (green; ↑IL-10, VEGF, angiogenesis) and MDSC (gray; ↑ARG1, iNOS, L-arginine depletion) expansion. CSC-derived exosomes amplify suppression and therapy resistance. Therapeutic strategies (bottom) include TDE release inhibition (GW4869, Rab27 inhibitors), uptake blockade (heparin), and engineered NK-exosomes for immunostimulation.

Exosomes are small EVs, typically 30–150 nm in diameter, generated through the endosomal-multivesicular body (MVB) pathway and released upon fusion of MVBs with the plasma membrane. These vesicles function as highly efficient intercellular messengers, enabling NB cells to shape their microenvironment through the transfer of proteins, lipids, metabolites, and nucleic acids [29,140]. In NB, exosome-mediated communication is a major mechanism by which tumor cells coordinate growth, invasion, and immune evasion. NB cells actively release TDEs enriched with oncogenic drivers, MYCN-regulated transcripts, immunosuppressive cytokines, and microRNAs that modulate both tumor and stromal compartments [44,45]. Stromal cells, including TAMs, mesenchymal stromal cells, and fibroblasts, also exchange vesicles that reinforce tumor proliferation, angiogenesis, and immune tolerance. Notably, CSCs produce exosomes with particularly potent cargo, including stemness-associated transcription factors, metabolic regulators, and immune-suppressive molecules that promote therapy resistance and tumor regeneration [44,45]. TDE-mediated immune suppression is a central barrier to effective IMT in NB. Exosomes downregulate antigen-presentation pathways, inhibit NK-cell cytotoxicity, impair T_c_ activation, and promote expansion of immunosuppressive myeloid populations [29]. These mechanisms contribute to the limited efficacy of anti-GD2 therapy, checkpoint blockade, and adoptive NK- or T_c_ approaches in HR-risk NB. Collectively, exosome-mediated signaling represents a fundamental biological axis through which NB orchestrates tumor progression and immune escape, underscoring its importance as both a biomarker source and therapeutic target (Figure 6).

### 7.2. TDEs and Their Role in Immune Suppression

TDEs are increasingly recognized as critical mediators of immune evasion in NB. These nanoscale extracellular vesicles transport a complex cargo of proteins, lipids, metabolites, and nucleic acids that profoundly reshape the tumor immune microenvironment [45]. In HR-NB, TDEs contribute to immune escape by dampening CTL responses, promoting immunosuppressive cell populations, and impairing immune cell metabolic fitness. Collectively, these effects represent a major barrier to effective T_c_- and NK cell-based IMTs [29,45].

#### 7.2.1. Suppression of T_c_ Activity by NB-Derived Exosomes

One of the primary immunosuppressive functions of NB-derived TDEs is the inhibition of T_c_ activation and effector function. TDEs directly interfere with TCR signaling pathways, resulting in attenuated activation of both CD8^+^ CTLs and CD4^+^ helper T_c_ [29]. This interference occurs through delivery of inhibitory ligands, immunosuppressive cytokines, and regulatory microRNAs that collectively blunt antitumor immunity. Similar mechanisms have been extensively described across solid tumors, reinforcing the relevance of these findings to NB [29,141].

#### 7.2.2. Inhibition of T_c_ Proliferation and Cytokine Production

NB-derived TDEs carry multiple immunosuppressive molecules capable of reducing T_c_ proliferation and cytokine secretion. These vesicles have been shown to diminish interleukin-2 (IL-2) production, a key cytokine required for T_c_ clonal expansion and survival, while simultaneously suppressing effector cytokines such as IFN-γ and TNF-α that are essential for CTL-mediated tumor killing [29]. Exosomal cargo, including TGF-β and inhibitory microRNAs, disrupts proximal TCR signaling and downstream transcriptional programs, thereby preventing effective tumor-directed immune responses [142,143].

#### 7.2.3. Disruption of T_c_ Metabolic Fitness

Although not always explicitly framed in metabolic terms, the functional paralysis of T_c_ induced by TDEs is consistent with exosome-mediated metabolic reprogramming. Effective T_c_ activation requires a metabolic shift toward aerobic glycolysis and robust mitochondrial function. TDE-mediated suppression of TCR signaling and cytokine production indirectly limits this metabolic reprogramming, leading to exhausted or anergic T_c_ states. Emerging evidence demonstrates that TDEs can alter immune cell glucose uptake, mitochondrial oxidative phosphorylation, and amino acid availability, supporting the concept that NB-derived exosomes similarly impair T_c_ metabolic fitness [45,141].

#### 7.2.4. Expansion of Immunosuppressive T_c_ Subsets

In addition to suppressing effector T_c_, TDEs promote the expansion and functional reprogramming of Tregs. The TDE cargo skews immune cell differentiation toward immunosuppressive phenotypes, contributing to a tolerogenic tumor microenvironment [29]. Increased Treg frequency further suppresses CTL activity through cytokine-dependent and contact-mediated mechanisms, compounding the immune inhibitory effects exerted by TDEs. Similar TDE-driven Treg expansion has been described across multiple solid cancers, reinforcing the generalizability of this mechanism [142].

#### 7.2.5. NK Cell Suppression by TDEs

NK cells play a crucial role in NB immune surveillance due to frequent downregulation of MHC class I on tumor cells. Despite this vulnerability, NB-derived TDEs markedly impair NK cell activity. TDEs suppress NK cell activation, proliferation, and cytotoxicity, significantly reducing their capacity to eliminate NB cells [29]. Exosomal factors such as prostaglandin E2 (PGE2), TGF-β, and inhibitory microRNAs are implicated in dampening NK cell effector functions [142].

#### 7.2.6. Downregulation of NK Cell Activating Receptors

TDEs also reduce surface expression of key NK cell activating receptors, including NKG2D, DNAM-1, and NKp30. While NB-specific data are limited, this mechanism is well documented across multiple tumor types and is highlighted as a relevant pathway in NB immune escape [29]. Loss of these receptors compromises NK cell recognition of stressed or malignant cells, further limiting tumor clearance [36,142].

#### 7.2.7. Interference with NK–Tumor Immunological Synapse Formation

Beyond receptor downregulation, TDEs interfere with cytoskeletal rearrangements required for stable immunological synapse formation between NK cells and tumor targets. Disruption of actin dynamics and lytic granule polarization impairs efficient delivery of cytotoxic molecules, resulting in incomplete or failed tumor cell killing. This mechanism represents a final common pathway through which TDEs undermine NK cell-mediated immunity and is considered a major obstacle to NK-based therapies in NB [29,36].

Altogether, TDE-mediated suppression of both T_c_ and NK cell immunity emerges as a dominant mechanism of immune escape in NB, highlighting exosome targeting as a promising adjunct to IMT strategies.

### 7.3. Inhibition of DC Maturation by TDEs

DCs are central orchestrators of adaptive immunity, acting as professional antigen-presenting cells that bridge innate and adaptive immune responses [144]. In NB, however, TDEs strongly impair DC differentiation, maturation, and function, thereby disrupting one of the earliest and most critical checkpoints of antitumor immune activation. DC suppression as a core mechanism by which TDEs establish an immunosuppressive TME, contributing to resistance against IMT approaches [29,144].

#### 7.3.1. Impaired Antigen Presentation

A defining feature of TDE-mediated DC dysfunction is impaired antigen presentation. TDEs interfere with the maturation of immature DCs by preventing the upregulation of MHC class I and II molecules, as well as essential costimulatory ligands such as CD80, CD86, and CD40 [142]. This results in defective T_c_ priming and inadequate activation of tumor-specific T lymphocytes. TDE cargo, including TGF-β, PD-L1, and regulatory microRNAs, actively blocks DC maturation pathways, pushing DCs into a semi-mature or functionally inert state incapable of inducing effective T_c_ responses [141,142]. In NB, this suppression of DC antigen presentation eliminates a critical axis of immune surveillance and facilitates tumor immune escape.

#### 7.3.2. Reprogramming of DC Cytokine Output

Beyond surface molecule expression, TDEs profoundly rewire DC cytokine secretion profiles. Exposure to tumor exosomes skews DCs away from a pro-inflammatory, Th1-polarizing phenotype toward a tolerogenic state characterized by reduced IL-12 production and increased secretion of immunosuppressive cytokines such as IL-10 [90]. This cytokine imbalance diminishes CTL differentiation and favors immune tolerance rather than immunity. Such cytokine reprogramming has been consistently described in NB and is considered a key mechanism by which TDEs suppress long-term antitumor immune memory.

#### 7.3.3. Expansion of Tolerogenic Dendritic Cell Phenotypes

In the immunosuppressive NB TME, TDEs promote the differentiation of regulatory or tolerogenic DC subsets. These DCs actively suppress effector T_c_ responses and contribute to Treg expansion, reinforcing immune evasion loops. Blockade of TDE release or uptake restores DC immunostimulatory capacity and enhances responses to IMT, underscoring the central role of DC suppression in NB immune escape [29,142]. Overall, TDE-mediated inhibition of DC maturation represents a foundational mechanism of immune suppression in NB and a promising therapeutic target for restoring adaptive immune activation.

### 7.4. TDE-Mediated Expansion and Reprogramming of Myeloid Populations

Beyond their direct inhibitory effects on T_c_, NK cells, and dendritic cells, TDEs profoundly reshape the innate immune compartment by driving the expansion and functional reprogramming of immunosuppressive myeloid populations. In NB, this myeloid skewing contributes to the establishment of a tumor-permissive immune ecosystem that supports tumor growth, immune evasion, and resistance to IMT. TDE-driven modulation of macrophages and MDSCs represents a central axis of immune suppression in HR-NB [10,29,142].

#### 7.4.1. TAMs

Macrophages are among the most plastic immune cells within the NB TME. TDEs actively induce macrophage polarization toward an immunosuppressive, M2-like phenotype, thereby promoting tumor progression. Exosomal cargo, including TGF-β, prostaglandins, and regulatory microRNAs, reprograms macrophage transcriptional and metabolic pathways, favoring anti-inflammatory and pro-tumorigenic functions rather than tumoricidal activity [29,141]. M2-polarized TAMs exert multiple tumor-supporting effects. They secrete high levels of IL-10 and TGF-β, which suppress CTL and NK cell activation, inhibit antigen presentation, and reinforce immune tolerance. In parallel, TAMs promote angiogenesis, ECM remodeling, and metastatic dissemination through the release of VEGF, matrix metalloproteinases, and growth factors. Importantly, in NB, M2-like macrophages have been implicated in nurturing tumor stem cell niches, supporting MYCN-driven survival pathways, and protecting tumor cells from immune-mediated clearance and therapy-induced stress [142,145].

#### 7.4.2. MDSCs

TDEs drive the expansion and activation of MDSCs, further amplifying systemic and local immune suppression. TDEs deliver suppressive microRNAs and inflammatory mediators that enhance myelopoiesis while blocking terminal differentiation into immunostimulatory myeloid cells. Functionally, exosome-educated MDSCs upregulate arginase-1 (ARG1) and inducible nitric oxide synthase (iNOS), leading to depletion of L-arginine and production of NO and ROS. These metabolic alterations directly impair T_c_ proliferation, TCR signaling, and survival [45,142]. The systemic accumulation of MDSCs creates a hostile environment for IMT, limiting the efficacy of adoptive T_c_ transfer, NK cell-based therapies, and immune checkpoint blockade. Inhibition of TDE biogenesis or uptake reduces MDSC expansion and restores immune responsiveness, underscoring the therapeutic relevance of targeting this pathway in NB [29]. In summary, TDE-mediated reprogramming of macrophages and MDSCs represents a dominant mechanism by which NB enforces durable immune suppression, complementing T_c_, NK cell, and dendritic cell dysfunction and reinforcing resistance to IMT.

### 7.5. CSC-Derived Exosomes in NB: Amplifying Immune Suppression

CSCs constitute a specialized subpopulation within NB characterized by enhanced tumor-initiating capacity, self-renewal, metastatic competence, and resistance to conventional therapies. Increasing evidence indicates that CSCs secrete exosomes with a molecular cargo that is both quantitatively and qualitatively distinct from that of bulk tumor cells. CSC-derived exosomes (CSC-Exos) exert disproportionately strong immunomodulatory effects, introducing additional layers of immune dysfunction that significantly contribute to NB immune evasion and disease relapse [29,145].

#### 7.5.1. CSC-Exos Promote Deep Immune Reprogramming

CSC-Exos are enriched in stemness-associated transcription factors (e.g., SOX2, MYCN-regulated programs), oncogenic microRNAs, and epigenetic regulators that enable long-term immune reprogramming. Upon uptake by immune cells, these vesicles shift cellular differentiation and functional states toward immune suppression [29]. CSC-Exos promote polarization of macrophages toward highly suppressive M2-like phenotypes, reinforce inhibitory cytokine networks, and accelerate exhaustion programs in T_c_ and NK cells. Compared with bulk TDEs, CSC-Exos more effectively blunt immune effector differentiation while sustaining suppressive loops within the TME [29,43]. Through these mechanisms, CSC-Exos establish durable immune tolerance that facilitates tumor persistence and relapse following therapy.

#### 7.5.2. CSC-Exos and Immune Plasticity

A defining feature emphasized is the ability of CSC-Exos to induce “functional changes in immune cell subsets,” underscoring their role as drivers of immune plasticity. CSC-Exos promote the development of tolerogenic DCs with impaired antigen-presenting capacity, foster the expansion of TREGs, and suppress inflammatory cytokine signaling. These effects create a TME uniquely supportive of CSC self-renewal and survival. Importantly, CSC-Exos participate in bidirectional feedback loops: immune suppression enhances CSC fitness, while CSC activity increases tumor heterogeneity and phenotypic plasticity, further complicating immune control [29,43,145].

#### 7.5.3. CSC-Exos Enhance Resistance to IMT

Because CSCs are intrinsically therapy-resistant, their exosomes also contribute to resistance against immunotherapeutic interventions. CSC-Exos may disseminate drug-resistance molecules to non-stem tumor cells, disrupt immune synapse formation, and inhibit CTL engagement. These effects undermine the efficacy of NK cell-based therapies, T_c_-directed IMTs, and immune checkpoint blockade. CSC-ExOs acts as a major impediment to durable IMT responses in NB, reinforcing the need to target exosome biogenesis, release, or uptake as part of combinatorial treatment strategies [29,145].

### 7.6. Exosome-Targeted Therapeutic Approaches

Given the central role of TDEs in orchestrating immune suppression, tumor progression, and therapy resistance, targeting exosome biology has emerged as a promising therapeutic strategy in NB [29,145]. Since exosomes are not merely passive biomarkers but active regulators of immune dysfunction, making them actionable nodes for therapeutic intervention across multiple levels of tumor–immune crosstalk.

#### 7.6.1. Inhibition of TDE Release

One of the most direct strategies to counteract exosome-mediated immune evasion is pharmacologic inhibition of TDE biogenesis and secretion. Several agents have been identified to disrupt exosome release pathways. GW4869, a neutral sphingomyelinase inhibitor, blocks ceramide-dependent exosome formation and has been widely used in preclinical models to reduce TDE production [29]. Similarly, imipramine interferes with endosomal trafficking pathways, while inhibitors targeting Rab GTPases (e.g., Rab27a, Rab27b) disrupt vesicle docking and fusion [146]. By limiting the release of immunosuppressive vesicles, these approaches reduce the delivery of inhibitory cytokines, suppressive microRNAs, and metabolic regulators to immune cells, thereby partially restoring antitumor immunity [29]. Although still largely preclinical, the exosome-release inhibition as a rational adjuvant strategy to enhance IMT efficacy in NB.

#### 7.6.2. Blocking Exosome Uptake

An alternative and complementary approach involves preventing recipient immune cells from internalizing suppressive exosomal cargo. TDE uptake occurs through multiple mechanisms, including clathrin-mediated endocytosis, macropinocytosis, and lipid raft-dependent membrane fusion [147]. Therapeutic strategies aimed at disrupting these pathways include the use of heparin and heparin analogues, which block exosome binding to cell-surface proteoglycans, as well as lipid raft disruptors and endocytosis inhibitors [148]. By limiting exosome internalization, these strategies preserve DC maturation, T_c_ activation, and NK cell cytotoxicity. Blocking uptake may offer greater specificity than globally inhibiting exosome release, particularly in the context of systemic immune modulation [29,149].

#### 7.6.3. Engineering NK-Derived Exosomes for IMT

A particularly innovative strategy is the therapeutic exploitation of immune cell-derived exosomes, especially those originating from NK cells. Unlike TDEs, NK-derived exosomes retain intrinsic cytotoxic and immune-activating properties and can be engineered to enhance antitumor efficacy. These vesicles can be loaded with stimulatory cytokines, cytotoxic molecules, or immune checkpoint inhibitors, enabling targeted delivery to tumor cells or immune effector populations [150]. Engineered NK exosomes have been shown to inhibit PD-L1 signaling on tumor cells, enhance T_c_ and NK cell activation, and elicit robust antitumor responses without the toxicity associated with live-cell therapies [150,151]. This approach reframes exosomes not solely as therapeutic targets but as next-generation cell-free immunotherapeutic agents with improved safety and translational potential.

#### 7.6.4. Therapeutic Roles of Normal-Tissue-Derived Exosomes (NDE)

Exosomes derived from nearby normal tissues, particularly immune and stromal cells, exhibit promising therapeutic potential in NB by enhancing anti-tumor immunity and modulating the TME. Immune cell-derived exosomes (e.g., NK, T_C_) can deliver cytotoxic molecules such as perforin and granzyme, thereby directly promoting tumor cell killing and immune activation [29]. In contrast to TDEs that suppress immune responses, normal-cell exosomes can restore immune function by reversing tumor-induced immunosuppression and reactivating CTLs. Additionally, these vesicles serve as efficient and biocompatible drug delivery systems, capable of transporting therapeutic cargo, including siRNAs, miRNAs, or small-molecule inhibitors targeting oncogenic drivers like MYCN, directly to tumor cells with reduced systemic toxicity [152]. NDEs also contribute to reprogramming the TME by modulating macrophages, dendritic cells, and cytokine signaling, thereby shifting it from an immunosuppressive to an immunostimulatory state [153]. Furthermore, they can transfer tumor-suppressive molecular cargo that inhibits proliferation and induces apoptosis in NB cells. Owing to their intrinsic stability, low immunogenicity, and natural targeting properties, these exosomes represent a versatile and attractive therapeutic platform. Overall, while TDE promotes immune evasion and disease progression, NDEs, especially from immune and stem cells, offer a multifaceted strategy to deliver therapeutics, restore immune surveillance, and reshape the NB microenvironment. Despite these advantages, clinical translation remains limited by challenges in large-scale production, cargo standardization, targeting specificity, and incomplete understanding of in vivo biodistribution and long-term safety.

#### 7.6.5. Biomarker and Precision Medicine Applications

Beyond therapeutic intervention, TDEs hold considerable promise as clinical biomarkers. Exosome cargo reflects tumor genotype, phenotype, and immune status, making them attractive candidates for noninvasive diagnostics. TDE signatures may serve as biomarkers for early disease detection, predictors of response to IMT, and indicators of MRD or relapse. Given the marked heterogeneity of NB, longitudinal profiling of circulating exosomes could enable dynamic risk stratification and guide personalized treatment strategies [145,154].

In conclusion, exosomes play a central and multifaceted role in NB immune evasion. TDEs suppress T_c_ activation, inhibit NK-cell cytotoxicity, and prevent DC maturation, thereby undermining both innate and adaptive immunity. They also reprogram myeloid populations toward immunosuppressive phenotypes, creating a microenvironment that favors tumor growth and therapy resistance. CSC-exos further amplify immunosuppressive pathways, contributing to NB relapse and treatment failure. Therapeutic approaches aimed at blocking exosome release or uptake, combined with engineered immune-activating exosomes, hold significant translational potential and may overcome current limitations of IMT in HR-NB.

## 8. Tumor–Immune Cell Interactions in NB

The defining feature of NB immunobiology is the profound dysfunction that emerges across multiple arms of the immune system, especially NK cells, T_c_, MDSCs and TAMs. Recent RNA-sequencing studies of NB have provided unprecedented clarity regarding these cellular interactions [12,15]. These landmark studies assessing pre and post CT, investigators identified distinct immune cell subsets, revealing the intrinsic and treatment-induced alterations that shape immune dysfunction in NB. This granular tumor profiling highlights a dynamic immunoregulatory network in which NK cells and T_c_ exhibit suppressed cytotoxicity and exhaustion, while myeloid populations expand into strongly immunosuppressive phenotypes [12]. Critically, these interactions converge upon specific immune checkpoint pathways, most notably the RD3–PD-L1, RD3–STING, RD3–NFκB, NECTIN2–TIGIT axes, identified as a major driver of immune impairment in NB [12,15]. Together, these findings illuminate how NB tumors orchestrate an immunosuppressive ecosystem capable of resisting both natural immune attack and therapeutically augmented immune responses.

### 8.1. NK Cell Dysfunction

NK cells are essential effectors of anti-tumor immunity, particularly in NB where MHC-I expression is frequently reduced. Under normal conditions, NK cells recognize and kill transformed or infected cells by detecting “missing-self” signals or via activating receptors that bind stress-induced ligands on tumor cells. In NB, however, NK cells are significantly impaired and exhibit a dysfunctional phenotype that evolves throughout the disease course and is modulated by CT [155,156].

#### 8.1.1. Reduced Cytotoxicity

A major finding is the marked reduction in NK-cell cytotoxic activity within NB tumors. NK cells isolated from both pre- and post-CT tumors express lower levels of cytotoxic effector molecules, including perforin and granzymes, which are essential for target-cell lysis. These NK cells also exhibit downregulated signaling pathways necessary for degranulation and cytotoxic granule release [155]. This loss of cytotoxic potential is accompanied by weakened expression of chemokines and receptors important for NK trafficking, further limiting their capacity to accumulate in tumor sites and engage malignant cells. Moreover, the NK-cell dysfunction is not static, and NK cytotoxicity fluctuates over the course of CT, with post-treatment samples alterations in NK subsets and signaling pathways that are further skewed toward dysfunction [156,157]. This dynamism highlights how cytotoxic impairment is both an intrinsic feature of NB immune evasion and a consequence of therapy-induced microenvironmental remodeling.

#### 8.1.2. Altered NK-Cell Phenotype

Beyond reduced cytotoxicity, NB-infiltrating NK cells exhibit a transcriptional phenotype consistent with exhaustion and chronic activation, including upregulation of multiple inhibitory receptors. High expression of TIGIT, an inhibitory receptor that binds the NECTIN2 ligand expressed on NB tumor cells was shown to be a central mediator of NK suppression [12]. Mechanistically, the NECTIN2–TIGIT interaction shuts down NK-cell function, inhibiting cytokine production and limiting responses to stress-induced ligands on tumor cells. Blocking TIGIT or combining TIGIT blockade with PD-L1 inhibition significantly enhanced NK-cell effector functions in preclinical models, resulting in improved tumor control [12]. These findings affirm that NK dysfunction is not merely correlational but causally linked to tumor-driven checkpoint signaling.

### 8.2. T_c_ Exhaustion and Dysfunctional Profiles

T_c_ plays a central role in adaptive immunity, and their activation is critical for generating durable anti-tumor responses. However, NB exhibit a profoundly exhausted and dysfunctional T_c_ profile that impairs cytotoxicity and reduces responsiveness to IMTs such as checkpoint inhibitors or T_c_-engaging therapies [23].

#### 8.2.1. T_c_ Exhaustion Pre-CT

T_c_ infiltrating untreated NB tumors exhibit features of exhaustion, characterized by upregulation of inhibitory receptors (e.g., TIGIT, PD-1); reduced expression of cytotoxic genes; lower cytokine secretion capacity; and impaired activation signals across TCR pathways [12]. These exhaustion markers suggest chronic antigen exposure, likely due to persistent tumor antigen presentation coupled with immune-suppressive signaling from the tumor and stromal compartments. Exhaustion phenotypes were particularly pronounced in CD8^+^ T_c_, which should ordinarily serve as critical cytotoxic responders to NB cells. T_c_ dysfunction was tied to the NECTIN2–TIGIT pathway, with TIGIT-expressing T_c_ showing severely diminished effector function [12,158]. This finding implicates NB-specific checkpoint ligand expression in driving the dysfunctional T_c_ program.

#### 8.2.2. Impact of CT on T_C_ Profiles

A striking aspect of NB immunobiology is the profound remodeling of T_c_ subsets following CT. When comparing pre- and post-CT tumors, studies found shifts toward more dysfunctional immune states; increased expression of exhaustion and suppressive markers; reduced presence of cytotoxic T_c_ subsets; and alterations in T_c_ metabolic and signaling pathways. CT induces both tumor and immune-cell death, but in NB it paradoxically deepens immune suppression by enriching phenotypes associated with chronic exhaustion [158]. This aligns with clinical experience, where relapses after induction therapy are associated with strong immune dysfunction and poor responses to subsequent IMTs. These findings suggest that T_c_ exhaustion in NB is both an intrinsic tumor-driven phenomenon and an extrinsic consequence of CT, creating a therapeutic obstacle that must be addressed through combination IMT strategies.

Recent studies identify RD3 as a critical regulator of T_c_-mediated immune surveillance in HR and therapy-resistant NB. Analysis of progressive NB demonstrates that acquired RD3 loss profoundly reshapes the tumor immune microenvironment, resulting in marked alterations in T_c_ abundance, activation state, and effector function. RD3-deficient tumors exhibit significantly reduced T_c_ infiltration along with diminished cytotoxic immune signatures, consistent with an immune-cold phenotype [15,37]. Mechanistically, RD3 loss disrupts antigen presentation machinery, including downregulation of MHC class I/II components and β2-microglobulin, thereby impairing T_c_ recognition of tumor cells. Concurrently, RD3 deficiency induces immune checkpoint molecules such as PD-L1 and CD276 and activates adenosinergic immunosuppressive pathways (CD73-A2AR), leading to T_c_ exhaustion and functional paralysis. These changes skew T_c_ profiles away from effector and memory phenotypes toward inactive or tolerized states, undermining sustained antitumor immunity [15,37]. Further, RD3 functions as a molecular switch integrating cellular identity and immunoediting, directly regulating transcriptional programs governing both tumor plasticity and T_c_ function. Restoration of RD3 reverses immune-silent programming, enhancing T_c_ activation, cytokine production, and cytotoxic capacity while promoting immune cell infiltration and tumor clearance in vivo. Collectively, these findings position RD3 as a master regulator of CT-steered modifications in T_c_ immune profiles in NB. Loss of RD3 enforces TIME by simultaneously impairing tumor immunogenicity and actively suppressing T_C_ function, providing a mechanistic link between therapy-induced plasticity and immune escape [15,37]. Targeting RD3-controlled pathways therefore represents a promising strategy to restore T_c_ immunity and enhance IMT responsiveness in progressive NB.

### 8.3. MDSCs and TAMs

Myeloid cells are central architects of the NB immune landscape. Unlike T_c_ and NK cells, which are suppressed or exhausted, myeloid populations expand and differentiate into potent immunosuppressive phenotypes that reinforce tumor progression and create a niche resistant to immune attack [10].

#### 8.3.1. Myeloid Cell-Mediated Immune Suppression

The single-cell study identified large populations of immunosuppressive myeloid cells, including MDSCs and TAMs, within NB tumors [12]. These cells contribute significantly to NB immune escape through secretion of immune-inhibitory cytokines; production of metabolites (e.g., ROS, NO) that inhibit T and NK activity; antigen presentation deficits; promotion of tumor angiogenesis; and remodeling of ECM to limit lymphocyte infiltration [10,12]. This immunosuppressive myeloid environment was found to be a defining characteristic of NB tumors.

#### 8.3.2. TAMs

TAMs in NB display a strongly pro-tumorigenic phenotype, exhibiting features analogous to M2-like macrophages, including high expression of anti-inflammatory cytokines; support for tumor growth and angiogenesis; and enhanced interactions with tumor cells that promote immune evasion. TAMs engage in paracrine signaling networks with tumor cells, supporting survival pathways such as ERK signaling. The macrophage–tumor interactions were particularly pronounced in CT-resistant states, suggesting that TAMs may play a role in mediating treatment failure [10,158].

#### 8.3.3. MDSCs

Transcriptional signatures consistent with MDSC biology, such as immunosuppressive metabolite production, antigen-presentation deficits, and inflammatory reprogramming, were observed within the myeloid compartment. These cells contribute to inhibition of T_c_ activation, suppression of NK-cell cytotoxicity, and expansion of TREG populations [10,159]. Their increased presence in post-therapy tumors supports a model in which CT further enriches immunosuppressive myeloid populations, intensifying immune evasion [10].

#### 8.3.4. Myeloid Reprogramming After CT

Studies have shown that macrophages expanded significantly following CT, adopting pro-angiogenic, metabolic, and immunosuppressive phenotypes. This remodeling was strongly correlated with poor outcome and reduced therapeutic response. The myeloid compartment therefore acts as both a driver of baseline immune suppression, and a dynamic mediator of therapy-induced immune remodeling [158,159]. This reinforces the need to incorporate myeloid-targeting strategies (e.g., CSF1R inhibitors, myeloid reprogrammers, metabolic modulators) into NB IMT regimens [10].

## 9. Influence of Genetic and Molecular Drivers on Immune Evasion in NB

NB exhibits profound clinical, biological, and immunological heterogeneity, much of which is shaped by its underlying genetic and molecular drivers. Among these, MYCN amplification, ALK, PI3K/AKT/mTOR, RAS-MAPK, etc., collectively, exert a powerful influence on the TME, modulating immune surveillance mechanisms, altering cytokine profiles, suppressing antigen presentation, and reprogramming both tumor and immune cell function [24,160]. Recent studies have emphasized that NB progression is not merely a consequence of autonomous tumor-cell proliferation but also of genetic-driven modulation of immune interactions. HR-NB, including those with MYCN amplification or ALK mutations, display strongly immunosuppressive microenvironments, characterized by impaired NK-cell and T_c_ activity, macrophage enrichment, and stromal remodeling [24]. These alterations are not incidental; they reflect coordinated signaling networks that tune the TME to favor immune escape.

### 9.1. MYCN Amplification and Immune Suppression

**Figure 7:**
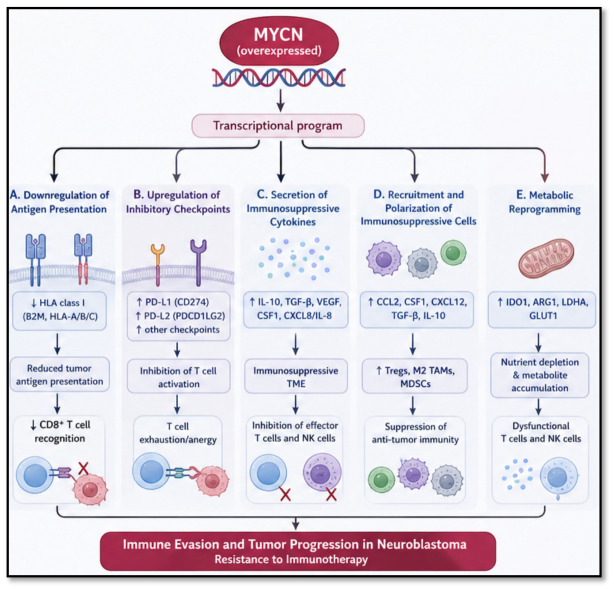
***MYCN Signaling Pathway Driving NB-TIME*.** Amplified MYCN drives a broad transcriptional program that modulates multiple immune-regulatory axes within NB- TME. MYCN suppresses expression of MHC class I components (e.g., HLA-A/B/C, β2-M), impairing tumor antigen presentation and reduced recognition by CD8^+^ CTLs; enhances the expression of immune checkpoint PD-L1 and PD-L2 leading to inhibition of T_C_ activation and promotion of T_C_ exhaustion or anergy; release cytokines (IL-10, TGF-β, VEGF, CSF1, CXCL8/IL-8), establishing an immunosuppressive TME and inhibit effector T_C_ and NK cell function; secrete chemokines (e.g., CCL2, CXCL12) that recruit and expand Tregs, M2-polarized TAMs, and MDSCs, thereby suppressing anti-tumor immunity; and alters tumor metabolism by upregulating IDO1, ARG1, LDHA, and GLUT1, leading to nutrient depletion and accumulation of immunosuppressive metabolites, which impair T_C_ and NK cell function.

MYCN amplification, present in ~20–25% of NB cases, is the single most powerful molecular predictor of poor prognosis. Numerous studies have demonstrated that MYCN-amplified tumors are highly aggressive, treatment-resistant, and prone to early metastasis [4,24]. However, beyond its well-known roles in proliferation, apoptosis resistance, and metabolic reprogramming, MYCN also directly orchestrates TIME mechanisms, shaping a TME hostile to immune surveillance. Such tumors often show reduced infiltration by immune effector cells, including NK cells and cytotoxic T_c_, and they develop stromal architectures that impede immune cell trafficking [24]. MYCN regulates the transcription of numerous immune-modulatory genes, directly shaping the immune landscape (Figure 7). Several mechanisms contribute to MYCN-mediated immune resistance:

Suppression of Antigen Presentation—MYCN-amplified NB cells downregulate antigen-processing and presentation machinery, reducing MHC-I expression and limiting CD8^+^ T-cell recognition [24].
Interference With NK-Cell Surveillance—Because MYCN-amplified tumors display low MHC-I, they might normally be more susceptible to NK-cell cytotoxicity. Further, MYCN promotes additional downstream changes, such as altered GD2 and immune-repressive signaling, that blunt NK-cell activation. This contributes to poor responsiveness to NK-based IMTs [24,161].
Promotion of an Immunosuppressive TME—MYCN hyperactivation stimulates tumor-associated stromal remodeling, creating niches enriched for immunosuppressive cells such as TAMs. These niches support tumor angiogenesis and ECM restructuring while releasing factors that suppress cytotoxic immunity [162].
Metabolic Immune Suppression—MYCN regulates metabolic pathways that influence immune cell viability and function. By shifting cellular metabolism toward glycolysis and altering mitochondrial networks, MYCN creates conditions in which immune effector cells are metabolically disadvantaged [162].

Collectively, these MYCN-driven mechanisms allow tumors to circumvent immune elimination, making MYCN a central regulator of NB TIME. To that end, IMTs including anti-GD2 antibodies and emerging CAR-T approaches, face particular challenges in MYCN-amplified tumors. These challenges arise because MYCN-positive tumors alter expression of IMT targets; inhibit T_c_ and NK-cell infiltration; promote checkpoint ligand upregulation; and maintain high levels of tumor plasticity that enable immune escape. Thus, MYCN-amplified NB represents a uniquely difficult subtype requiring combination strategies integrating immune, metabolic, and epigenetic therapies.

### 9.2. ALK Signaling and Immune Resistance in NB

Activating mutations in ALK represent one of the most frequent germline or somatic oncogenic drivers in NB, particularly in HR-NB. Beyond its well-established role in promoting tumor cell proliferation, survival, and resistance to apoptosis, accumulating evidence indicates that aberrant ALK signaling actively shapes the tumor immune microenvironment. ALK inhibitors constitute a major therapeutic axis in NB; however, ALK activity itself contributes to immune resistance by coordinating tumor-intrinsic and immune-extrinsic programs [163]. ALK signaling frequently cooperates with MYCN amplification to enhance transcriptional networks that suppress tumor immunogenicity. This cooperation results in reduced expression of antigen-presentation machinery and upregulation of immune inhibitory molecules, thereby limiting effective T_c_-mediated recognition of tumor cells. Clinically, ALK-mutant tumors are often associated with more aggressive phenotypes and diminished infiltration of CTLs, reinforcing the concept of ALK-driven immune exclusion [36]. In addition, ALK activation promotes metabolic reprogramming within tumor cells, including enhanced glycolysis and altered lipid metabolism, which indirectly impairs immune effector cell function. Such metabolic shifts reduce nutrient availability and generate immunosuppressive metabolites within the TME, weakening NK-cell cytotoxicity and impairing T_c_ activation and persistence. ALK-dependent metabolic adaptations create a hostile landscape for immune cell engagement, further reinforcing immune escape mechanisms [36,163]. Collectively, ALK mutation status influences both tumor aggressiveness and tumor–immune interactions. By integrating oncogenic signaling with immune suppression and metabolic rewiring, ALK serves as a critical contributor to NB TIME. These insights underscore the rationale for combining ALK-targeted therapies with IMT approaches to overcome resistance and restore antitumor immunity.

### 9.3. PI3K/AKT/mTOR Pathway and Immune Modulation

The PI3K/AKT/mTOR signaling pathway is a central regulator of cellular survival, growth, metabolism, and stress adaptation, and its aberrant activation is a hallmark of HR-NB. PI3K/AKT/mTOR as a major oncogenic hub downstream of RTKs and MYCN, coordinating angiogenesis, resistance to apoptosis, stress responses, and metabolic plasticity that collectively drive tumor progression. While these functions are classically tumor-intrinsic, PI3K/AKT/mTOR activation has profound indirect consequences for antitumor immunity [164]. Constitutive pathway signaling accelerates tumor metabolic programs, including enhanced glycolysis, amino acid uptake, and lipid synthesis. This metabolic hyperactivity creates a competitive microenvironment in which glucose, glutamine, and other nutrients become limiting for infiltrating T_c_ and NK cells, impairing their proliferation, cytokine production, and cytotoxic effector functions [165]. In parallel, PI3K/AKT/mTOR signaling promotes the expression of immunosuppressive molecules within the TME. Pathway activation enhances the secretion of inhibitory cytokines and growth factors, while also supporting transcriptional programs that increase immune checkpoint ligand expression, further dampening T_c_ activation and persistence. These effects reinforce immune exclusion and functional exhaustion of effector lymphocytes [165]. Finally, PI3K/AKT/mTOR signaling confers resistance to immunogenic cell death by stabilizing anti-apoptotic proteins and suppressing stress-induced danger signals that would otherwise activate innate immune sensing [165]. As this pathway intersects closely with MYCN and other oncogenic drivers, its sustained activation reinforces the immune-cold phenotype characteristic of HR-NB, highlighting PI3K/AKT/mTOR as a critical link between tumor metabolism, survival signaling, and immune escape.

### 9.4. RAS–MAPK Pathway and Immune Evasion

The RAS–MAPK signaling pathway represents a major oncogenic axis implicated in NB progression, tumor plasticity, and therapeutic resistance. Aberrant activation of the RAS-MAPK cascade promotes tumor cell proliferation, survival, and lineage reprogramming. Importantly, this pathway also exerts substantial influence on tumor–immune interactions, positioning it as a key contributor to immune evasion in HR-NB [6,166]. One major immune-modulatory consequence of RAS-MAPK activation is extensive remodeling of the tumor stroma and microenvironment. Enhanced MAPK signaling drives ECM deposition, angiogenesis, and fibroblast activation, collectively altering immune cell trafficking and reducing the physical accessibility of cytotoxic T_c_ and NK cells to tumor nests. This stromal reorganization promotes immune exclusion, a hallmark of IMT-resistant tumors. RAS-MAPK activation also induces stress-response and survival gene programs that dampen inflammatory signaling and suppress immune-mediated cytotoxicity. These transcriptional changes limit the responsiveness of tumor cells to immune effector mechanisms and blunt innate immune activation, further weakening antitumor immunity [166]. In addition, NB tumors with sustained RAS-MAPK signaling frequently transition toward mesenchymal-like states. This phenotypic shift is associated with reduced antigenicity, impaired antigen presentation, and resistance to T_c_-based IMTs. Mesenchymal NB states are increasingly recognized as immune-cold and therapy-refractory. Crucially, RAS-MAPK activation often co-occurs with MYCN amplification and intersects with ALK signaling in HR-NB [6,166]. This convergence of oncogenic pathways synergistically amplifies tumor aggressiveness and immune escape, reinforcing the concept that NB immune evasion arises from integrated signaling and transcriptional networks rather than a single dominant oncogene.

In conclusion, NB TIME is deeply intertwined with its genetic landscape. MYCN amplification establishes a transcriptional program that promotes immune suppression, stromal reorganization, metabolic reprogramming, and resistance to immune-based therapies. Meanwhile, activation of ALK, PI3K/AKT/mTOR, and RAS–MAPK pathways further enhances tumor adaptability, survival, and immune escape by shaping the metabolic state, stromal architecture, and cell-surface immunogenicity of NB tumors. Together, these oncogenic drivers create an environment in which NK cells and CTLs are unable to mount effective anti-tumor responses. Any future therapeutic strategy for HR-NB must address these molecular drivers to overcome immune resistance. This likely requires a combination of targeted inhibitors, metabolic modulators, and IMTs designed with deep awareness of genetic–immune crosstalk. Only by integrating molecularly targeted approaches with advanced IMT will it be possible to meaningfully improve outcomes for HR-NB.

## 10. IMTs for NB

IMT has become a critical component of modern treatment strategies for NB, particularly for patients with HR-NB who experience poor outcomes despite IMCT. Current approaches aim to harness and enhance the patient’s immune system to recognize and eliminate tumor cells, overcoming the intrinsic immune-evasive nature of NB. Among these, anti-GD2 monoclonal antibodies represent the most established and clinically successful IMT, significantly improving survival when combined with cytokines and isotretinoin. Additional strategies under active investigation include immune checkpoint blockade, adoptive cell therapies such as CAR-T and CAR-NK cells, cancer vaccines, and cytokine-based immune modulation [21,150,156]. However, immune-suppressive signaling networks, tumor heterogeneity, and a typically immune-cold TME limit the durability of these responses. Understanding how NB resists immune attack has therefore become central to optimizing current IMTs and developing effective combination strategies that restore robust antitumor immunity.

### 10.1. Anti-GD2 Therapy

Anti-GD2 monoclonal antibody therapy has become a foundational component of treatment for HR-NB, representing one of the few IMT strategies that has demonstrably improved survival in this pediatric population. GD2, a disialoganglioside expressed abundantly on NB cells but minimally on healthy tissues, provides an attractive therapeutic target for antibody-mediated cytotoxicity. Clinical integration of anti-GD2 antibodies such as dinutuximab has led to measurable improvements in EFS and OS, validating the rationale for leveraging GD2-directed immune responses. Yet despite its success, anti-GD2 therapy does not uniformly lead to durable tumor control, and a significant number of patients relapse. Recent high-resolution genomic and transcriptomic analyses have begun to clarify why anti-GD2 therapy’s efficacy remains limited, revealing that tumor-intrinsic immune-evasive processes deeply influence therapeutic responsiveness [167]. One of the central observations emerging is that the effectiveness of anti-GD2 IMT is strongly linked to the maintenance of specific developmental phenotypes within the tumor. Analysis of 840 NB tumors demonstrated that patients who responded favorably to multimodality therapy containing anti-GD2 antibodies exhibited gene-expression signatures associated with mature noradrenergic sympathoadrenal cell states [167]. This corresponds to a developmental lineage that preserves differentiation programs and metabolic features conducive to robust inflammatory signaling and enhanced susceptibility to ADCC. Conversely, tumors that lacked these developmental phenotypes showed markedly poorer treatment outcomes, indicating that deviations from this lineage undermine the immune recognition and effector mechanisms required for anti-GD2 efficacy.

A key driver of this loss of favorable developmental states is chromosomal instability, particularly loss of chromosome 11q, which was identified as a major event degrading the sympathoadrenal phenotypes essential for optimal anti-GD2 responses. Tumors with 11q loss demonstrated increased dedifferentiation, elevated cell-cycle dysregulation, and broader transcriptional plasticity [168]. These changes promote immune escape by reducing the expression of lineage-specific antigens, altering membrane glycosylation patterns, and enabling tumor cells to evade ADCC by diminishing immune receptor engagement. Notably, targeted analysis of patient-matched biopsy pairs before and after multimodality therapy revealed significant clonal evolution, including accumulation of mutations in cell-cycle pathways following treatment. Such evolution suggests that anti-GD2 therapy may apply selective pressure, driving expansion of resistant tumor subclones with reduced GD2 expression or impaired susceptibility to antibody-mediated lysis [167,168]. Beyond genomic instability, other tumor-cell-intrinsic mechanisms also contribute to TIME during anti-GD2 therapy. Dedifferentiated tumor cells often downregulate GD2 biosynthetic enzymes, decreasing surface GD2 density and consequently weakening antibody binding. Reduced GD2 density undermines ADCC and complement activation, contributing to incomplete tumor clearance and relapses. Furthermore, chronically inflamed or therapy-exposed NB cells may alter expression of Fc-receptor ligands or modulate the local cytokine milieu in ways that suppress NK cell function, further limiting antibody-mediated cytotoxicity. These findings parallel the functional NK-cell impairments described in single-cell analyses of NB, in which NK cells displayed reduced cytotoxicity and increased inhibitory receptor signaling in pre- and post-therapy tumors. Such NK-cell dysfunction directly compromises the primary effector arm through which anti-GD2 antibodies exert their antitumor activity [168,169].

An emerging concept supported by the genomic analysis is that NB’s inherent developmental plasticity allows tumor cells to shift between noradrenergic and mesenchymal-like states. Mesenchymal-state NB is characterized by IMT resistance, diminished GD2 expression, and heightened capacity for TIME. Chromosomal rearrangements and therapy-driven DNA-damage responses accelerate these transitions, creating tumor subpopulations that are poorly recognized by GD2-specific immune mechanisms. Identification of cell-cycle pathway mutations accumulating after therapy further underscores the adaptive evolutionary ability of NB under IMT pressure [168]. Altogether, while anti-GD2 therapy remains a cornerstone of HR-NB treatment and has produced survival benefits not achieved by other therapeutic additions, the limitations of this approach stem largely from tumor-intrinsic biology. Chromosomal instability, particularly 11q loss, undermines the developmental phenotypes required for robust ADCC signaling. Tumor dedifferentiation, loss of GD2 expression, and acquisition of therapy-induced mutations collectively weaken antibody efficacy. These insights highlight the need for future therapeutic strategies that preserve or restore pro-inflammatory sympathoadrenal states, enhance NK-cell function, or prevent tumor dedifferentiation during therapy. Addressing these immune-evasive adaptations will be essential to improving the durability of responses to anti-GD2 treatment and reducing relapse in HR-NB [168,169].

### 10.2. CAR-T Cell Therapy in NB

Chimeric antigen receptor (CAR) T-cell therapy has revolutionized treatment outcomes for several hematologic malignancies, including NB (Table 3) yet its translation to NB has been far more limited. The biological and microenvironmental features of NB pose substantial barriers to CAR-T efficacy, and despite promising preclinical activity, clinical trials in pediatric NB have thus far demonstrated only modest and inconsistent responses. Early-phase clinical trials show that CAR-T cell infusion in NB is safe and feasible, but the clinical efficacy remains significantly constrained, highlighting structural challenges inherent to both CAR-T engineering and the NB TME [170,171]. A principal obstacle undermining CAR-T activity in NB is the characteristically immunosuppressive TME, which obstructs both CAR-T infiltration and sustained effector function. NB harbor an environment marked by low immune infiltration, dense stromal barriers, and a dominance of suppressive myeloid populations, all of which diminish CAR-T persistence and reduce anti-tumor potency. This immune-cold phenotype stems from NB’s developmental origins within the NCC, producing tumors that express low levels of MHC class I and limited neoantigens, thereby reducing opportunities for endogenous T_c_ co-activation and cross-priming [78]. The absence of pro-inflammatory signals leads to minimal recruitment of effector lymphocytes, creating a context in which infused CAR-T cells must operate without the cooperative signaling [21,78].

Moreover, NB tumors exert active suppression on infiltrating lymphocytes through the secretion of immunosuppressive cytokines and the recruitment of regulatory cell subsets. Multiple IMTs, including checkpoint inhibitors, have failed to demonstrate robust benefit in NB precisely because the TME suppresses T_c_ activation at multiple levels. CAR-T cells entering this microenvironment encounter high concentrations of TGF-β, IL-10, and soluble inhibitory ligands, together producing functional exhaustion, reduced cytokine secretion, and metabolic paralysis. This reflects a multi-layered barrier in which CAR-T cells cannot sustain the signaling cascades required for serial tumor cell killing [21,56]. Physical and structural obstacles in the NB TME further restrict CAR-T efficacy. NB tumors contain extensive ECM deposition, pericyte and fibroblast-rich stroma, and poorly organized vasculature, all of which hinder CAR-T trafficking to tumor nests. Insufficient infiltration means that even potent CAR constructs fail to reach all tumor sites, especially within BM and metastatic compartments where NB often resides. These barriers are common across pediatric solid tumors but particularly pronounced in NB, reflecting the tumor’s intrinsic stromal architecture and growth pattern [172].

Antigen escape represents an additional challenge limiting CAR-T efficacy. Although GD2 is highly expressed in NB, its expression can be heterogeneous across tumor regions or downregulated under immune pressure, contributing to CAR-T failure. Identifying ideal CAR-T targets in NB has been difficult, with GD2 targeting sometimes insufficient for comprehensive tumor clearance [173]. This challenge has prompted investigations into alternative antigens, multi-antigen CAR constructs, and bispecific CAR-T engineering strategies, but these remain largely preclinical.

CAR-T cell persistence also remains a limiting factor in NB. While in hematologic malignancies, durable CAR-T expansion correlates with durable remission, in NB infused CAR-T cells often exhibit only transient persistence, reducing long-term disease control. The immunosuppressive TME promotes early exhaustion and apoptosis of CAR-T cells, impairing their longevity. This deficiency has motivated efforts to arm CAR-T cells with cytokine-secreting modules, such as IL-15, or to co-express checkpoint-resistant receptors to enhance survival in hostile environments. Even so, these modifications increase the risk of toxicity and must be balanced carefully in pediatric applications [174]. Another important limitation is the potential for severe inflammatory sequelae that must be tightly managed in children with NB. Although CAR-T toxicities in NB have been generally manageable in early trials, cytokine release syndrome and neurotoxicity remain real concerns, particularly when strategies are employed to improve CAR-T expansion or persistence [21,56]. The need for enhanced CAR-T potency in NB thus exists in tension with safety considerations in a vulnerable pediatric population. Despite these challenges, the field continues to evolve toward more resilient CAR-T strategies tailored to the NB TME. Several promising approaches under investigation, including fourth- and fifth-generation “armored” CAR-T cells engineered to secrete cytokines, resist checkpoint inhibition, or degrade ECM barriers. Combinatorial regimens pairing CAR-T cell therapy with anti-GD2 antibodies, oncolytic viruses, checkpoint inhibitors, or metabolic reprogramming agents are also gaining traction, as each addresses a different dimension of the immunosuppressive TME. Nevertheless, the persistent failure of single-agent IMTs in NB underscores that no single modulator is likely sufficient; instead, overcoming the TME will require layered strategies that enable CAR-T infiltration, sustain effector activity, and prevent exhaustion [170,171].

### 10.3. Immune Checkpoint Blockade

#### 10.3.1. TIGIT + PD-L1 Synergy and Its Limitations

Immune checkpoint blockade has transformed therapeutic strategies across a range of adult cancers, yet its translation to pediatric solid tumors such as NB has proven far more challenging. NB is characterized by a profoundly immunosuppressive TME and limited endogenous T_c_ infiltration, conditions that restrict the effectiveness of classical checkpoint inhibitors targeting PD-1 or CTLA-4. Recent high-resolution studies, however, have revealed that NB harbors a distinctive immunoregulatory architecture that is not merely a muted version of adult tumors but instead reflects unique patterns of immune dysfunction [56,175]. Central to this architecture is the NECTIN2–TIGIT axis, identified as a dominant suppressive pathway that regulates both innate and adaptive lymphocyte activity. The discovery that TIGIT blockade synergizes strongly with PD-L1 inhibition in NB provides a mechanistic rationale for combination checkpoint blockade and represents one of the most promising recent directions in NB IMT [176,177]. Sc-RNA-Seq of NB has elucidated the divergent roles of TIGIT and PD-L1 signaling within the TME. NBs are infiltrated by NK cells, T_c_, B_c_, and a wide array of immunosuppressive myeloid populations, but many of these cells display evidence of severe dysfunction. NK cells show significantly reduced cytotoxic signatures, including diminished expression of perforin and granzyme family members, while T_c-_particularly CD8^+^ subsets, exhibit hallmarks of exhaustion such as elevated expression of TIGIT, PD-1, and other inhibitory receptors. This exhausted immunophenotype is more pronounced following CT, highlighting how standard treatment reshapes the immune landscape in ways that reinforce checkpoint-driven immunosuppression. Among these inhibitory circuits, the NECTIN2–TIGIT interaction emerges as a dominant mechanism of immune escape specific to NB [12]. NECTIN2 (CD112) is highly expressed on NB cells and stromal components, while TIGIT is broadly upregulated on NK cells and T_c_ infiltrating NB tissue. Engagement of TIGIT by NECTIN2 blocks activating signals mediated by CD226 and other co-stimulatory receptors, effectively paralyzing the cytotoxic machinery of lymphocytes [178]. This axis plays a more critical role in NB than the PD-1/PD-L1 pathway alone, explaining why single-agent PD-1 or PD-L1 inhibitors have shown minimal clinical success in HR-NB, despite their efficacy in adult malignancies. Importantly, functional analyses in syngeneic mouse models have demonstrated that combined blockade of TIGIT and PD-L1 elicits far stronger antitumor responses than either monotherapy. In vivo experiments showed that inhibition of TIGIT or PD-L1 alone resulted in partial and inconsistent tumor control, whereas dual blockade significantly reduced tumor growth and, in several models, induced complete responses [176,177]. This effect held true even in CT-resistant NB models harboring ALK mutations and MYCN amplification, genetic subtypes notoriously associated with poor prognosis and immune resistance. The synergy appears to arise from restoration of co-stimulatory signaling, rescue of cytotoxic gene expression programs, and increased recruitment and activation of NK and T_c_ within the tumor microenvironment [12]. Despite these promising preclinical findings, several barriers limit the translational potential of TIGIT + PD-L1 combination therapy in clinical NB management. chief among these is the deeply entrenched immune exhaustion phenotype that develops early in tumor evolution and becomes more pronounced following CT. Following cytotoxic treatment, NB tumors exhibit increased infiltration of T_c_ expressing exhaustion markers, including TIGIT, PD-1, and LAG-3, along with metabolic and transcriptional programs characteristic of terminally exhausted cells. This exhaustion phenotype may restrict the proliferative burst and long-term persistence of reinvigorated T_c_ following checkpoint blockade, potentially diminishing the durability of therapeutic benefit [12,177]. Another limitation arises from the highly immunosuppressive myeloid compartment. NB contain populations of myeloid cells exhibiting M2-like phenotypes, pro-angiogenic signaling, metabolic reprogramming, and strong immunoregulatory activity. While TIGIT + PD-L1 blockade enhances lymphocyte function, it does not directly reprogram these myeloid populations, which continue to secrete TGF-β, IL-10, and other suppressive cytokines that dampen immune responses. Without strategies that simultaneously target the myeloid niche, treatment responses to dual checkpoint blockade may be blunted or transient [12,177].

Tumor heterogeneity and plasticity also contribute to limitations in checkpoint inhibitor efficacy. NB exists along an axis between noradrenergic and mesenchymal states, with mesenchymal-like subtypes demonstrating greater IMT resistance and decreased expression of ligands required for effective ADCC or checkpoint-mediated rejuvenation. These tumor states can shift dynamically under therapeutic pressure, enabling immune escape even when checkpoint blockade transiently restores lymphocyte activity [179]. Finally, although dual TIGIT + PD-L1 blockade has produced complete responses in animal models, careful evaluation of toxicity, dosing, and safety profiles is essential for translation into pediatric care. Children with HR-NB are often present with frailty, treatment-induced organ damage, and limited tolerance for additional immunotoxicity. Designing pediatric trials that balance therapeutic intensity with safety will require cautious optimization. Taken together, the findings from single-cell analysis and in vivo checkpoint blockade studies point to TIGIT + PD-L1 co-inhibition as a compelling new direction in NB IMT. Yet the profound immunosuppression inherent to the NB microenvironment, coupled with tumor plasticity and myeloid-driven signaling, underscores that dual blockade is likely necessary but not sufficient on its own [176,177]. Future strategies may require integrated regimens that combine TIGIT + PD-L1 inhibition with agents that remodel the TME, reverse metabolic exhaustion, enhance antigen presentation, or modulate tumor lineage states. As research progresses, the identification of synergistic partners for this checkpoint-based approach will be essential to achieving durable, transformative outcomes for children with HR-NB.

#### 10.3.2. RD3-Centered Immune Checkpoint Blockade

Unlike classical immune checkpoints that primarily modulate lymphocyte signaling at the effector phase, RD3 has emerged as a tumor-intrinsic master regulator of immunogenicity, operating upstream of multiple immune escape mechanisms in HR-NB. Recent evidence demonstrates that therapy-induced RD3 loss orchestrates a coordinated collapse of antitumor immune surveillance by rewiring innate sensing, antigen presentation, checkpoint expression, and CTL engagement. Rather than representing isolated pathways, RD3 governs an interconnected network of immune axes that collectively enforces an immune-silent TME [15,37].

##### RD3–STING Axis: Coupling Innate Immune Sensing to Tumor Immunogenicity

RD3 plays a critical role in maintaining functional innate immune signaling, particularly through preservation of interferon-responsive programs. Loss of RD3 results in attenuation of type I interferon-associated transcriptional outputs and disruption of cGAS-STING-dependent immune activation, thereby impairing danger signaling within NB cells. This dampened innate sensing restricts the recruitment and priming of downstream adaptive immune responses, reinforcing the “cold tumor” phenotype characteristic of therapy-resistant NB [15,37]. Functionally, RD3 deficiency suppresses the immunogenic cell-intrinsic programs required for effective STING-mediated cross-talk between tumor cells and antigen-presenting cells. Restoration of RD3 reinstates inflammatory signaling, enhances interferon-stimulated gene expression, and promotes immune cell infiltration in vivo, establishing RD3 as a permissive switch for productive STING-driven antitumor immunity rather than a redundant accessory pathway [37].

##### RD3–PD-L1 Axis: Transcriptional Control of Immune Checkpoint Dominance

In contrast to checkpoint pathways that are activated reactively by immune pressure, RD3 loss directly drives constitutive upregulation of PD-L1 and CD276 at least at the translational level. RNA-seq analyses reveal that RD3-deficient tumors exhibit heightened expression of immune checkpoint ligands independent of immune infiltration, suggesting that checkpoint dominance in NB is, in part, tumor-programmed rather than immune-induced [15,37]. This RD3–PD-L1 axis is particularly relevant in the post-therapy setting, where cytotoxic treatments accelerate RD3 depletion and simultaneously amplify checkpoint expression. Consequently, checkpoint blockade alone is unlikely to yield durable benefit if the upstream RD3-controlled transcriptional landscape remains uncorrected. RD3 restoration, by contrast, downregulates PD-L1 expression, reshaping the immune inhibitory gradient within the TME and re-sensitizing tumors to immunomodulatory interventions [37].

##### RD3–CTL Axis: Reinstating CTL Function

RD3 loss exerts profound effects on CTL engagement by disrupting both immune recognition and effector execution. RD3-deficient tumors display reduced CD8^+^ T-cell infiltration, impaired cytokine release, and diminished cytolytic gene signatures, reflecting a failure of productive immunoediting. Importantly, this dysfunction is not solely attributable to checkpoint signaling but arises from compounded defects in antigen presentation, co-stimulation, and inflammatory conditioning [15]. In vivo models demonstrate that reinstating RD3 restores CTL recruitment and functional activation, leading to enhanced tumor clearance and durable immune surveillance. RD3 thereby operates as a gatekeeper of effective CTL engagement, linking tumor cell identity with adaptive immune execution rather than serving as a passive differentiation marker [37].

##### RD3-Antigen Presentation Axis: Preserving Tumor Visibility

Among the most consequential effects of RD3 loss is its disruption of antigen presentation machinery. RD3-deficient NB cells exhibit marked downregulation of MHC class I and II components, including β2-microglobulin, effectively rendering tumor cells invisible to both CD8^+^ and CD4^+^ T_c_. This antigen presentation collapse represents a dominant mechanism of immune escape that precedes and potentiates checkpoint-mediated suppression [15]. Mechanistically, RD3 governs transcriptional programs that preserve lineage fidelity and epithelial differentiation, states intrinsically linked to intact antigen processing pathways. Restoration of RD3 reverses antigen presentation deficits, re-establishes immune visibility, and cooperates with reinvigorated innate and adaptive immune responses to enforce tumor control [37].

Collectively, RD3 defines a central immunoregulatory hub that integrates innate sensing, checkpoint regulation, antigen presentation, and CTL function into a single tumor-intrinsic axis of immune competence. Unlike surface checkpoint receptors, RD3 operates upstream of immune engagement, dictating whether tumors can be recognized and eliminated in the first place. The convergence of RD3–STING, RD3–PD-L1, RD3–CTL, and RD3–antigen presentation axes underscores why isolated checkpoint blockade has failed in HR-NB and why RD3-targeted restoration strategies may be necessary to unlock the full therapeutic potential of IMT [15,37]. As such, RD3 is best conceptualized not as a biomarker, but as a tumor-intrinsic immune checkpoint regulator, one whose modulation may convert immunologically silent NB into tumors capable of sustaining durable, multi-arm antitumor immunity.

### 10.4. Exosome-Based IMT Approaches: Engineering NK-Cell-Derived Exosomes

Among emerging strategies designed to overcome the profound immune suppression characteristic of NB, exosome-based IMTs have gained significant attention. In NB, TDEs have been firmly established as drivers of immunosuppressive reprogramming, undermining both innate and adaptive immune responses. However, their immunomodulatory capacity has also opened the possibility of repurposing exosomes as therapeutic tools, particularly through engineering exosomes derived from NK cells to restore antitumor immunity and counteract NB’s immune-evasive tactics [142,143,151]. The study on exosomes and immune modulation in NB highlights that TDEs profoundly impair NK-cell and T-cell function, inhibit dendritic-cell maturation, and promote the expansion of immunosuppressive myeloid cells. Such effects create a TME resistant to conventional IMTs and checkpoint blockade. However, the therapeutic potential of NK-cell-derived exosomes has been emphasized, which can be engineered to deliver immune-activating molecules or checkpoint inhibitors, thereby reversing many of these suppressive effects. This duality, where exosomes serve as both a mechanism of tumor escape and a tool for therapeutic intervention, marks them as uniquely promising in the context of NB [45,142]. NK-cell-derived exosomes (NK-Exos) possess intrinsic cytotoxic properties that mirror the functional profile of their parent NK cells. They carry perforin, granzymes, and other cytolytic proteins capable of inducing apoptosis in tumor cells. Because exosomes are nano-sized and capable of penetrating dense extracellular matrices, they can access tumor regions often unreachable by NK cells themselves, particularly relevant in NB, where extensive stromal barriers limit immune infiltration. NK-Exos not only retain but, in some contexts, enhance NK-cell cytotoxic behavior by delivering activating cues that counteract TDE-mediated suppression. In models of NB and similar tumors, NK-Exos have been shown to restore NK-cell function and partially overcome the metabolic and cytokine-mediated suppression imposed by the NB microenvironment [150,151]. The therapeutic potential of engineered NK-Exos is particularly striking. Because exosomes can be modified ex vivo, researchers can load them with immune-activating molecules, such as cytokines, microRNAs, or inhibitors of immune checkpoints, thereby transforming them into highly targeted biologic delivery systems for instance, engineering NK-Exos to carry inhibitors of molecules like TIGIT or PD-L1. Such exosomes could function dually: directly killing tumor cells and simultaneously reactivating endogenous NK and T_c_ by interfering with dominant suppressive checkpoints within the TME. This approach is attractive because it circumvents the barriers that have historically limited checkpoint-blocking antibodies in NB, such as poor lymphocyte infiltration and weak baseline inflammation [150,151]. Exosomes, unlike full-size immune cells or large antibodies, can distribute throughout the tumor mass efficiently, and when engineered with checkpoint-blocking cargo, they may enable sustained local modulation of immune signaling [154].

Engineered NK-Exos also hold promise in addressing NB’s frequent downregulation of antigen-presentation machinery and its reliance on non-immunogenic states. Because NK-cell cytotoxicity is not dependent on antigen presentation but instead on a balance of activating and inhibitory ligands, NK-Exos bypass one of the major barriers facing T-cell-based IMTs. Furthermore, exosomes can deliver microRNAs known to modulate tumor-cell immune phenotypes. By selectively transporting microRNAs that target NB-specific immune-escape genes or metabolic regulators, engineered NK-Exos could recalibrate tumor-cell signaling to enhance susceptibility to immune attack. Although NK-Exos’ capacity to carry checkpoint inhibitors and immune-activating molecules are focused, the vesicle biology strongly supports the feasibility of miRNA-based engineering [180,181]. An additional therapeutic advantage of NK-Exos lies in their potential to counteract TDE-mediated suppression of NK cells. TDEs from NB inhibit NK-cell activation and proliferation while reprogramming immune cells toward dysfunction. NK-Exos could competitively displace TDEs from key signaling niches or interfere with their uptake by immune cells, mitigating the suppressive influence of tumor-derived vesicles. In this way, NK-Exos may not only deliver pro-immune payloads but also help restore baseline immune competence by blocking or neutralizing TDE functions. This concept parallels other vesicle-based antagonism strategies explored across cancers, where engineered exosomes are used to “re-educate” immune cells that have been rewired by tumor-derived signals [150,151].

Still, despite the promise of NK-Exos, significant challenges remain. The immunosuppressive NB TME features abundant myeloid cells, high concentrations of inhibitory cytokines such as TGF-β, and extensive metabolic competition. These factors may limit the functional longevity or potency of NK-Exos after delivery. Moreover, exosome biodistribution, systemic clearance, and ideal routes of administration are active areas of investigation. Studies have underscored that blockade of TDE biogenesis or uptake enhances the efficacy of IMT, suggesting that NK-Exo-based approaches may need to be combined with TDE inhibition to achieve optimal benefit [182]. In conclusion, engineered NK-cell-derived exosomes represent a transformative opportunity for NB IMT. By delivering cytotoxic proteins and immune-activating signals and potentially carrying checkpoint-blocking molecules such as anti-TIGIT or anti-PD-L1, NK-Exos could overcome several of the obstacles that limit T-cell-based and antibody-based IMT. Their ability to penetrate the dense NB tumor architecture, resist suppressive signaling, and recalibrate immune interactions positions them as versatile tools for next-generation treatment strategies [141,145,181]. Although further preclinical development and clinical validation are necessary, the mechanistic insights provided strongly support NK-Exos as a powerful and adaptable platform for enhancing antitumor immunity in HR-NB.

### 10.5. Cytokine Therapy

Cytokine therapy has been widely explored as a strategy to reverse the strongly immunosuppressive TME in NB, which is typically characterized by low immunogenicity, reduced MHC class I expression, and infiltration of suppressive myeloid populations such as TAMs and MDSCs, along with inhibitory cytokines like TGF-β that dampen T- and NK-cell activity. Historically, interleukin-2 (IL-2) has been incorporated into standard therapy in combination with anti-GD2 antibodies and GM-CSF, where it enhances NK-cell activation and antibody-dependent cellular cytotoxicity; however, its clinical benefit is limited by toxicity and its paradoxical expansion of TREGs, which can sustain immunosuppression. This has led to the development of next-generation cytokine approaches, particularly IL-15, which preferentially expands NK cells and memory CD8^+^ T_c_ without promoting TREGs, thereby more effectively shifting the immune balance toward tumor clearance; preclinical studies show that IL-15 combined with anti-GD2 therapy induces stronger tumor regression than IL-2-based regimens. Other cytokines such as IL-12 further contribute by promoting Th1 polarization, increasing IFN-γ production, and enhancing antigen presentation, thereby converting the TME from “cold” to inflamed. More recently, engineered cytokines and immunocytokines, including antibody-cytokine fusion proteins and IL-2 variants biased toward effector cells, have been designed to improve tumor targeting while reducing systemic toxicity. Importantly, cytokines are increasingly used in combination strategies, including with anti-GD2 antibodies, CAR-T or CAR-NK cells (often engineered to express IL-15 for persistence), checkpoint inhibitors, and TGF-β blockade, to overcome multiple layers of immune suppression simultaneously. Overall, while classical cytokine therapy demonstrated proof of concept, current approaches emphasize targeted delivery, reduced toxicity, and rational combinations to reprogram the NB TME and achieve sustained anti-tumor immunity. However, current cytokine therapy in NB faces several real-world limitations, including significant systemic toxicity and off-target effects, especially with agents like IL-2, which restrict the dose that can be safely administered. In addition, some cytokines paradoxically expand immunosuppressive populations such as TREGs, thereby undermining the intended anti-tumor immune response. Furthermore, the short half-life of cytokines and their poor tumor-specific targeting lead to inadequate persistence in the TME, resulting in incomplete and often transient reversal of immune suppression.

## 11. Emerging Therapeutic Strategies to Overcome Immune Evasion in NB

**Figure 8:**
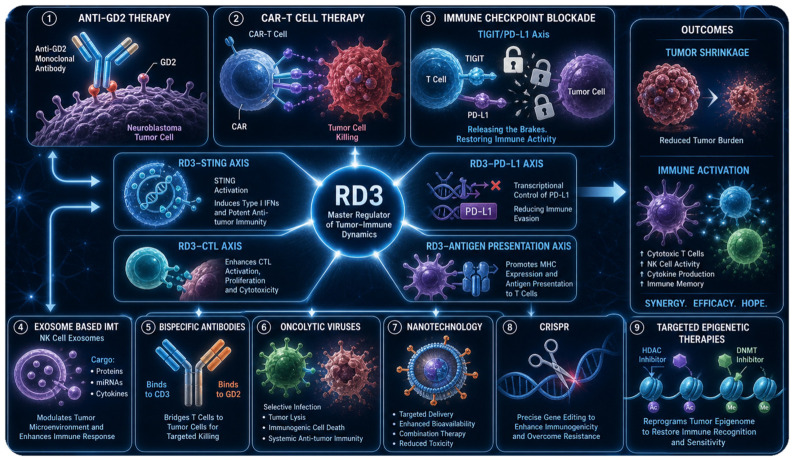
***Current and evolving IMT strategies for NB.*** Established and emerging therapeutic modalities integrated with RD3-mediated immune modulation pathways, highlighting their synergistic potential to enhance immune visibility and improve NB clinical outcomes.

NB remains one of the most challenging pediatric cancers to treat, especially in its HR forms, where deeply embedded mechanisms of immune evasion limit the effectiveness of conventional and IMT interventions. As understanding of the molecular, metabolic, and immunologic determinants of NB pathogenesis has advanced, attention has increasingly shifted toward next-generation therapeutic strategies capable of counteracting these escape pathways. Recent studies provide detailed insight into how novel platforms, including bispecific antibodies, oncolytic virotherapy, nanotechnology-driven delivery systems, CRISPR-mediated genomic editing, and targeted epigenetic therapies, may be leveraged to overcome the immune resistance that defines the NB TME (Figure 8) [183]. These emerging modalities share a common theme: they are designed not only to kill tumor cells directly but also to reshape the immune landscape, reverse immune dysfunction, and sensitize NB to additional modes of therapy. In doing so, they represent a paradigm shift away from therapies that attack the tumor in isolation and toward strategies that dismantle the molecular and immunologic networks enabling tumor survival.

### 11.1. Bispecific Antibodies

Bispecific antibodies (BsAbs) have emerged as a powerful tool to enhance tumor–immune cell interactions by physically bridging immune effector cells, most commonly T_c_ or NK cells, to tumor antigens. Their therapeutic potential of BsAbs highlighted as a class of “novel and emerging IMTs” capable of restoring immune control even in cancers with strong immunosuppressive signals [184]. In NB, BsAbs targeting antigens such as GD2, B7-H3, or ALK can engage T_c_ directly with tumor cells, effectively bypassing the suppressed antigen-presentation pathways that limit classical T_c_ activation. By using CD3-engaging formats, BsAbs can redirect polyclonal T_c_ responses against NB cells regardless of TCR specificity. This mechanism is particularly relevant given NB’s low neo-antigen load and weak immunogenicity, features that contribute significantly to immune escape. The ability of BsAbs to force immune synapse formation may therefore help counteract T_c_ exclusion and exhaustion that are hallmarks of NB tumors [184]. Moreover, in tumors where NK-cell activity is compromised, BsAbs engineered to recruit NK cells, including Fc-optimized or NKp46-engaging formats, may restore cytotoxicity lost due to the suppressive TME. Bispecific antibodies can function effectively even in hostile microenvironments, provided they are paired with strategies that mitigate inhibitory checkpoint signals [184]. As NB research advances, next-generation BsAbs incorporating enhanced affinity, dual-antigen targeting, or combined checkpoint blockade are expected to play an increasingly central role in treatment.

### 11.2. Oncolytic Viruses

Oncolytic virotherapy has evolved beyond its origins as a simple cytolytic tool and is now recognized as a potent method for reprogramming the immune microenvironment. Oncolytic viruses (OVs) selectively infect and lyse tumor cells, releasing tumor antigens, inducing local inflammation, and recruiting immune effector populations that are typically absent in immune-cold tumors such as NB [185,186]. OVs are one of the “novel immunotherapeutic approaches” showing potential to overcome the immunosuppressive constraints that limit current therapies. In NB, OVs may provide several advantages. First, viral infection can upregulate antigen-processing machinery and stimulate type I interferon responses, counteracting the defects in antigen presentation that NB exploit. Second, viral infection may modulate the phenotype of TAMS and MDSCs, tipping the balance toward a more pro-inflammatory state. Third, OVs can serve as delivery platforms carrying cytokines (e.g., IL-12, GM-CSF), co-stimulatory ligands, or even checkpoint inhibitors directly into the TME [185,187]. Given the profound NK-cell dysfunction and T-cell exhaustion in NB, OVs may also act synergistically with IMTs such as anti-GD2 antibodies or CAR-T cells by improving immune infiltration and reducing stromal barriers [187,188]. The biological insights suggest that oncolytic virotherapy is a promising strategy for awakening NB’s dormant antitumor immunity and generating immune memory that reduces relapse risk.

### 11.3. Nanotechnology-Driven Approaches

Nanotechnology has gained traction as a versatile tool to modulate immune responses and deliver therapeutic payloads with high specificity. Nanotechnology among the “novel and emerging therapeutic strategies” with potential relevance across immune-evasive tumors. In NB, nanocarriers can overcome several treatment barriers, including drug efflux, stromal exclusion, and off-target toxicity [189]. Nanoparticle-based platforms offer several distinct immunologic advantages. They can be engineered to deliver: immunomodulators that reverse local immune suppression (e.g., TGF-β inhibitors, STAT3 inhibitors); neoantigen-enriched tumor lysates to dendritic cells, promoting enhanced antigen presentation; and siRNA-based therapies targeting immune-escape genes, such as those regulating PD-L1 expression or metabolic rewiring enzymes. Importantly, nanoparticles can be tailored to penetrate the dense ECM of NB, enhance tumor uptake, and sustain release of therapeutic compounds [190,191]. They may also serve as carriers for CRISPR components or epigenetic drugs, directly linking nanotechnology with other emerging therapeutic modalities. The adaptability of these constructs allows them to simultaneously disrupt tumor-intrinsic signaling, reprogram the TME, and deliver synergistic agents such as checkpoint inhibitors or oncolytic viruses [191,192]. Thus, nanotechnology holds potential not merely as a delivery system but as a multi-functional IMT approach capable of simultaneously targeting several dimensions of NB immune escape.

#### 11.3.1. CRISPR-Based Therapeutic Strategies

While IMTs aim to correct immune dysfunction, CRISPR-based strategies aim to correct the genetic and transcriptional programs driving NB progression and immune evasion. Studies have identified CRISPR-Cas9 as a cutting-edge tool with potential for both research and therapeutic applications in NB [193]. CRISPR systems can be used to: knockout immune-suppressive genes (e.g., TGF-β receptors, checkpoint ligands); target oncogenic drivers such as MYCN, ALK, or downstream components of PI3K/AKT/mTOR and RAS–MAPK pathways; restore expression of antigen-processing machinery or NK-activating ligands; and investigate gene regulatory circuits that give rise to mesenchymal or dedifferentiated NB phenotypes associated with immune escape [193,194]. Because NB cells often display epigenetically mediated plasticity, CRISPR-based interventions allow direct interrogation and reversal of lineage transitions that impair immunogenicity. For example, disrupting enhancers supporting mesenchymal identity may re-sensitize tumor cells to T-cell and NK-cell recognition, restoring pathways that anti-GD2, checkpoint inhibitors, or CAR-T cells can exploit [195].

In preclinical models, CRISPR has already been used to identify novel tumor surface antigens, understand treatment-induced resistance, and propose new targets for CAR-T cell engineering. CRISPR is poised to significantly accelerate precision medicine approaches for NB, particularly when integrated with multi-omic profiling [194,195].

#### 11.3.2. Targeted Epigenetic Therapies

Epigenetic dysregulation lies at the core of NB’s cellular plasticity, lineage switching, metabolic rewiring, and immune resistance. Growing portfolio of epigenetic regulators being explored as therapeutic targets in NB, driven by the realization that epigenetic reprogramming influences both tumor progression and immune responsiveness [105,128,129]. Targeted epigenetic therapies, including inhibitors of HDACs, DNMTs, BET-bromodomain proteins, histone demethylases, and other chromatin remodelers, offer several immune-relevant benefits: (i) restoring antigen presentation, increasing MHC-I and NK-activating ligand expression; (ii) reversing TME suppression by modulating cytokine expression and reducing immunosuppressive myeloid recruitment; (iii) counteracting therapy-induced tumor dedifferentiation, preserving the noradrenergic identity associated with improved IMT outcomes; and (iv) enhancing susceptibility to checkpoint blockade, particularly TIGIT- or PD-L1-directed therapies [31,64,121,134,162]. Furthermore, epigenetic approaches may work synergistically with CRISPR and nanotechnology. Epigenetic sensitization can render tumor cells more responsive to immune attack, while CRISPR can identify optimal epigenetic nodes for intervention [193,194]. Nanoparticles can then deliver epigenetic drugs or editing tools directly into the tumor [189].

Collectively, targeted epigenetic therapies represent a promising category of “immunogenic modulators” capable of reversing NB’s immune-cold state.

In conclusion, the ongoing evolution of NB-IMT reflects a shift toward multimodal strategies that address the layered complexity of TIME. Bispecific antibodies improve the physical and functional engagement between immune cells and tumor cells. OVs can inflame the TME and restore antigenicity. Nanotechnology provides a versatile platform for delivering immunomodulatory payloads directly into tumor niches. CRISPR enables precise targeting of oncogenic or immune-suppressive circuits, while targeted epigenetic therapies reverse tumor plasticity and enhance immune susceptibility [184,189,193]. Together, these emerging approaches represent a coherent effort to dismantle NB-TIME architecture at multiple levels. Integrating these technologies into rational combination therapies will be essential for unlocking durable, transformative responses in children with HR-NB.

## 12. Integration of Multi-Omics and Single-Cell Technologies in Understanding Immune Escape

The emergence of high-resolution multi-omics and single-cell technologies has transformed our understanding of NB biology, particularly the mechanisms by which NB tumors evade immune surveillance. Traditional bulk sequencing approaches have long provided valuable insights into driver mutations, copy-number alterations, and transcriptional states associated with NB progression, but they have obscured the profound intratumoral heterogeneity and cellular diversity that shape immune interactions [196]. By deconvoluting tumors at cellular and molecular resolutions, scRNA-seq, combined with complementary multi-omics platforms, has revealed an intricate immunoregulatory ecosystem that cannot be appreciated through bulk methods alone [197]. These technologies have uncovered immune-escape mechanisms embedded within tumor-cell developmental programs, treatment-induced cell-state transitions, and dynamic cross-talk between tumor, immune, and stromal compartments [12,49]. In doing so, they provide a framework for therapeutic strategies tailored to the complex interplay of genetic, epigenetic, metabolic, and immunologic factors that define NB immune resistance. The integration of these high-content modalities aligns with observations from large-scale studies of cancer immune evasion emphasizes the necessity of multi-omics approaches for understanding tumor immune escape and designing rational IMT. Dissecting tumor–immune interactions require technologies that account for tumor heterogeneity, microenvironmental influences, metabolic reprogramming, and cross-lineage signaling, all of which can only be captured through combined genomic, transcriptomic, proteomic, and epigenomic interrogation [196,197]. In NB specifically, scRNA-seq efforts have validated this point by revealing multiple layers of dysfunction across immune and tumor compartments, offering direct mechanistic insight that is now guiding new IMT design.

### 12.1. Insights from Single-Cell RNA-Sequencing Studies

The most comprehensive single-cell study of NB profiled tumors, including matched pre- and post-CT samples, demonstrating that NB tumors contain a rich diversity of immune and stromal cells, comprising at least 17 distinct immune subsets, including NK cells, B cells, multiple T_c_ populations, dendritic cells, monocytes, and four macrophage subsets. Critically, NK and T_c_ in NB are not simply reduced in number but exhibit deeply impaired functional states, with NK cells showing reduced cytotoxicity and T_c_ showing pronounced exhaustion markers. The cytotoxic genes required for effective tumor clearance, such as *GZMB*, *PRF1*, and *GNLY*, were markedly downregulated, particularly in NK cells, while T_c_ exhaustion markers, including PD-1, *LAG3*, *TIGIT*, and *CTLA4*, were elevated. These alterations converge into profound immune suppression, highlighting that NB tumors orchestrate multiple, overlapping mechanisms of lymphocyte dysfunction [12,49]. Furthermore, CT profoundly reconfigures the immune landscape, exacerbating rather than alleviating immune exhaustion. Post-treatment samples contained even higher frequencies of dysfunctional T_c_ with elevated inhibitory-receptor expression and impaired cytotoxic functions. NK-cell dysfunction also intensified following therapy due to disrupted signaling and imbalanced expression of activating versus inhibitory receptors. These findings underscore those cytotoxic therapies, while essential for tumor debulking, may inadvertently reinforce immune-escape mechanisms by fostering durable exhaustion states and altering cellular interactions within the microenvironment [49]. Another key insight derived from scRNA-seq concerns the central role of the NECTIN2–TIGIT checkpoint axis in NB. By mapping ligand–receptor interactions across tumor and immune compartments, the study identified NECTIN2 expression on tumor and stromal cells as a dominant suppressive signal engaging TIGIT on T and NK cells. Functional experiments confirmed that combined TIGIT + PD-L1 blockade significantly restored lymphocyte activity and produced complete tumor regressions in vivo. This finding not only underscores the value of sc-ligand–receptor interactome mapping but also demonstrates how such approaches uncover NB-specific immune-evasion pathways not observed in other pediatric tumors [12]. sc-profiling also illuminated the plasticity of NB tumor cells, revealing transitions between noradrenergic and mesenchymal states. Mesenchymal-like tumor cells exhibited greater IMT resistance, reduced antigenicity, and deeper engagement with immunosuppressive microenvironment signals. These developmental trajectories were further shaped by genetic alterations, such as MYCN amplification or ALK mutations, and by treatment pressures such as CT, which enriched for more resistant cell states. This cellular plasticity directly influences immune escape and treatment outcomes, demonstrating the importance of integrating single-cell transcriptomics with clinical and molecular data [49,198].

### 12.2. Multi-Omics Approaches Shaping Future IMT

Immune-escape mechanisms stem from the intersection of tumor-intrinsic factors, including metabolic reprogramming, epigenetic alterations, and genetic drivers, and tumor-extrinsic components such as immune cells, stromal architecture, vasculature, and microbiome cues. Multi-omics approaches are uniquely suited to chart these interconnected networks. Integrating genomic, transcriptomic, proteomic, epigenomic, and metabolomic data provides a systems-level view of how NB engages immune-evasive strategies and which molecular nodes may be most effectively targeted. This multi-layered perspective advances the identification of novel therapeutic interventions, including metabolic modulators, epigenetic therapies, bispecific antibodies, engineered cell therapies, oncolytic viruses, and nanotechnology-based IMT delivery systems [12,49,196]. Multi-omics analyses are particularly valuable in understanding NB immune-metabolic coupling, the fundamental niche to cancer immune escape. By integrating metabolic and immune profiling, one can characterize how nutrient competition, hypoxia, and metabolite accumulation impair NK- and T-cell function within NB tumors. Identifying metabolic bottlenecks using proteomics and metabolomics can pinpoint therapeutic interventions that restore immune cell activity, such as metabolic reprogramming agents or inhibitors of tumor-driven metabolic pathways [81,196,197]. Epigenetic multi-omics approaches, combining ATAC-seq, DNA methylation profiling, and ChIP-seq, have also revealed how NB tumors rewire chromatin landscapes through super-enhancers, MYCN-regulated transcriptional networks, and treatment-induced epigenetic remodeling. These data are crucial for developing epigenetic therapies that reverse immune-evasive states by restoring antigen-presentation machinery or activating pro-inflammatory transcriptional programs. The integration of epigenomic and transcriptomic data is key to uncovering epigenetic checkpoints that shape immune resistance, thus guiding rational combinations of IMTs with epigenetic drugs [199]. Proteomics and phospho-proteomics offer yet another layer of insight, especially regarding immune-checkpoint regulation, kinase signaling cascades, and stromal–immune interactions. By connecting protein-level signaling to transcriptomic states, researchers can identify post-transcriptional regulatory mechanisms critical for immune escape, such as modulation of immune ligands, secretion of immunosuppressive cytokines, and kinase-driven exhaustion pathways. Finally, multi-omics integration is playing an increasingly important role in stratifying patients for tailored IMT [52,93,130]. By combining genomic drivers (e.g., MYCN, ALK) with immune-phenotypic signatures derived from scRNA-seq and proteomics, it is possible to identify which patients are most likely to respond to checkpoint inhibitors, CAR-T cell therapy, NK-cell-based treatments, bispecific antibodies, or combination regimens [12,156,171]. This integrated, precision-IMT framework approaches are indispensable for advancing NB therapeutics beyond incremental improvements and toward durable, transformative clinical outcomes for HR-NB.

## 13. Limitations

The authors acknowledge that this review is limited by the evolving and incomplete nature of current knowledge on NB immune evasion, with many mechanistic insights derived from preclinical models and not yet fully validated in large, well-annotated patient cohorts. The relative contributions and interplay of tumor-intrinsic programs, including lineage plasticity, epigenetic reprogramming, metabolic adaptation, and RD3-associated signaling, remain incompletely defined in clinical contexts. Additionally, the marked heterogeneity and dynamic cell-state transitions in NB complicate patient stratification and limit the translation of emerging immunotherapeutic strategies. Finally, despite advances in multi-omics profiling, their integration into biomarker-driven clinical trials and real-time therapeutic decision-making remains limited, underscoring the need for longitudinal, spatially resolved, and functionally validated studies.

## 14. Conclusions

NB embodies one of the most complex and immunologically evasive malignancies of childhood. Its biology is shaped by the interplay of developmental lineage, epigenetic plasticity, metabolic adaptation, and selective pressures exerted by therapy. These forces create a tumor ecosystem capable of resisting both endogenous immune surveillance and modern immunotherapeutic strategies. Here, we synthesize several unifying themes that highlight not only the challenges inherent in treating NB but also the opportunities for advancing therapeutic approaches based on deeper biological understanding. While an earlier review [8] provided an important foundation by outlining the major components of the NB TME, particularly immune suppression driven by myeloid cells, checkpoint pathways, and limited T/NK-cell activity, our current review substantially advances the field by incorporating recent multi-omics, single-cell, and mechanistic insights that redefine immune evasion as a dynamic, programmable process rather than a static barrier. Beyond the descriptive TME components and emerging IMTs (e.g., anti-GD2, checkpoint blockade), the present review integrates tumor-intrinsic determinants (e.g., RD3 axis, lineage plasticity, epigenetic rewiring) with extrinsic immune suppression, providing a systems-level framework linking cellular identity to immune visibility. In addition, this review highlights newly recognized mechanisms not emphasized in 2020, including exosome-mediated immune suppression, ferroptosis and metabolic–immune coupling, therapy-induced immune remodeling, and spatial immune heterogeneity revealed by single-cell analyses. Importantly, we move beyond cataloging targets to emphasize network-based and combinatorial strategies, framing NB immune escape as a multi-layered, adaptable architecture that requires integrated therapeutic disruption rather than single-axis targeting. Thus, the novelty lies in shifting from a “component-based” view to a mechanistically integrated and evolution-aware model of TIME in NB.

A central challenge arises from the deeply immunosuppressive TME that characterizes NB. Studies revealed severely impaired antitumor lymphocyte function, including markedly reduced cytotoxicity in NK cells and widespread exhaustion among T-cell subsets, underscoring the necessity of therapies capable of reinvigorating lymphocyte function despite both tumor-intrinsic suppression and stromal-derived inhibitory signals. Studies have emphasized the complexity of such suppressive networks, stressing that tumors engage metabolic, epigenetic, and signaling pathways simultaneously to blunt immune activity and create niches resistant to immune clearance. In NB, similar interdependencies are evident, as immune dysfunction cannot be understood without recognizing the coordinated influence of oncogenic drivers, stromal architecture, myeloid-rich infiltrates, and metabolic stressors.

Another major barrier is the profound cellular plasticity observed in NB, which contributes not only to tumor heterogeneity and therapy resistance but also directly to immune escape. This plasticity involves transitions between noradrenergic and mesenchymal phenotypes, each carrying distinct immunologic characteristics. Mesenchymal-like states are strongly associated with immune evasion, reduced antigenicity, and poorer therapeutic response. The dynamic reversibility of these states, driven in part by epigenetic mechanisms, raises important questions about whether lineage reprogramming therapies can restore immunogenicity and enhance susceptibility to IMT. NB epigenome studies highlighted how chromatin regulators, super-enhancers, and transcriptional circuitry shape these lineage identities and are themselves powerful modulators of immune visibility and tumor–immune interactions. Yet significant gaps remain in understanding which epigenetic regulators are most responsible for immune suppression and how targeted reprogramming might alter downstream immune dynamics.

Therapy-induced immune remodeling represents another critical dimension of NB biology. CT, though indispensable, reshapes the immune landscape in ways that may exacerbate exhaustion, promote myeloid-cell expansion, and accelerate dedifferentiation. This treatment-driven remodeling underscores why many patients relapse despite initial response and why IMT introduced after induction therapy often fail to achieve durable efficacy. Multi-omics integration will be essential for mapping these transitions at high resolution and determining how to intervene at the molecular nodes responsible for post-therapy immune collapse.

Despite these formidable challenges, the prospects for overcoming NB immune evasion are rapidly expanding. Advances in immune checkpoint blockade, particularly RD3–PD-L1, RD3–STING, RD3–NFκB, Nectin2–TIGIT axes inhibition, demonstrate that targeted disruption of dominant suppressive pathways can produce complete tumor regressions in preclinical models. However, the success of combination checkpoint blockade rests on addressing additional layers of immune resistance, such as myeloid-cell–driven suppression and stromal barriers. The need for combinatorial approaches, integrating checkpoint blockade, NK-cell based therapies, CAR-T cell enhancements, epigenetic therapies, metabolic reprogramming, and novel delivery platforms, is repeatedly emphasized. Effective immune intervention in complex tumor ecosystems requires simultaneous inhibition of multiple escape mechanisms, tailored to the tumor’s specific molecular and immunologic profile.

Moreover, the rise of personalized IMT guided by genomic, epigenomic, transcriptomic, and sc-profiles, offers a viable roadmap for improving outcomes. Precision medicine approaches will enable clinicians to match patients with therapies suited to their tumor’s signaling networks, developmental state, immune infiltrate composition, and surface antigen landscape. This strategy highlights that future breakthroughs will arise from integrating mechanistic insights into both tumor biology and host immunity.

Looking forward, several unanswered questions will shape the trajectory of NB IMT research.

How can lineage plasticity be therapeutically stabilized without compromising normal development?

Which epigenetic regulators represent the most effective targets for immune reactivation?

How can oncolytic viruses, bispecific antibodies, engineered NK-cell-derived exosomes, and next-generation CAR-T cells be optimized to overcome NB-specific immune barriers?

How can multi-omics integration be operationalized in real-time clinical decision-making for children with NB?

The answers to these questions will determine whether current advances translate into durable cures. Despite the complexity of NB immune evasion, the convergence of sc-analytics, multi-omics profiling, rational combinatorial design, and mechanistically informed IMTs marks a turning point for the field. By embracing the biological intricacies revealed through cutting-edge research, the next generation of therapies may finally dismantle the immune barriers that have long protected NB from complete eradication.

## Figures and Tables

**Table 1:** Immune Checkpoints and Cell Cycle–Associated Mechanisms of Immune Evasion in NB.

Checkpoint/Pathway	Type	Mechanism in NB	Cell-Cycle/ Tumor-Intrinsic Link	Ref.
PD-1/PD-L1 axis	Adaptive immune checkpoint	PD-L1 expression inhibits T-cell activation via PD-1 signaling, leading to T-cell exhaustion and immune escape	PD-L1 upregulation induced by inflammatory signaling; MYCN-driven tumor programs & cytokines from tumor cells/macrophages regulate PD-L1	[16,17]
CTLA-4 (B7–CTLA-4)	Adaptive immune checkpoint	Competes with CD28 for B7 ligands → suppresses T-cell priming in lymphoid tissues	Functions at early activation stage; often combined with PD-1 blockade to overcome adaptive resistance	[17,18]
TIGIT–NECTIN2 axis	Adaptive/NK checkpoint	TIGIT signaling suppresses T_C_ and NK cell cytotoxicity; inhibitory axis in NB TME	Associated with dysfunctional immune infiltrates; cooperates with PD-L1 signaling; linked to tumor evolution under therapy	[12,19]
CD47–SIRPα (“don’t eat me” signal)	Innate immune checkpoint	Tumor-expressed CD47 inhibits macrophage phagocytosis via SIRPα	Upregulated in NB; supports survival and resistance independent of adaptive immunity	[20]
B7-H3 (CD276)	Emerging checkpoint	Overexpressed in NB; inhibits T_C_/NK cell activity and drives TIME	Often co-targeted with GD2; linked with aggressive tumor phenotype	[21]
LAG-3, TIM-3 (co-inhibitory receptors)	Adaptive exhaustion markers	Co-expression with PD-1 on exhausted T_c_ reduces effector function	Reflects chronic antigen exposure and tumor-driven immune exhaustion	[22]
MHC-I downregulation	Antigen presentation checkpoint (indirect)	Reduced HLA class I expression limits T_C_ recognition	Linked to low mutational burden and transcriptional dysregulation	[23]
MYCN-driven immunosuppressive network	Tumor-intrinsic regulator	MYCN promotes Th2/M2 skewing, suppress NK/T_C_ responses, and remodels immune microenvironment	Directly regulates cell-cycle progression, metabolism, and transcription; couples proliferation with TIME	[24]
Tumor-associated macrophage (TAM) PD-L1 regulation	Microenvironment checkpoint	Myeloid cells express PD-L1, suppress T_C_ even when tumor PD-L1 is low	MYCN tumor signaling drives cytokine secretion (e.g., MIF) → induces PD-L1 on macrophages	[16]
CDK1 (cell-cycle kinase)	Cell-cycle–immune interface	High CDK1 ⟶ poor immune infiltration and reduced cytokine; inhibit-ion induces ICD	Central G2/M regulator; inhibition releases DAMPs (CRT, HMGB1) enhancing antitumor immunity	[25]
CDK7/CDK9 transcriptional regulators	Cell-cycle checkpoint regulators	Control transcription of cell-cycle and immune-related genes; influence stress responses and checkpoint activation	Integrate DNA damage response (ATM/ATR → CHK1/2) with transcriptional programs affecting immune signaling	[26]
DNA damage response (ATM/ATR–CHK axis)	Cell-cycle checkpoint (G1/S, G2/M)	DDR alters tumor immuneogenicity and cytokine signaling; enhance immune recognition	Core cell-cycle checkpoints modulate antigen presentation and immune signaling pathways	[27]
TME immune-suppression (TAMs, CAFs, MDSCs)	Indirect checkpoint network	Barriers, cytokines, metabolism suppress immune infiltration and activation	Tumor lineage plasticity and therapy-induced changes (ADR → MES transition)	[19]
GD2-associated immune modulation	Tumor antigen–linked immune targeting	GD2-targeted therapies enhance ADCC, require overcoming checkpoint-mediated suppression	Often combined with checkpoint inhibitors (e.g., B7-H3, macrophage checkpoints)	[28]

**Table 2:** Summary of Epigenetic Therapeutic Strategies in NB.

Class	Mechanism	Effects on NB	Immunologic Impact	Agents	References
**HDAC Inhibitors**	Inhibit histone deacetylation, increase chromatin accessibility	Promotes differentiation; reduces proliferation	↑ MHC-I expression; restores IFN signaling; enhances T/NK recognition	Vorinostat, Panobinostat, Entinostat	[64,103,104]
**DNMT Inhibitors**	Reduce DNA methylation, reactivate silenced genes	Restores tumor suppressors; reduces stem-like states	↑ Antigen presentation; ↑ chemokine signaling; sensitizes T_C_ killing	Decitabine, Azacitidine	[105,106]
**BET Inhibitors**	Block BRD4 binding to acetylated chromatin	Downregulates MYCN; disrupts survival pathways	↑ MHC-I; ↑ IFN pathways; ↓ immunosuppressive cytokines	JQ1, OTX015	[107,108,109,110]
**EZH2 Inhibitors (PRC2)**	Reduce H3K27me3-mediated repression	Promotes differentiation; weakens PRC2 survival circuits	↑ Antigen presentation; ↑ inflammatory signaling	Tazemetostat	[52,111,112]
**LSD1 Inhibitors**	Block H3K4/H3K9 demethylation	Reduces stemness; reactivates differentiation genes	↑ MHC-I; ↑ IFN responses; enhances T/NK cytotoxicity	Seclidemstat, Iadademstat	[113,114,115,116,117,118]
**PRC1 Inhibitors**	Inhibit BMI1/RING1B-mediated H2AK119ub1	Reduces self-renewal; promotes differentiation	↑ Immunostimulatory gene expression	BMI1 inhibitors (preclinical)	[119,120,121]
**HAT Activators**	Enhance histone acetylation and transcriptional activation	Restores lineage programs; counters repressive chromatin	↑ MHC-I; ↑ IFN signaling; improves immune visibility	Emerging HAT-stabilizing compounds	[122,123]
**PROTACs**	Recruit E3 ubiquitin ligase—target protein ubiquitination and proteasomal degradation	Suppress MYCN-driven transcriptional programs, reduced tumor proliferation,	↑ antigen-presentation, ↑ interferon signaling, ↓ immunosuppressive cytokines	PROTAC BRD4	[124,125]

**Table 3:** Ongoing and completed clinical trials evaluating CAR-T cell-based IMT for NB. Clinical studies investigating CAR-T cell therapies and related adoptive cellular IMTs in NB (ClinicalTrials.gov). The studies encompass a range of CAR-T strategies targeting tumor-associated antigens (e.g., GD2, B7-H3 (CD276), GPC2), as well as combination approaches involving NK cells, cytokine-modified constructs, or adjunct chemotherapeutic (e.g., fludarabine, cyclophosphamide) lymphodepletion regimens. Trials vary in phase and design, including early-phase safety and efficacy studies, and with relapsed, refractory, or HR disease.

Nct number	Study title	Study status	Interventions
Nct05990751	Multi-modular chimeric antigen receptor targeting GD2 in NB	Active not_ Recruiting	GD2 car t cells
Nct07007117	Phox2b pc-car t cells for relapsed NB	Recruiting	phox2b pc-car t cells
Nct06684639	Efficacy of GD2-CAR-T in treatment of NB	Recruiting	GD2-CAR-T cell
Nct05650749	Gpc2 CAR-T cells for relapsed/refractory NB	Recruiting	Gpc2 CAR-T cells
Nct02919046	Efficacy and safety with CAR-T for relapsed or refractory NB in children	Unknown	GD2-targeted CAR-T cells
Nct02311621	Engineered NB cellular immunotherapy (encit)-01	Active not_ Recruiting	patient derived CD171 specific Egfrt CAR-T cells—2nd GEN; 3rd GEN; long spacer 2nd GEN T cells
Nct04637503	4s CAR-T therapy targeting GD2, Psma and CD276 for treating NB	Unknown	GD2, Psma and CD276 CAR-T cells
Nct07172958	Selective antigen specific T_c_ and CAR-T cells in subjects with relapsed/refractory NB	Recruiting	Selective antigen specific dtî^2^rii-expressing T_c_ combined with B7-h3 CAR-T cells
Nct07087002	Gpc2-car t cell therapy for relapsed or refractory medulloblastoma in children and young adults	Recruiting	Gpc2- CAR-T cells|drug fludarabine; drug cyclophosphamide
Nct01460901	Donor derived, multi-virus-specific, cytotoxic t-lymphocytes for relapsed/refractory NB	Completed	GD2 car modified tri-virus specific cytotoxic T_c_
Nct07211737	Nkg2d.zeta-nk cell conditioning with c7r.GD2.CAR-T cells for relapsed or refractory NB	Not yet_ Recruiting	Genetic i15.nkg2d.zeta NK cells and c7r.GD2.CAR-T cells
Nct03373097	Anti-GD2 CAR-T cells for HR and/or relapsed/refractory NB	Active not_ Recruiting	GD2-cart01
Nct02107963	A phase I trial of T_c_ expressing an anti-GD2 CAR-T in children with GD2+ solid tumors	Completed	anti-GD2-car engineered t cells|drug ap1903|drug cyclophosphamide
Nct06836505	Safety and efficacy of CAR-T for relapsed/refractory NB a single-arm, open-label trial.	Recruiting	CAR-T therapy
Nct01953900	Ic9-GD2-car-vzv-ctls/refractory or metastatic GD2-positive sarcoma and NB	Active_not_ Recruiting	Genetic GD2 T_c_; vzv vaccine; drug fludarabine; drug cyclophosphamide
Nct04483778	B7H3 CAR-T cell immunotherapy for recurrent/refractory solid tumors in children	Active_not_ Recruiting	2nd GEN 4-1bbζ b7h3-egfrt-dhfr; 4-1bbζ b7h3-egfrt-dhfr (Sel); 4-1bbζ CD19-her2tg Pembrolizumab
Nct04897321	B7-H3-specific chimeric antigen receptor autologous T-cell therapy for pediatric patients with solid tumors (3car)	Recruiting	Drug fludarabine|drug cyclophosphamide|drug mesna|drug b7-H3 CAR-T cells
Nct03721068	CAR T-cells targeting the GD2 with il-15+icaspase9 for relapsed/refractory NB	Recruiting	ic9.GD2.car.il-15 T_c_; drug cyclophosphamide; drug fludarabine
Nct04864821	Clinical study of CD276 targeted autologous CAR-T cell infusion in patients with CD276 positive advanced solid tumor	Unknown	Drug targeting CD276 car t cells
Nct01822652	3rd GEN GD-2 chimeric antigen receptor and icaspase suicide safety switch, NB, grain	Active_not_ Recruiting	Genetic IC9-GD2 T_c_—frozen or fresh. Drug Cytoxan; fludara; Keytruda; genetic ic9-GD2 T_c_
Nct03294954	GD2 specific car and interleukin-15 expressing autologous NK T_c_ to treat children with NB	Recruiting	Genetic ginakit cells; ginakit cells + etanercept
Nct05562024	Taa06 injection in the treatment of patients with B7-H3-positive relapsed/refractory NB	Recruiting	T_c_ injection targeting b7-h3 chimeric antigen receptor
Nct02761915	A phase i trial of anti-GD2 T_c_ (1rg-cart)	Completed	Other leukapheresis|drug cyclophosphamide|drug fludarabine|genetic 1rg-cart
Nct04539366	Testing a new immune cell therapy, GD2 CAR-T, in children, adolescents, and young adults with relapsed/refractory NB, the GD2-car persist trial	Suspended	Procedure—biopsy; biospecimen; bone marrow aspiration; imaging heart etc. Drug cyclophosphamide; fludarabine; GD2-CAR-T
Nct00085930	Blood T_c_ and EBV specific ctls expressing GD2 CAR-T to NB patients	Completed	EBV specific ctls
Nct06803875	Study of halk.car t cells for patients with relapsed/refractory HR NB	Recruiting	autologous halk.car T_c_
Nct02439788	3rd GEN GD2 specific chimeric antigen receptor transduced autologous natural killer T_c_ for NB	Withdrawn	Drug cyclophosphamide|drug fludarabine|genetic ginakit cells
Nct03618381	Egfr806 car t cell imt for recurrent/refractory NB	Active_not_ Recruiting	2nd GEN 4-1bbζ egfr806-egfrt; 4-1bbζ egfr806-egfrt and 4-1bbζ CD19-her2tg
Nct07358260	B7-h3.CD28z.cart in solid tumors	Not_yet_ Recruiting	B7-H3.CD28z.cart|drug fludarabine|drug cyclophosphamide
Nct06500819	Autologous b7-h3 CAR T cells in relapsed/refractory NB	Recruiting	Drug b7-h3cart dose (intravenous)
Nct03635632	C7r-GD2 CAR-T cells for patients with relapsed or refractory NB	Active_not_ Recruiting	Genetic c7r-GD2.cart cells
Nct02765243	Anti-GD2 4th GEN cart cells targeting refractory and/or recurrent NB	Completed	anti-GD2 cart

## Data Availability

No new data were created or analyzed in this study.

## Abbreviations

NB	Neuroblastoma
TIME	Tumor Immune Evasion
HR-NB	High-risk Neuroblastoma
NCC	Neural Crest Cells
TME	Tumor Microenvironment
TAMs	Tumor-associated Macrophages
MDSCs	Myeloid-Derived Suppressor Cells
T_c_	T Lymphoid cells
DC	Dendritic Cells
NK	Natural Killer Cells
TDE	Tumor-Derived Exosomes
CT	Chemotherapy
RT	Radiotherapy
ASCT	Autologous Stem Cell Rescue
IMT	Incorporation of Immunotherapy
OS	Overall Survival
MRD	Minimal Residual Disease
MHC	Major Histocompatibility Complex
CSC	Cancer Stem Cell
scRNA-seq	Single Cell RNA Sequence
NO	Nitric Oxide
TCR	T Lymphoid cells Receptor
HIF	Hypoxia-Inducible Factor
CTL	Cytotoxic T Lymphocyte
APM	Antigen Processing Machinery
eAdo	Extracellular Adenosine
A2AR	A2A Adenosine Receptor
APCs	Antigen-Presenting Cells
Treg	T-regulatory cell
FAO	Fatty Acid Oxidation
OXPHOS	Oxidative Phosphorylation
GPX4	Glutathione Peroxidase 4
HDAC	Histone Deacetylase
HDACi	HDAC inhibitors
DNMT	DNA Methyltransferase
DNMTi	DNMT inhibitors
BET	Bromodomain and extraterminal domain
BETi	BET inhibitors
LSD1	Lysine specific demethylase 1
LSD1i	LSD1 inhibitors
PRC1	Polycomb Repressive Complex 1
HAT	Histone Acetyltransferase
PROTACs	Proteolysis-Targeting Chimeras
EVs	Extracellular Vehicles
MVB	Endosomal-Multivesicular Body
IL-2	Interleukin-2
PGE2	Prostaglandin E2
ARG1	Arginase-1
iNOS	Inducible Nitric Oxide Synthase
CSC-Exos	CSC-derived Exosomes
ADCC	Antibody-Dependent Cellular Cytotoxicity
CAR	Chimeric Antigen Receptor
NK-Exos	NK-cell-derived Exosomes
BsAbs	Bispecific Antibodies
Ovs	Oncolytic Viruses
